# Molecular dynamics simulation or structure refinement of proteins: are solvent molecules required? A case study using hen lysozyme

**DOI:** 10.1007/s00249-022-01593-1

**Published:** 2022-03-18

**Authors:** Maria Pechlaner, Wilfred F. van Gunsteren, Niels Hansen, Lorna J. Smith

**Affiliations:** 1 Laboratory of Physical Chemistry, Swiss Federal Institute of Technology, ETH, 8093 Zurich, Switzerland; 2 Institute of Thermodynamics and Thermal Process Engineering, University of Stuttgart, 70569 Stuttgart, Germany; 3 Inorganic Chemistry Laboratory, Department of Chemistry, University of Oxford, South Parks Road, Oxford, OX1 3QR UK

**Keywords:** Stochastic dynamics simulation, Structure refinement, Implicit solvation, Mean solvation force, Conformational sampling

## Abstract

In protein simulation or structure refinement based on values of observable quantities measured in (aqueous) solution, solvent (water) molecules may be explicitly treated, omitted, or represented by a potential of mean-solvation-force term, depending on protein coordinates only, in the force field used. These three approaches are compared for hen egg white lysozyme (HEWL). This 129-residue non-spherical protein contains a variety of secondary-structure elements, and ample experimental data are available: 1630 atom–atom Nuclear Overhauser Enhancement (NOE) upper distance bounds, 213 ^*3*^* J*-couplings and 200 *S*^*2*^ order parameters. These data are used to compare the performance of the three approaches. It is found that a molecular dynamics (MD) simulation in explicit water approximates the experimental data much better than stochastic dynamics (SD) simulation in vacuo without or with a solvent-accessible-surface-area (SASA) implicit-solvation term added to the force field. This is due to the missing energetic and entropic contributions and hydrogen-bonding capacities of the water molecules and the missing dielectric screening effect of this high-permittivity solvent. Omission of explicit water molecules leads to compaction of the protein, an increased internal strain, distortion of exposed loop and turn regions and excessive intra-protein hydrogen bonding. As a consequence, the conformation and dynamics of groups on the surface of the protein, which may play a key role in protein–protein interactions or ligand or substrate binding, may be incorrectly modelled. It is thus recommended to include water molecules explicitly in structure refinement of proteins in aqueous solution based on nuclear magnetic resonance (NMR) or other experimentally measured data.

**Supplementary Information:**

The online version contains supplementary material available at 10.1007/s00249-022-01593-1.

## Introduction

Since the first simulations of the dynamics of a protein more than four decades ago (McCammon et al. 1977; van Gunsteren and Berendsen 1977), the application of molecular dynamics (MD) simulation to proteins has seen a continuous development in terms of accuracy and efficiency and the use of MD simulation has spread through chemistry, biochemistry and molecular biology (van Gunsteren and Berendsen 1990; van Gunsteren et al. 2006, 2018). The first protein simulations involved a simple protein model (454 united or extended atoms) of the protein bovine pancreatic inhibitor (BPTI, 58 residues), without any non-polar hydrogen atoms or solvent molecules, that is, using a vacuum boundary condition. A short non-bonded interaction cut-off distance (*R*_*c*_ = 0.8 nm) was employed and the relative dielectric permittivity *ε*_*r*_ was assumed to be proportional to the distance *r* between the atoms of the protein. These conditions limited the accuracy of the simulations. In the next decades, larger proteins were simulated, the protein models were refined, more hydrogen atoms and water molecules were added, the non-bonded interaction cut-off was extended (e.g. to *R*_*c*_ = 1.4 nm), and long-ranged electrostatic interactions were modelled using continuum electrostatics or lattice periodicity. These improvements of the models, in particular the addition of many water molecules, easily ten thousand to solvate a protein in a periodic box, required an increased computing effort. As a consequence, it was attempted to simplify the models again, for example by coarse-graining, that is, representing multiple atoms by a single interaction centre or bead (Riniker et al. 2012), or by replacing the explicit treatment of the solvating water molecules by a mean solvation force that is a function of the positions $\vec{r}^{N} \equiv (\vec{r}_{1} ,\vec{r}_{2} ,...,\vec{r}_{N} )$ in Cartesian coordinates of the *N* protein atoms. In such an implicit-solvation model (van Gunsteren et al. 1994), the influence of the solvent on the protein degrees of freedom is incorporated in the interaction function and equations of motion of the latter in an average manner.

### Modelling solvent effects upon protein structure

The solvent effect upon the structure and dynamics of a solute may be divided into different types.

1. The average or mean interaction between solute atoms is affected by the presence of solvent. When the solvent is omitted from the simulation, the solute force field should be changed to incorporate the mean solvent effect, that is, a potential of mean force should be used for the solute.

2. The solvent exerts a dynamical effect on the solute, which may be mimicked by the introduction into the equations of motion of a frictional force representing solvent drag and of a stochastic force $\vec{f}_{i}^{\rm st} (t)$, randomly fluctuating in time *t*, representing collisions of solute atoms *i* with solvent molecules. In the simplest case, the frictional force is taken to be proportional to the velocity $\vec{v}_{i} (t)$ of the solute atom to which it applies, and the stochastic force $\vec{f}_{i}^{\rm st} (t)$ is a stationary Gaussian-distributed random variable, uncorrelated between the different degrees of freedom, which leads to the Langevin equation of motion, a stochastic ordinary differential equation,

1 $$m_{i} \dot{\vec{v}}_{i} (t) = \vec{f}_{i} (\vec{r}^{N} (t)) - m_{i} \gamma_{i} \vec{v}_{i} (t) + \vec{f}_{i}^{\rm st} (t),$$

where *m*_*i*_ is the mass and *γ*_*i*_ the friction coefficient of particle *i* (*i*=,2…,*N*), and a time derivative of a quantity is indicated by a dot over the symbol. The stochastic force is assumed to be a stationary Gaussian-distributed random variable with zero mean and to have neither correlation with prior velocities nor with the force

2 $$\vec{f}_{i}\,\, (\vec{r}^{N} (t)) = - \frac{{\partial V(\vec{r}^{N} (t))}}{{\partial \vec{r}_{i} (t)}},$$

as derived from the potential energy function $V(\vec{r}^{N} )$,

3 $$< f_{i}^{\rm st} (t^{\prime})\,f_{j}^{\rm st} (t) > = 2m_{i} \gamma_{i} k_{B} T_{ref} \delta_{ij} \delta (t - t^{\prime}),$$

4 $$P(f_{i}^{\rm st} ) = (2\pi < (f_{i}^{\rm st} )^{2} > )^{ - 1/2} \exp ( - (f_{i}^{\rm st} )^{2} /(2 < (f_{i}^{\rm st} )^{2} > )),$$

5 $$< f_{i}^{\rm st} > = 0,$$

6 $$< v_{i} (t^{\prime})f_{j}^{\rm st} (t) > = 0, \quad t \ge \, t^{\prime},$$

7 $$< f_{i} (t^{\prime})f_{j}^{\rm st} (t) > = 0, \quad t \ge \, t^{\prime},$$

where < … > denotes averaging over an equilibrium ensemble, *k*_*B*_ is Boltzmann’s constant, *T*_*ref*_ is the reference temperature, $P(f_{i}^{\rm st} )$ is the probability distribution of the stochastic force, *δ*_*ij*_ is the Kronecker delta and *δ(t-t’)* is the delta function. Note that Eqs. (3–7) are not formulated in terms of three-dimensional vectors $\vec{v}_{i}$ and $\vec{f}_{i}$, but in terms of their components (indicated by *v* and *f* without vector arrow), i.e. along the *x*-, *y*- and *z*-directions of the right-handed Cartesian coordinate system. A minor correction to Eq. (3) has been discussed in (Ciccotti and Ryckaert 1981; Bossis et al. 1982; van Gunsteren and Berendsen 1982).

The stochastic force *f*_*i*_^*st*^*(t)* and the atomic friction coefficient *γ*_*i*_ will only be sizable for protein atoms at the surface. Therefore, they are taken dependent on the number of neighbour atoms within the protein (Shi et al. 1988)

8 $$\gamma_{i} \left( t \right) \, = \gamma_{{{\text{solv}}}} \omega_{i} \left( t \right)$$

with

9 $$\omega_{i} \left( t \right) = {\text{ max}}\left( {0,{ 1} - N_{i}^{nb} (t)/N^{{n{\text{bref}}}} } \right),$$

where *N*_*i*_^*nb*^*(t)* denotes the number of non-hydrogen neighbour atoms of the protein atom *i* within 0.3 nm radius, and *N*^*nbref*^ is defined as an upper limit of 6 neighbour protein atoms at which solvent forces on solute atom *i* are assumed to vanish. For water as solvent (at room temperature and pressure) *γ*_*solv*_ = 91 ps^−1^, and *ω*_*i*_*(t)* is updated every 1 ps during the simulation (Shi et al. 1988).

### Elimination of protein or solvent degrees of freedom

The conditions that must be fulfilled by degrees of freedom in order that they may be eliminated in a physically correct manner in the process of model simplification, such that a computationally efficient and yet accurate coarse-grained model is obtained, are:

1. They must be non-essential for the process or property of interest.
2. They must be large in number or computationally intensive, so that the computational gain is substantial enough to off-set the loss in accuracy.
3. The interactions governing the degrees of freedom to be eliminated should be largely decoupled from the interactions governing the other degrees of freedom of the system which are to be maintained. This means that the frequency components of the motion along the degrees of freedom to be eliminated must be well separated from the other frequencies occurring in the system, and that the coupling between the two types of motion is weak (van Gunsteren and Berendsen 1977).
4. Their elimination should allow a simple, efficient representation of the interaction governing the other, remaining degrees of freedom.

### Use of an implicit-solvation model

The use of an implicit solvent model, i.e. the attempt to mimic the effect of the solvent by a function that is only dependent on the solute coordinates, does not satisfy conditions 3 and 4. If the solvent is water, it leads to various distortions of the interactions within the solute–solvent system:

1. The energy and entropy contributions of the solvent molecules to the free energy of the system are missing. For example, the experimental value of the excess free energy of liquid water at room temperature and pressure is with 24 kJ mol^−1^ about half the size of its heat of vaporization of 44 kJ mol^−1^. Thus, *TΔS*, where *T* is the temperature and *ΔS* the difference in entropy between gas and liquid phase, is about 20 kJ mol^−1^, and therefore, not negligible. While the energy contribution of the solvent molecules may to some extent be incorporated into the potential of mean force of the solute, the entropy contribution cannot, because it depends on the mobility of the solvent molecules.
2. Since a water molecule may serve as hydrogen-bond donor as well as hydrogen-bond acceptor, hydrogen bonding between solute and solvent is missing, leading to enhanced solute–solute hydrogen bonding (Shi et al. 1988).
3. Since the relative dielectric permittivity *ε*_*r*_ of water at room temperature and pressure is about 80, the dielectric screening effect of the aqueous solution is missing, leading to too strong electrostatic interactions.

Although the motions of a large solute may cover time scales ranging from femtoseconds to milliseconds and the relaxation times of water molecules are of the order of picoseconds, their motions on picosecond to nanosecond time scales are not decoupled, and thus condition 3 is not satisfied for some processes. In explicit water, the non-polar particles aggregate, and the electrostatic interaction between ions is reduced, leading to dissolution. So-called hydrophobic or non-polar particles do like water, but their interaction with water is less strong than the interaction of water with itself, leading to water excluding the hydrophobic molecules and their subsequent aggregation. Ions with opposite charges do like water more than each other, which leads to water surrounding the ions and dissolution of ion pairs. The ‘‘hydrophobic effect’’, the apparent attraction between non-polar molecules or repulsion between ions in aqueous solution due to the stronger interaction between the water molecules or between water molecules and ions, cannot be properly modelled in terms of solute and ion coordinates only, because the effective interaction between solute atoms and their entropy is a complex function of the distribution of solvent coordinates. Thus, also condition 4 is difficult to meet (Müller et al. 2006).

The mentioned fundamental inadequacies of implicit-solvation models (van Gunsteren et al. 1994; Müller et al. 2006) are inherent to any such model (e.g. generalized Born surface area (GBSA) models) and cannot be resolved by using one or the other parameter-calibration procedure when developing such a model.

In light of these considerations, one may wonder for which applications of implicit-solvation models the gain in computational efficiency outweighs the loss of accuracy and physical mechanisms. It makes definitively little sense in simulations of protein folding. If the solvent is omitted, folding is reduced to a problem of chain enthalpy and entropy. In implicit-solvation models, changes in solvent entropy in the first solvation shells upon folding and unfolding cannot be directly modelled in a potential energy term for the solute (Daura et al. 1999). In contrast, the omission of solvent molecules is common practice in structure determination and refinement of proteins based on experimental data. Whether this approximation is warranted will depend on the ratio of the number of independent measured values of observable quantities for a molecule using a particular measurement technique and the number of independent molecular degrees of freedom.

### Use of measured data to derive or refine protein structure

All techniques to derive structural information on (macro)molecules from the measurement of observable quantities *Q* make use of a relation of *Q* to structure $\vec{r}^{N}$, a function $Q(\vec{r}^{N} )$ (van Gunsteren et al. 2016). Virtually, all experimental techniques measure an average < *Q* > _*space,time*_ of *Q* over the molecules (space) in the test tube or in a crystal and over a time window determined by the type of experiment, which may range from picoseconds to seconds. This means that < *Q* > constitutes an average over a Boltzmann-weighted set, i.e. a statistical-mechanical ensemble, of molecular configurations. The weights are proportional to $\exp ( - V(\vec{r}^{N} )/k_{B} T)$, where $V(\vec{r}^{N} )$ indicates the energy of a molecular configuration or structure $\vec{r}^{N}$.

The quality of a set of structures $\vec{r}^{N}$ derived from a set of measured values *Q*^exp^ of *Q* using a particular molecular model will depend on various factors of the structure determination procedure.

1. *Q*^exp^ values are subject to uncertainty or error.
2. It is not possible to fully account for the averaging over space and time inherent in the experimental measurement.
3. For most bio-molecular systems, the number of independent *Q*^exp^ values available, *N*^exp^, is much smaller than the number of degrees of freedom of the system, *N*^dof^. This means that the structure determination problem is underdetermined (*N*^exp^/*N*^dof^ <  < 1) and can only be addressed using a molecular model, i.e. a function $V(\vec{r}^{N} )$ specifying likely structural parameters (e.g. bond lengths and bond angles) of a system. The function $V(\vec{r}^{N} )$ may yield low-energy values for configurations that are physically most reasonable. The fewer *Q*^exp^ values that are available or the lower *N*^exp^/*N*^dof^, the larger the influence of the choice of molecular model and interaction function $V(\vec{r}^{N} )$ and its inaccuracy, for example due to omission of solvent molecules, on the generated structures will be.
4. The function $Q(\vec{r}^{N} )$ is not known or it involves assumptions or approximations affecting its accuracy.
5. The inverse function $\vec{r}^{N} (Q)$ of the function $Q(\vec{r}^{N} )$ may not exist, or if it does, it may be multiple-valued.
6. The generation or sampling of molecular configurations $\vec{r}^{N}$ must be biased, i.e. guided towards those that are (on average) compatible with *Q*^exp^. This is particularly challenging when the inverse function $\vec{r}^{N} (Q)$ of the function $Q(\vec{r}^{N} )$ is multiple-valued.

### Information density of various experimental data for proteins

The third factor involves the balance between the quality or accuracy of the molecular model used and the number of independent experimental values *Q*^exp^ available for a quantity *Q*, which is very different for different experimental measurement techniques, such as X-ray diffraction, NMR, CD, Raman or infrared spectroscopy. Where X-ray diffraction of crystals is a measurement technique that is characterised by a high information density, that is, a large ratio *N*^exp^/*N*^dof^ of the number of independent measured values of observable quantities for a molecule and the number of independent molecular degrees of freedom, NMR measurements of proteins in aqueous solution show a much lower information density, and circular dichroism (CD), Raman or infrared spectroscopy or small-angle X-ray scattering (SAXS) data have a very low information density. This implies that the omission of solvent in a crystalline system when deriving protein structure from abundant X-ray diffraction intensities requires a less accurate molecular model than the omission of solvent when deriving protein structure from much less abundant NMR data for a protein in solution. In the latter case, the use of an implicit-solvation model instead of the complete neglect of solvating water molecules may improve the quality of the obtained structures.

### Experimental data are generally averages over protein conformations

The second above-mentioned factor also plays a different role when applying the different mentioned measurement techniques. For observable quantities *Q*, such as X-ray diffraction intensities *I*_*hkl*_, Nuclear Overhauser Enhancement intensities (NOEs; when represented as atom–atom distance bounds), ^*3*^* J*-couplings or chemical shifts, it is possible to formulate a function $Q(\vec{r}^{N} )$ relating a *Q*-value to a particular structure $\vec{r}^{N}$. For other observable quantities, such as *S*^*2*^ order parameters or residual dipolar couplings (RDCs), the function relating *Q* to $\vec{r}^{N}$ involves some average over the Boltzmann ensemble of structures in solution, $Q( < f(\vec{r}^{N} ) > )$, where *f* denotes the function of $\vec{r}^{N}$ that is being averaged (van Gunsteren et al. 2016). This means that structure determination or refinement based on such quantities must involve the averaging $< f(\vec{r}^{N} ) >$ in addition to the averaging $< Q( < f(\vec{r}^{N} ) > ) >$. Unfortunately, an RDC value is the result of the averaging of a dipolar coupling over the rotational motion of the molecule. For a protein in aqueous solution, the extensive sampling of its rotational motion in an MD simulation would easily take microseconds, which would make protein structure determination based on RDCs using explicit water molecules in the simulation rather expensive, whereas the use of a computationally efficient implicit-solvation model would allow simulations of microseconds length.

**Fig. 1:**
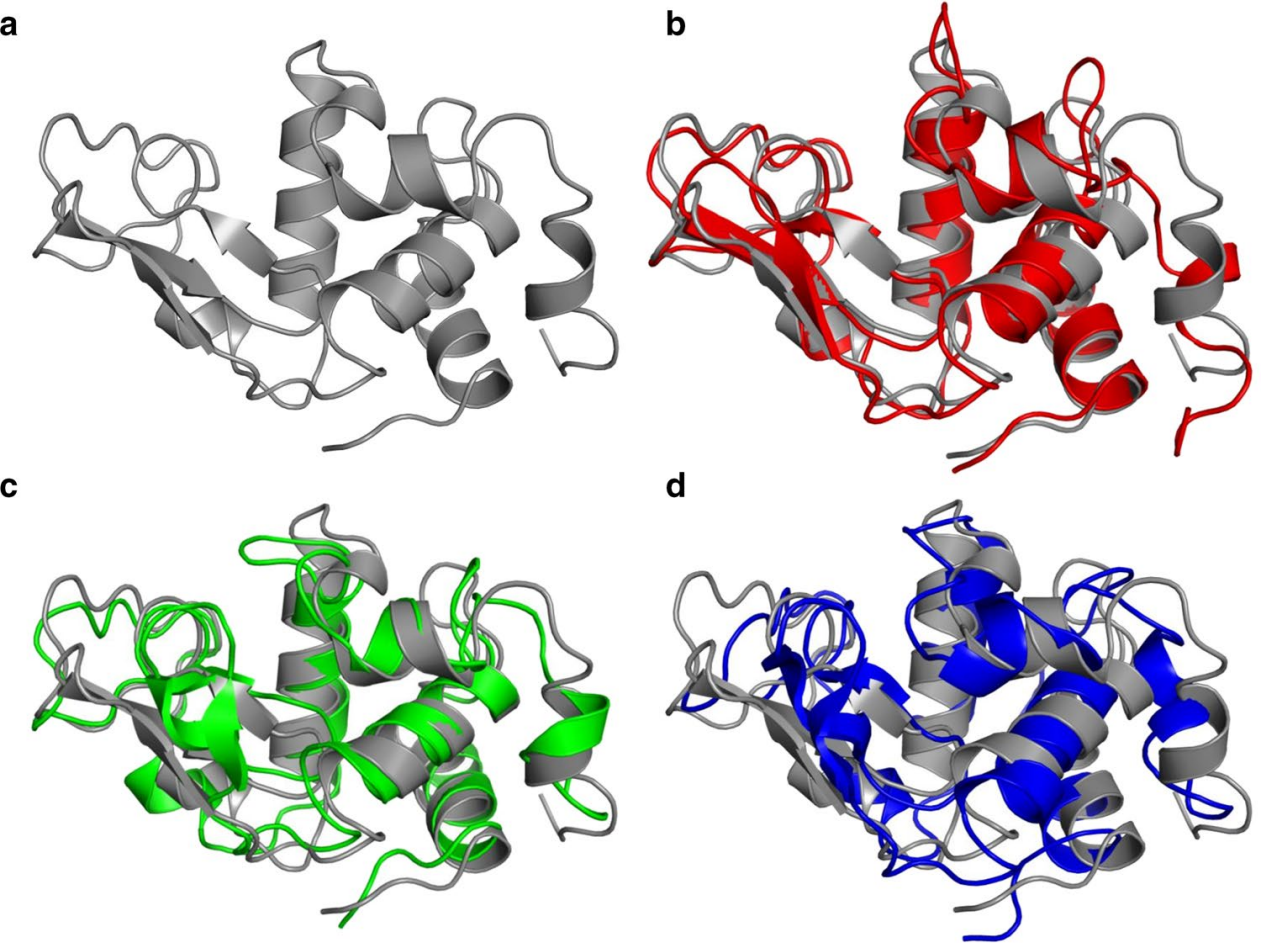
Ribbon pictures of the structure of HEWL. **a** *2VB1* X-ray structure (grey). **b** Final structure of the MD_water simulation (red) overlayed on the 2VB1 X-ray structure. **c** Final structure of an SD_nowater simulation (green) overlayed on the 2VB1 X-ray structure. **d** Final structure of an SD_implicit simulation (blue) overlayed on the 2VB1 X-ray structure

In the present article, it is investigated whether the complete omission of solvent or the use of an implicit-solvation model in an SD simulation of a protein will lead to larger deviations from experimental data than the use of explicit solvent molecules in an MD simulation using periodic boundary conditions. The protein hen egg white lysozyme (HEWL, 129 amino-acid residues), see Fig. 1, serves as test molecule, because ample NMR data of this protein in solution are available.

## Methods

Energy minimisations, molecular dynamics and stochastic dynamics simulations were performed using the GROMOS bio-molecular simulation software (Schmid et al. 2011a, 2012; van Gunsteren et al. 2019a).

### Molecular model for the MD simulation in explicit water using periodic boundary conditions

When solvated in explicit water (MD_water), the protein was modelled using the GROMOS bio-molecular force field 54A7 (Poger et al. 2010; Schmid et al. 2011b). In view of the pH used in the experimental NMR measurements, pH⁓3.8, only Glu 35 was protonated and His was doubly protonated (Bartik et al. 1994). The simple-point-charge (SPC) model (Berendsen et al. 1981) was used to describe the solvent molecules in the rectangular periodic box. To compensate for the overall positive charge of the protein, 10 Cl^−^ ions were included in the solution. All bond lengths and the bond angle of the water molecules were kept rigid with a relative geometric precision of 10^–4^ using the SHAKE algorithm (Ryckaert et al. 1977), allowing for a 2 fs MD time step in the leap-frog algorithm (Hockney and Eastwood 1981) used to integrate the equations of motion. For the non-bonded interactions, a triple-range method (van Gunsteren et al. 1986) with cut-off radii of 0.8/1.4 nm was used. Short-range (within 0.8 nm) van der Waals and electrostatic interactions were evaluated every time step based on a charge-group pair list (van Gunsteren et al. 2019b). Medium-range van der Waals and electrostatic interactions, between pairs at a distance larger than 0.8 nm and shorter than 1.4 nm, were evaluated every fifth time step (10 fs), at which time point the pair list was updated, and kept constant between updates. Outside the larger cut-off radius (1.4 nm) a reaction-field approximation (Barker and Watts 1973; Tironi et al. 1995) with a relative dielectric permittivity of 61 (Heinz et al. 2001) was used. Minimum-image periodic boundary conditions were applied.

### Molecular model for the SD simulations in vacuo without and with implicit-solvation term

When simulating the protein in vacuo (SD_nowater, SD_implicit), the GROMOS bio-molecular force field 54B7 (van Gunsteren and Dolenc 2012, van Gunsteren et al. 2019c) was used. The A-version of a GROMOS force field is the basic force field designed for molecules in explicit water. The B-version is derived from the A-version in order to be used for simulating molecules in vacuo, where the dielectric screening effect of the environment is neglected. The atomic charges and van der Waals parameters are changed such that atom charge groups with a non-zero total charge are neutralized while maintaining the hydrogen-bonding capacity of the individual atoms. This takes account of the dielectric screening of the aqueous solution that is missing in vacuo.

All bond lengths were kept rigid with a relative geometric precision of 10^–4^ using the SHAKE algorithm (Ryckaert et al. 1977), allowing for a 2 fs MD time step in the leap-frog algorithm (van Gunsteren and Berendsen 1988) used to integrate the Langevin equation of motion. The non-bonded interactions were treated as in the MD simulation in explicit water. No periodic boundary conditions were applied.

The implicit-solvation term of the force field is of the so-called solvent-accessible-surface-area (SASA) type, in which the local solute–solvent interactions are assumed to be proportional to the SASA of the solute atoms (Chothia 1974; Eisenberg and McLachlan 1986). The implicit-solvation term with parameter values that make it compatible with the GROMOS force fields reads (Ooi et al. 1987; Still et al. 1990; Fraternali and van Gunsteren 1996; Kunz et al. 2012; Kleinjung et al. 2012)

10 $$V_{solv}^{SASA} (\vec{r}^{N} ) \equiv \sum\limits_{i = 1}^{N} {\sigma_{i}^{SASA} A_{i} (} \vec{r}^{N} ),$$

where the weight factors $\sigma_{i}^{SASA}$ are the implicit-solvation model parameters for the atoms of the molecule, which may differ per atom type or only between classes of atoms, such as charged, polar or non-polar atoms (Kleinjung et al. 2012), see Table 1. The accessible area $A_{i} (\vec{r}^{N} )$ of atom *i* is defined using the approximate analytical expression (Hasel et al. 1988).

11 $$A_{i} (\vec{r}^{N} ) \equiv S_{i} \prod\limits_{j = 1,j \ne i}^{N} {\left[ {1 - p_{i} p_{ij} b_{ij} (r_{ij} )/S_{i} } \right]} .$$

**Table 1:** Two sets of implicit-solvation model parameters compatible with the GROMOS 54B7 force field (Kleinjung et al. 2012)

Code	Name	*R*_*i*_	*p*_*i*_	σ_i_^SASA^	Atom type	σ_i_^SASA^	Description
		nm		kJmol^−1^ nm^−2^		kJmol^−1^ nm^−2^	
1	O	0.150	0.926	− 7.2	Polar	− 7.3	Carbonyl oxygen (C=O)
2	OM	0.170	0.922	− 21.7	Charged	− 23.3	Carboxyl oxygen (CO^−^)
3	OA	0.152	1.080	− 7.0	Polar	− 7.3	Hydroxyl or sugar oxygen
4	OE	0.152	1.080	–	–	–	Ether or ester oxygen
5	OW	−	−	–	–	–	Water oxygen
6	N	0.155	1.028	0.0	−	0.0	Peptide nitrogen (NH)
7	NT	0.160	1.215	− 4.0	Polar	− 7.3	Terminal nitrogen (NH2)
8	NL	0.160	1.215	− 26.1	Charged	− 23.3	Terminal nitrogen (NH3)
9	NR	0.155	1.028	− 4.5	Polar	− 7.3	Aromatic nitrogen
10	NZ	0.155	1.028	− 13.3	Charged	− 23.3	Arg NH (NH2)
11	NE	0.155	1.028	0.0	–	0.0	Arg NE (NH)
12	C	0.172	1.554	0.0	−	0.0	Bare carbon
13	CH0	0.172	1.554	–	–	–	Bare sp3 carbon, 4 bound heavy atoms
14	CH1	0.180	1.276	3.8	hydrophobic	4.1	Aliphatic or sugar CH-group
15	CH2	0.190	1.045	5.0	hydrophobic	4.1	Aliphatic or sugar CH2-group
16	CH3	0.200	0.880	3.3	hydrophobic	4.1	Aliphatic CH3-group
17	CH4	–	–	–	–	–	Methane
18	CH2r	0.190	1.045	–	–	–	Aliphatic or sugar CH2-group in ring
19	CR1	0.180	1.073	4.5	hydrophobic	4.1	Aromatic CH-group
20	HC	0.110	1.128	0.0	–	–	Hydrogen bound to carbon
21	H	0.110	1.128	0.0	–	–	Hydrogen not bound to carbon
22	DUM	–	–	–	–	–	Dummy atom
23	S	0.180	1.121	0.0	–	–	Sulphur

Values in the fifth column were taken from Table 1 of (Kleinjung et al. 2012), which cover the GROMOS non-bonded interaction atom types (first column: integer atom code; second column: atom name) for proteins (van Gunsteren et al. 2019b). The values in the seventh column were taken from Table 2 of (Kleinjung et al. 2012), which contains a simplified set of parameters based on only three types of atoms. Values of *R*_*i*_ and *p*_*i*_ were taken from (Hasel et al. 1988)

Here, the total surface area of an isolated atom *i* with radius *R*_*i*_ accessible to a solvent probe atom with radius *R*_*solv*_ is given by

12 $$S_{i} \equiv 4\pi \left( {R_{i} + R_{solv} } \right)^{2}$$

and the overlap reduction factor *b*_*ij*_ (Wodak and Janin 1980) for atoms *i* and *j* at a distance $r_{ij} \equiv ((\vec{r}_{i} - \vec{r}_{j} )^{2} )^{1/2}$ is given by

13a $$b_{ij} (r_{ij} ) \equiv \pi (R_{i} + R_{solv} )(R_{i} + R_{j} + 2R_{solv} - r_{ij} )(1 + (R_{j} - R_{i} )/r_{ij} ),$$

if 0 < *r*_*ij*_ < *R*_*i*_ + *R*_*j*_ + 2*R*_*solv*_, and

13b $$b_{ij} (r_{ij} ) \equiv 0,$$

if *r*_*ij*_ ≥ *R*_*i*_ + *R*_*j*_ + 2*R*_*solv*_.

The atom type parameter *p*_*i*_ has been introduced in Eq. (11) to empirically reduce the effect of double counting the overlap area when multiple overlaps of the surface of atom *i* with those of many other atoms *j* occur. The pair parameter *p*_*ij*_ serves as an additional reducing factor that distinguishes between first and next covalently bound neighbour atoms *j* of atom *i*. The parameters *p*_*i*_ and *p*_*ij*_ (*p*_*ij*_ = 0.8875 for covalently bound first neighbours and *p*_*ij*_ = 0.3516 for covalently bound next neighbours) have been optimized (Hasel et al. 1988) using *R*_*solv*_ = 0.14 nm and given *R*_*i*_ values to reproduce the exact solvent-accessible surface areas of a large number of small molecules. The values are given in Table 1 of (Hasel et al. 1988) and their mapping onto the atom types used in the GROMOS 54B7 force field, along with the corresponding *R*_*i*_ values, is given in Table 1.

The force on atom *k* resulting from the implicit-solvation potential energy term $V_{solv}^{SASA} (\vec{r}^{N} )$, Eq. (10), is

14 $$\vec{f}_{k}^{SASA} = - \frac{{\partial V_{solv}^{SASA} (\vec{r}^{N} )}}{{\partial \vec{r}_{k} }} = - \sum\limits_{i = 1}^{N} {\sigma_{i}^{SASA} \frac{{\partial A_{i} (\vec{r}^{N} )}}{{\partial \vec{r}_{k} }}} ,$$

with

15 $$\frac{{\partial A_{i} (\vec{r}^{N} )}}{{\partial \vec{r}_{k} }} = S_{i} \left( { - p_{i} p_{ik} \frac{{\partial b_{ik} (r_{ik} )}}{{\partial \vec{r}_{k} }}/S_{i} } \right)\prod\limits_{j = 1,j \ne i,j \ne k}^{N} {\left( {1 - p_{i} p_{ij} b_{ij} (r_{ij} )/S_{i} } \right)} ,\,{\text{if}}\;k \ne i,$$

and

16 $$\frac{{\partial A_{i} (\vec{r}^{N} )}}{{\partial \vec{r}_{i} }} = S_{i} \sum\limits_{l = 1,l \ne i}^{N} {\left( { - p_{i} p_{il} \frac{{\partial b_{il} (r_{il} )}}{{\partial \vec{r}_{i} }}/S_{i} } \right)} \prod\limits_{j = 1,j \ne i,j \ne l}^{N} {\left( {1 - p_{i} p_{ij} b_{ij} (r_{ij} )/S_{i} } \right)} ,\,{\text{if}}\;k = i.$$

The partial derivatives of the overlap reduction factor *b* can be written as

17 $$\frac{{\partial b_{ik} (r_{ik} )}}{{\partial \vec{r}_{k} }} = \frac{{db_{ik} (r_{ik} )}}{{dr_{ik} }}\frac{{\partial r_{ik} }}{{\partial \vec{r}_{k} }},$$

and

18 $$\frac{{\partial b_{ik} (r_{ik} )}}{{\partial \vec{r}_{i} }} = \frac{{db_{ik} (r_{ik} )}}{{dr_{ik} }}\frac{{\partial r_{ik} }}{{\partial \vec{r}_{i} }},$$

with

$$\frac{{db_{ik} (r_{ik} )}}{{dr_{ik} }} = - \pi (R_{i} + R_{solv} )(1 + (R_{k} - R_{i} )/r_{ik} )$$

19a $$+ \pi (R_{i} + R_{solv} )( - (R_{k} - R_{i} )/r_{ik}^{2} )(R_{i} + R_{k} + 2R_{solv} - r_{ik} ),$$

if 0 < *r*_*ij*_ < *R*_*i*_ + *R*_*k*_ + 2*R*_*solv*_, and

19b $$\frac{{db_{ik}\,\, (r_{ik} )}}{{dr_{ik} }} = 0,$$

if *r*_*ij*_ ≥ *R*_*i*_ + *R*_*k*_ + 2*R*_*solv*_, and

20 $$\frac{{\partial \,\,r_{ik} }}{{\partial \vec{r}_{k} }} = - \frac{{\vec{r}_{ik} }}{{r_{ik} }},$$

and

21 $$\frac{{\partial r_{ik} }}{{\partial \vec{r}_{i} }} = \frac{{\vec{r}_{ik} }}{{r_{ik} }},$$

with $$\vec{r}_{ik} \equiv \vec{r}_{i} - \vec{r}_{k}$$ and $$r_{ik} \equiv ((\vec{r}_{ik} )^{2} )^{1/2}$$.

The parameters $\sigma_{i}^{SASA}$ of the implicit-solvation model that are compatible with the GROMOS 54B7 force field were taken from (Kleinjung et al. 2012) and are given in Table 1.

### Simulation set-up for the MD simulation in explicit water using periodic boundary conditions

The X-ray crystal structure derived from a triclinic unit cell at 0.065 nm resolution at *T* = 100 K with Protein Data Bank (PDB) code *2VB1* (Berman et al. 2000) was used as the initial structure for the energy minimisations followed by the MD and SD simulations. It contains multiple side-chain conformations for 46 residues. For the initial structure, the side-chain conformation with the highest occupancy was chosen.

The initial structure was first energy minimised in vacuo to release possible strain induced by small differences in bond lengths, bond angles, improper dihedral angles, and short distance non-bonded contacts between the force-field parameters and the X-ray structure. Subsequently, the protein was put into a rectangular box filled with water molecules, such that the minimum solute-wall distance was 1.0 nm, and water molecules closer than 0.23 nm from the solute were removed. This resulted in a box with 12,157 water molecules for the initial protein structure. To relax unfavourable contacts between atoms of the solute and the solvent, a second energy minimisation was performed for the protein in the periodic box with water while keeping the atoms of the solute harmonically position-restrained (van Gunsteren et al. 2019b) with a force constant of 25,000 kJmol^−1^ nm^−2^ (Lier et al. 2020).

The resulting protein-water configuration was used as initial configuration for the MD simulation in explicit water. To avoid artificial deformations in the protein structure due to relatively high-energy atomic interactions still present in the system, the MD simulation was started at *T* = 60 K and then the temperature was slowly raised to *T* = 308 K. Initial atomic velocities were sampled from a Maxwell distribution at *T* = 60 K. The equilibration scheme consisted of five short 20 ps simulations at temperatures 60, 120, 180, 240 and 308 K at constant volume. During the first four of the equilibration periods, the solute atoms were harmonically restrained to their positions in the initial structures with force constants of 25,000, 2500, 250, and 25 kJmol^−1^ nm^−2^. The temperature was kept constant using the weak-coupling algorithm (Berendsen et al. 1984) with a relaxation or coupling time *τ*_*Τ*_ = 0.1 ps. Solute and solvent were separately coupled to the heat bath. Following this equilibration procedure, the simulations were performed at a reference temperature of 308 K and a reference pressure of 1 atm. The pressure was kept constant using the weak-coupling algorithm (Berendsen et al. 1984) with a coupling time *τ*_*p*_ = 0.5 ps and an isothermal compressibility *κ*_*T*_ = 4.575 10^–4^ (kJmol^−1^ nm^−3^)^−1^. The centre of mass motion of the system was removed every 1000 time steps (2 ps). Trajectory energies and atomic coordinates were stored at 5 ps intervals and used for analysis (Eichenberger et al. 2011).

### Simulation set-up for the SD simulations in vacuo without or with implicit-solvation forces

After energy minimisation, the protein in vacuo was slowly heated up using the same protocol as was used for the protein in water. After equilibration of 1 ns, the SD simulations in vacuo (SD_nowater, SD_implicit) were performed with a reference temperature of 308 K, maintained by the Langevin equations or thermostat and by weak coupling to a heat bath (*τ*_*Τ*_ = 0.1 ps), the latter in order to control the temperature of atoms that have a friction coefficient equal to zero, whose temperature is thus not controlled by the Langevin thermostat. Translational motion of the centre of mass of the system was removed every 2 ps. Trajectory energies and atomic coordinates were stored at 5 ps intervals and used for analysis (Eichenberger et al. 2011).

### MD and SD simulations performed

One MD simulation and eight SD simulations were performed, each 20 ns long:

1. MD_water: MD simulation of HEWL in a periodic box with 12,157 explicit water molecules and using the GROMOS 54A7 force field. The average solute and solvent temperatures were 311 K and 312 K, respectively.
2. SD_nowater: Four SD simulations of HEWL in vacuo, each with different initial velocities, using the GROMOS 54B7 force field. The average solute temperature was 309 K.
3. SD_implicit: Four SD simulations of HEWL in vacuo, each with different initial velocities, using the GROMOS 54B7 force field with the SASA implicit-solvation term and the set of solvation parameters of column 5 of Table 1 was used. The average solute temperature was 309 K.

### Analysis of atomic trajectories

The GROMOS force fields treat aliphatic carbons as united CH, CH_2_ and CH_3_ atoms. Therefore, when calculating NOE distances, inter-hydrogen distances involving the aliphatic hydrogen atoms were calculated using virtual atomic positions for CH and pro-chiral CH_2_ (van Gunsteren et al. 1985) and pseudo-atomic positions for CH_3_ (Wüthrich et al. 1983) for those hydrogen atoms (van Gunsteren et al. 2019b). The pseudo-atom NOE distance bound corrections of (Wüthrich et al. 1983) were used (van Gunsteren et al. 2016). The set of NOE distance upper bounds for HEWL (Smith et al. 1993; Schwalbe et al. 2001) can be found in Table S1 of Supporting Information, together with the values obtained from some simulations. The NOE between Trp 28 HZ3 and Leu 56 HG was reassigned as between Trp 28 HZ3 and Leu 56 HD* following reassessment of the experimental spectra. Inter-hydrogen distances were calculated as < *r*^−3^ > ^−1/3^, i.e. using *r*^−3^ averaging over the trajectory structures, where *r* indicates the actual hydrogen–hydrogen distance.

In view of the uncertainty inherent to the calculation of NOE bounds and *r*^−3^ averaged distances, deviations from experiment of less than 0.1 nm are considered insignificant.

Two sets of backbone ^*3*^*J*_*HN-Hα*_ couplings and two sets of side-chain ^*3*^*J*_*Hα-Hβ*_ couplings of HEWL (Smith et al. 2021a) were used, see Supporting Information Tables S2–S5.

1. A set (*bb1*) of 95 backbone ^*3*^*J*_*HN-Hα*_-coupling values, see Table II of (Smith et al. 1991) from which the values for 11 glycine residues were omitted, because these had not been stereo-specifically assigned.
2. A set (*bb2*) of 22 experimentally stereo-specifically unassigned backbone ^*3*^*J*_*HN-Hα*_-coupling values for the 11 glycine residues, see Table II of (Smith et al. 1991). 10 of these were stereo-specifically assigned (Smith et al. 2021a) based on a comparison of the ^*3*^*J*_*HN-Hα*_-coupling values calculated from MD simulations and from X-ray structures.
3. A set (*sc1*) of 58 ^*3*^*J*_*Hα-Hβ*_-coupling values, see Tables III and IV of (Smith et al. 1991), which were stereo-specifically assigned using experimental data.
4. A set (*sc2*) of 38 out of 40 experimentally stereo-specifically unassigned ^*3*^*J*_*Hα-Hβ*_-coupling values, see Table III of (Smith et al. 1991), which were stereo-specifically assigned (Smith et al. 2021a) based on the ^*3*^*J*_*Hα-Hβ*_-coupling values calculated from MD simulations. Only Glu 7 could not be stereo-specifically assigned.

**Fig. 2:**
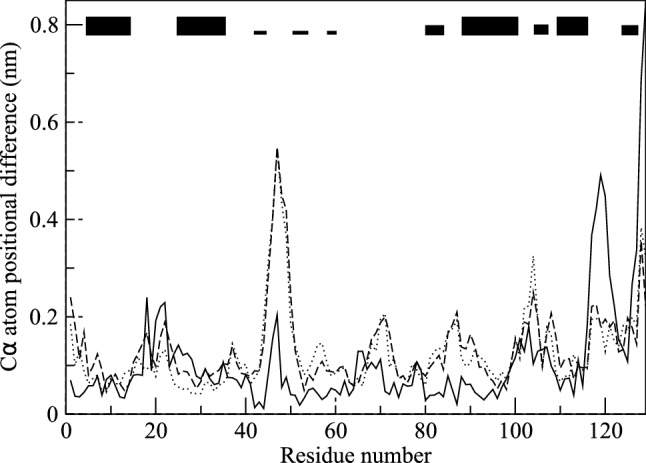
Backbone CA atom-positional root-mean-square differences (RMSD) between the *2VB1* X-ray structure and the final structures for the three types of simulations as function of residue sequence number. The structures are translationally and rotationally superimposed using the backbone atoms (N, CA, C) of residues 3–126. Solid line: MD_water simulation. Dotted line: SD_nowater simulations. Dashed line: SD_implicit simulations. The black bars at the top indicate secondary structure elements of HEWL (thick bars: α-helix; thinner bars: 3_10_-helix; narrow bars: ß-strand). The values for the SD simulations are averages over four simulations starting with different velocities

For the calculation of the backbone ^*3*^*J*_*HN-Hα*_-couplings, the Karplus relation (Karplus 1959, 1963) was used with the parameter values *a* = 6.4 Hz, *b* = -1.4 Hz and *c* = 1.9 Hz (Pardi et al. 1984), see Fig. 2 of (Smith et al. 2021a). The side-chain ^*3*^*J*_*Hα-Hβ*_-couplings were calculated using the parameter values *a* = 9.5 Hz, *b* = -1.6 Hz and *c* = 1.8 Hz (DeMarco et al. 1978), see Fig. 2 of (Smith et al. 2021a).

**Fig. 3:**
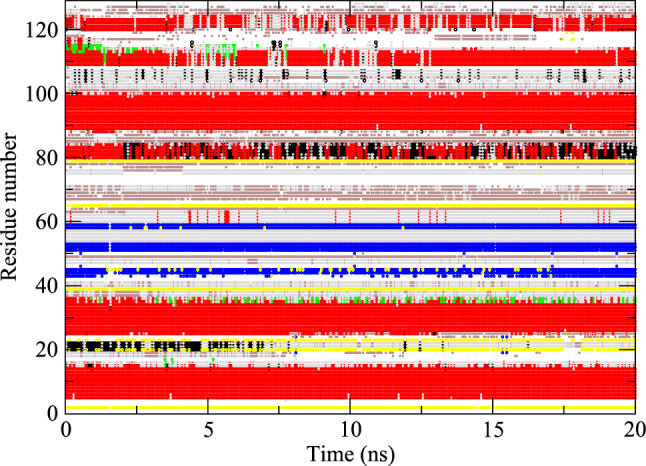
Secondary structure elements (Kabsch and Sander 1983) as a function of time calculated for the MD_water simulation. Red: α-helix; green: π-helix; black: 3_10_-helix; blue: ß-strand; yellow: ß-bridge; brown: bend; grey: turn

The experimentally derived ^*3*^*J*_*HN-Hα*_-coupling values for Val 2, Thr 51, Asp 66, Cys 115, Thr 118 and Ile 124 lie outside the Karplus curve, so were set to 9.7 Hz, which is the maximum of the Karplus curve used (Pardi et al. 1984). None of the experimentally derived ^*3*^*J*_*Hα-Hβ*_-coupling values lie outside the Karplus curve used (DeMarco et al. 1978). The nomenclature for the H_α2_ and H_α3_ atoms in Gly residues and the H_β_, H_β2_ and H_β3_ atoms in the side chains was defined as in Fig. 3 of (Markley et al. 1998). The values obtained from some simulations can be found in Tables S2–S5 of Supporting Information.

In view of the various factors contributing to an uncertainty of about 2 Hz inherent to the Karplus relation linking structure and ^*3*^* J*-couplings, a deviation of less than 2 Hz between ^*3*^* J*-coupling values calculated from MD trajectory structures and ^*3*^* J*-coupling values derived from experiment is considered insignificant.

Four sets of *S*^*2*^ order-parameter for HEWL, 121 for the backbone NH and 79 for the side-chain CH_3_, NH and NH_2_ moieties (Buck et al. 1995; Moorman et al. 2012), were used to evaluate the simulations, see Supporting Information Tables S6–S9. *S*^*2*^ order parameters for the atom pair (*a*,*b*) were calculated using the ensemble averaging expression (Henry and Szabo 1985)

22 $$S_{ab}^{2} = \frac{1}{2}\left\{ {3\sum\limits_{\alpha = 1}^{3} {\sum\limits_{\beta = 1}^{3} {\left\langle {\frac{{\mu_{ab\alpha } (t)\mu_{ab\beta } (t)}}{{r_{ab}^{3} (t)}}} \right\rangle_{t}^{2} } } - \left\langle {\frac{1}{{r_{ab}^{3} (t)}}} \right\rangle_{t}^{2} } \right\}(r_{ab}^{eff} )^{6} ,$$

where *t* indicates the time-averaging window, here 1 ns, shorter than the rotational correlation time of 5.7 ns of HEWL in solution (Smith et al. 1993),

23 $$\mu_{ab1} \equiv \, \left( {x_{a} {-}x_{b} } \right)/r_{ab} ,\, \mu_{ab2} \equiv \, \left( {y_{a} {-}y_{b} } \right)/r_{ab} ,\, \mu_{ab3} \equiv \, \left( {z_{a} {-}z_{b} } \right)/r_{ab} ,$$

are the *x*-, *y*-, and *z*-components of the vector $\vec{r}_{ab} \equiv \vec{r}_{a} - \vec{r}_{b}$ and $r_{ab} \equiv ((\vec{r}_{ab} )^{2} )^{1/2}$, its length (Hansen et al. 2014). To obtain a dimensionless quantity, the term in curly brackets is multiplied with the 6^th^ power of the effective length ($r_{ab}^{eff}$) of the vector $\vec{r}_{ab}$. Because in the present work, bond length constraints are used, the length of $\vec{r}_{ab}$ is essentially constant over time and its length thus equal to its effective value $r_{ab}^{eff}$.

Before calculating $S_{ab}^{2}$, the protein trajectory structures are superimposed using the backbone atoms (N, C_α_, C) of residues 3–126 in the fit in order to eliminate the effect of overall rotation of the protein upon the $S_{ab}^{2}$-values. Use of only the backbone atoms of four of the five α-helices and two β-strands in HEWL (residues 4–15, 24–36, 41–45, 50–53, 89–99, and 108–115) did not lead to significantly different $S_{ab}^{2}$-values.

For the Asn and Gln residues, only one *S*^*2*^_*NH*_*(exp)* value per NH_2_ group is available (Buck et al. 1995). This required the assignment to one of the two NH1 and NH2 bond vectors. This was done based on a comparison of the *S*^*2*^_*NH1*_*(sim)-* and *S*^*2*^_*NH2*_*(sim)*-values calculated from MD simulations (Smith et al. 2021b). The experimentally unassigned *S*^*2*^_*CG1*_- and *S*^*2*^_*CG2*_-values for Val and *S*^*2*^_*CD1*_- and *S*^*2*^_*CD2*_-values for Leu residues (Moorman et al. 2012) were assigned in a similar way (Smith et al. 2021b). The values obtained from some simulations can be found in Tables S6–S9 of Supporting Information.

In view of the uncertainty inherent to the derivation of *S*^*2*^_*ab*_*(exp)*-values from relaxation experiments and inherent to the calculation of *S*^*2*^_*ab*_*(sim)*-values from MD or SD simulations, a deviation of less than 0.2 between simulation and experiment is considered insignificant.

Atom-positional root-mean-square differences RMSD between trajectory structures and the *2VB1* X-ray crystal structure and atom-positional root-mean-square fluctuations (RMSF), i.e. around their average positions, in the MD and SD trajectories were calculated after superimposing the backbone atoms (N, CA, C) of residues 3 – 126 to eliminate the contribution of overall translation and rotation of the protein.

The radius of gyration *R*_*gyr*_ was calculated as

24 $$R_{gyr} \equiv \left( {\sum\limits_{i = 1}^{N} {(\vec{r}_{i} - } \vec{r}_{cm} )^{2} } \right)^{1/2} ,$$

with

25 $$\vec{r}_{cm} \equiv M^{ - 1} \sum\limits_{i = 1}^{N} {m_{i} \vec{r}_{i} }$$

and

26 $$M \equiv \sum\limits_{i = 1}^{N} {m_{i} } .$$

The secondary-structure assignment was done with the program DSSP, based on the Kabsch–Sander rules (Kabsch and Sander 1983).

Hydrogen bonds were identified according to a geometric criterion: a hydrogen bond was assumed to exist if the hydrogen-acceptor distance was smaller than 0.25 nm and the donor-hydrogen-acceptor angle was larger than 135°. The extent of hydrogen bonding was evaluated using the number of intra-solute hydrogen bonds in a simulation multiplied by their % occurrence in the simulation divided by the number of hydrogen bonds in the *2VB1* X-ray structure (Fraternali and van Gunsteren 1996).

The time evolution of structural features that would be sensitive to the way the solvent is modelled, was examined in terms of auto-correlation functions and spectral densities of atom positions and of torsional angles. From a time series of a quantity *Q(t)*, a normalised time correlation function,

27 $$C_{Q} (t) = \frac{{ < Q(\tau ) \cdot Q(\tau + t) >_{\tau } }}{{ < Q(\tau ) \cdot Q(\tau ) >_{\tau } }}$$

was calculated using the Fast Fourier Transform technique (Futrelle and McGinty 1971; van Gunsteren et al. 2019d). For these calculations, 25 ps towards the end of the simulations were repeated while saving configurations every 0.01 ps instead of 5 ps to obtain a finer resolution of the auto-correlation functions. When calculating the spectral density, only the first 2% of the auto-correlation function was used.

**Fig. 4:**
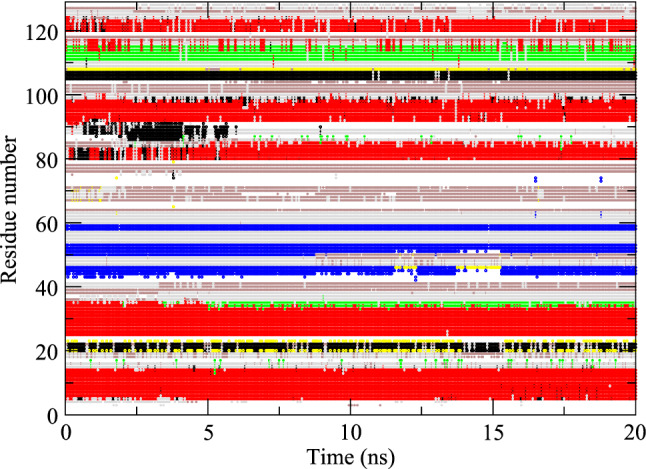
Secondary structure elements (Kabsch and Sander 1983) as a function of time calculated for an MD_nowater simulation. Red: α-helix; green: π-helix; black: 3_10_-helix; blue: ß-strand; yellow: ß-bridge; brown: bend; grey: turn

**Fig. 5:**
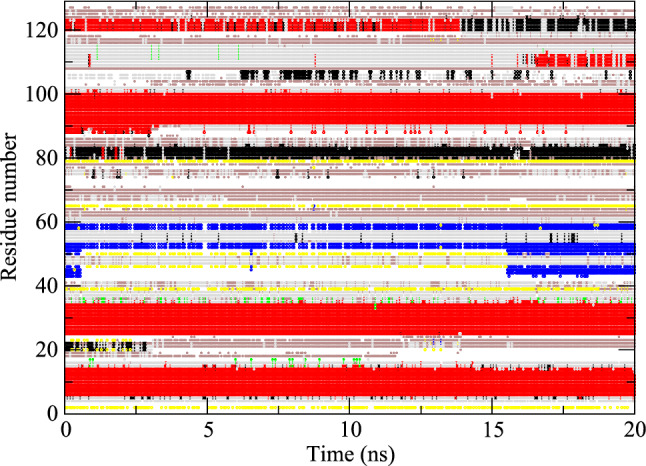
Secondary structure elements (Kabsch and Sander 1983) as a function of time calculated for an MD_implicit simulation. Red: α-helix; green: π-helix; black: 3_10_-helix; blue: ß-strand; yellow: ß-bridge; brown: bend; grey: turn

Although four SD_nowater and four SD_implicit simulations have been run, the data for only one simulation (those with RMSD and RMSF closest to the mean) of each type are shown in Figs. 4 and 5 and listed in the Supporting Information.

## Results and discussion

### Comparison of structural and energetic properties calculated from the simulations

In Table 2, average values of various properties of HEWL obtained from the three types of simulations, MD_water, SD_nowater and SD_implicit, are shown. As expected, both simulations without explicit water molecules, SD_nowater and SD_implicit, show larger atom-positional root-mean-square differences (RMSD) with the *2VB1* X-ray structure for the CA atoms (6% and 4%, respectively) and for all atoms (13% and 11%, respectively) than the protein solvated in explicit water (MD_water). Inclusion of an implicit-solvation force-field term in an in vacuo simulation does not improve the agreement with the *2VB1* X-ray crystal structure significantly. Figure 1 shows the *2VB1* X-ray crystal structure together with final structures of the three types of simulation. The final structure of the MD_water (red, Fig. 1b) simulation shows less deviation from the X-ray structure (grey) than the final structures of the simulations in vacuo, SD_nowater (green, Fig. 1c) and SD_implicit (blue, Fig. 1d). Figure 2 shows the atom-positional root-mean-square differences (RMSD) with the *2VB1* X-ray structure for the CA atoms as function of residue sequence number. The largest differences for both the SD_nowater and SD_implicit simulations is in an exposed turn between two ß-strands centred around Thr 47. There are also large differences in the loop region connecting helices C and D (particularly at Gly 104 for the *SD_nowater* simulation) and at the C-terminus. The *MD_water* simulation also shows a large difference at the C-terminus and changes around Asp 119, a region which contains a 3_10_ helix in the X-ray structure. The differences between explicit solvent on the one hand and no or implicit solvent on the other hand are larger than the differences between no solvent and implicit solvent.

**Table 2:** Averages of structural and energetic properties of HEWL calculated from the three types of simulations

Property	Unit	MD_water	SD_nowater	SD_implicit
RMSD(X-ray) CA	nm	0.292	0.309	0.304
RMSD(X-ray) all	nm	0.362	0.408	0.401
RMSF CA	nm	0.119	0.092	0.099
RMSF all	nm	0.161	0.135	0.142
Radius of gyration	nm	1.416	1.363	1.359
SASA hydrophylic	nm^2^	24.8	20.4	21.5
SASA hydrophobic	nm^2^	37.0	32.1	31.8
SASA total	nm^2^	82.8	69.2	70.3
Hydrogen bonds	%	96.0	120.5	117.2
Energy bonded (u:u)	kJ/mol	4355	4525	4511
Energy vdW (u:u)	kJ/mol	− 3399	− 3549	− 3533
Energy ele (u:u)	kJ/mol	− 10,777	− 10,056	− 10,036
Energy vdW (u:v)	kJ/mol	− 801	–	–
Energy ele (u:v)	kJ/mol	− 12,272	–	–
Energy SASA	kJ/mol	–	–	− 114

*RMSD* root-mean-square difference with the *2VB1* X-ray structure, *CA* CA atoms, *all* all atoms, *RMSF* root-mean-square fluctuation

Radius of gyration: see Eq. (24) (*2VB1* crystal structure: 1.405 nm). SASA: solvent-accessible-surface-area. Hydrogen bonds: number of intra-solute hydrogen bonds in a simulation multiplied by their % occurrence in the simulation divided by the number of hydrogen bonds in the *2VB1* X-ray structure (Fraternali and van Gunsteren 1996). Bonded (u:u): intra-solute bonded energy. vdW (u:u): intra-solute van der Waals energy. ele (u:u): intra-solute electrostatic energy. vdW (u:v): solute–solvent van der Waals energy. ele (u:v): solute–solvent electrostatic energy. SASA: implicit-solvation energy. The values for the SD simulations are averages over four simulations starting with different velocities

The lower radius of gyration in vacuo (SD_nowater: 96%, SD_implicit: 96%) compared to that in explicit water (MD_water) reflect the compaction of the protein in vacuo. The total solvent-accessible-surface-area is reduced by 16% in SD_nowater and by 15% in SD_implicit compared to the MD simulation in explicit water. For the hydrophilic area the numbers are 18% (SD_nowater) and 13% (SD_implicit). The reductions in hydrophobic area are 13% and 14% respectively.

The compaction also leads to about 26% (SD_nowater) and 22% (SD_implicit) more intra-protein hydrogen bonding in vacuo compared to explicit water, due to the missing hydrogen-bond donor and acceptor atoms of the water molecules absent in vacuo. The occurrence of secondary-structure elements (α-helix, π-helix, 3_10_-helix, ß-strand, ß-bridge, bend, turn) as function of time are shown for the three types of simulation in Figs. 3, 4 and 5. Compared to explicit water (Fig. 3), in vacuo without implicit-solvation term (Fig. 4) the fourth α-helix becomes shorter, a 3_10_-helix appears between the fourth and fifth α-helices, and the fifth changes into a wider π-helix. Using an implicit-solvation term in the force field (Fig. 5), the ß-sheet becomes less stable, the third α-helix turns in to a 3_10_-helix, and only later in the simulation the fifth α-helix appears while the sixth α-helix turns into a 3_10_-helix. In vacuo, the secondary-structure elements of HEWL become less stable than when simulated in explicit water.

The absence of many explicit water molecules in simulations in vacuo will influence the internal (u:u) energy of the protein (Table 2): In MD_water, it is -9821 kJ/mol, in SD_nowater it is higher, -9080 kJ/mol, and in SD_implicit, it is increased to − 9058 kJ/mol. Apparently, omission of explicit water molecules, bulk water, increases the internal energy of the protein, with the implicit-solvation force-field term more than without. The SASA energy of − 114 kJ/mol is a poor representation of the protein – explicit water energy of -12,272 kJ/mol, leading to slightly more strain in the molecule in vacuo than in explicit water. The reduced atom-positional root-mean-square fluctuations (RMSF, Table 2) in *SD_nowater* and *SD_implicit* compared to *MD_water* indicate a reduction of the internal entropy of the protein.

### Comparison of NOE distances, ^***3***^***J***-couplings and ***S***^***2***^ order parameters calculated from the simulations with experimentally derived values for HEWL

Table 3 shows the number of NOE distance upper bound violations in the *2VB1* X-ray crystal structure and for the three types of simulations of HEWL. The X-ray structure shows only 12 distance bound violations larger than 0.1 nm. MD simulation in explicit water leads to more, 42, of such violations, 2.6% of the total number of 1630 bounds. SD simulation in vacuo without an implicit-solvation force-field term leads to twice as many, 87 (5.3%) of such violations, in particular large (> 0.3 nm) ones, and the introduction of the implicit-solvation force-field term yields less of such violations, 65 (4.0%). The use of an implicit-solvation force-field term improves the agreement with the NOE data, but the agreement is still worse than when using explicit solvation in the MD simulation.

**Table 3:** Number of NOE distance bound violations in the *2VB1* X-ray crystal structure and in the three types of simulations of HEWL

Structure or simulation	Size of NOE distance bound violation (in nm)					
	0.05 – 0.1	0.1 – 0.15	0.15 – 0.2	0.2 – 0.25	0.25 – 0.3	> 0.3
X-ray_2VB1	21	7	5	0	0	0
MD_water	44	18	11	5	3	5
SD_nowater	47	31	18	9	8	21
SD_implicit	48	26	15	6	9	9

Number of NOE distance bounds: 1630. The values for the SD simulations are averages over four simulations starting with different velocities

Table 4 shows the number of deviations from experimentally derived values for different types of ^*3*^* J*-couplings in the *2VB1* X-ray crystal structure and for the three types of simulations of HEWL. The numbers of available measured ^*3*^* J*-couplings are for the backbone 95 NMR-assigned and 22 MD/X-ray-assigned ^*3*^* J*-couplings and for the side chains 58 NMR-assigned and 38 MD-assigned ^*3*^* J*-couplings, in total 213 ^*3*^* J*-coupling values (Smith et al. 2021a). The X-ray structure shows 3 (2.6%) backbone, 15 (26%) NMR-assigned side-chain and 25 (66%) MD-assigned side-chain ^*3*^* J*-coupling deviations larger than 2 Hz. In the MD simulation of HEWL in explicit water these values are 19 (16%), 14 (24%) and 13 (34%), respectively. SD simulation in vacuo without implicit-solvation force-field term leads to larger deviations, 25.7 (27%) NMR-assigned backbone and 2.2 (10%) MD/X-ray-assigned backbone ^*3*^* J*-couplings, and 20.0 (34%) NMR-assigned side-chain and 18.9 (50%) MD-assigned side-chain ^*3*^* J*-coupling deviations larger than 2 Hz. The introduction of an implicit-solvation force-field term does not change these values significantly, with 24.8 (26%) NMR-assigned and 2.9 (13%) MD/X-ray-assigned backbone ^*3*^* J*-couplings, and 23.2 (40%) NMR-assigned side-chain and 18.0 (47%) MD-assigned side-chain ^*3*^* J*-coupling deviations larger than 2 Hz. The large deviations for the *SD_nowater* and *SD_implicit* simulations are particularly for residues in the long loop region (especially residues 61–74) and residues 45–51 in the exposed turn between two ß-strands where the final simulation structures show a large CA atom-positional RMSD to the *2VB1* X-ray structure (Fig. 2). MD simulation in explicit water yields, compared to the X-ray crystal data, worse agreement for the backbone ^*3*^* J*-couplings, but slightly better agreement for the side-chain ^*3*^* J*-couplings. The SD simulations in vacuo, without or with implicit-solvation force-field term, show significantly worse agreement with the experimentally derived ^*3*^* J*-coupling data.

**Table 4:** Number of deviations, |^*3*^* J* (exp)– ^*3*^* J* (MD, SD or X-ray)|, in the *2VB1* X-ray structure and in the three types of simulations of HEWL, for the 95 and 22 backbone (bb) ^*3*^*J*_*HNHα*_-coupling values derived from NMR measurements and assigned based on the NMR data or stereo-specifically on MD simulation or X-ray data, and for the 58 and 38 side-chain (sc) ^*3*^*J*_*HαHβ*_-coupling values derived from NMR measurements and stereo-specifically assigned based on NMR measurements or on MD simulation data (Smith et al. 2021a)

Type of ^*3*^* J*-coupling, assignment (number)	Crystal structure or simulation	Size of ^*3*^*J*_*HNHα*_ or ^*3*^*J*_*HαHβ*_ deviation (in Hz)				
		1 – 2	2 – 3	3 – 4	4 – 5	> 5
bb: ^*3*^*J*_*HNHα*_ assigned NMR (95)	X-ray	13	2	1	0	0
	MD_water	25	10	8	0	0
	SD_nowater	21.5	12.8	7.2	4.2	1.5
	SD_implicit	23.2	13.2	6.8	3.8	1.0
bb: ^*3*^*J*_*HNHα*_ assigned MD/X-ray (22)	X-ray	5	0	0	0	0
	MD_water	3	1	0	0	0
	SD_nowater	5.8	1.2	0.8	0.2	0.0
	SD_implicit	4.5	1.2	1.2	0.5	0.0
sc: ^*3*^*J*_*HαHβ*_ assigned NMR (58)	X-ray	23	9	4	1	1
	MD_water	18	2	5	4	3
	SD_nowater	16.2	8.0	1.8	2.2	8.0
	SD_implicit	16.0	8.2	2.0	2.8	10.2
sc: ^*3*^*J*_*HαHβ*_ assigned MD (38)	X-ray	9	3	4	5	13
	MD_water	4	9	2	0	2
	SD_nowater	8.8	5.2	7.5	3.0	3.2
	SD_implicit	9.5	5.2	5.8	4.5	2.5

The values for the SD simulations are averages over four simulations starting with different velocities

Table 5 shows the number of deviations from experimentally derived values for different types of *S*^*2*^ order parameters for the three types of simulations of HEWL. The numbers of available experimentally derived *S*^*2*^ order-parameter values are 121 backbone *S*^*2*^_*NH*_-values, 79 side-chain *S*^*2*^-values, that is, 51 *S*^*2*^_*CH*_-values of Ala, Ile, Leu, Met, Thr and Val residues, 11 *S*^*2*^_*NH*_-values of Trp and Arg residues and 17 *S*^*2*^_*NH2*_-values of Asn and Gln residues, in total 200 *S*^*2*^ order-parameter values (Smith et al. 2021b). The MD simulation of HEWL in explicit water shows for the backbone *S*^*2*^_*NH*_ order parameters 21 (17%) deviations larger than 0.2 and for the side-chain *S*^*2*^ order parameters 25 (55%) deviations larger than 0.2, that is, 21 (41%) *S*^*2*^_*CH*_-values of Ala, Ile, Leu, Met, Thr and Val residues, 1 (9%) *S*^*2*^_*NH*_-value of a Trp residue and 3 (18%) *S*^*2*^_*NH2*_-values of Asn and Gln residues. SD simulations in vacuo yield better agreement with the experimentally derived values for the backbone, but worse agreement for the side chains. Without implicit-solvation force-field term, the deviations larger than 0.2 are 10.0 (8%) for the backbone and 18.4 (36%), 1.7 (15%) and 8.1 (48%) for the three types of side-chain *S*^*2*^ order parameters, respectively. Inclusion of an implicit-solvation force-field term does not change the agreement significantly, with deviations larger than 0.2 of 10.2 (8%) for the backbone and 19.2 (38%), 2.2 (20%) and 8.2 (48%) for the three types of side-chain *S*^*2*^ order parameters, respectively. The significant increase in the deviations of the *S*^*2*^_*NH2*_-values of Asn and Gln residues for *the SD_nowater and SD_implicit simulations comes from* residues with higher calculated *S*^2^ values from the simulations than those observed experimentally. These Asn and Gln side chains form persistent intra-protein hydrogen bonds in the SD_nowater and SD_implicit simulations, while in the X-ray structure and MD_water simulation they hydrogen bond to crystallographic waters and form short-lived hydrogen bonds to bulk water molecules, respectively. For example, the side chain of Asn 19 hydrogen bonds to the backbone carbonyl group of Asp 18 with populations of 65% and 33% in the SD_nowater and SD_implicit simulations, respectively, and the side chain of Gln 121 hydrogen bonds to the backbone carbonyl group of Thr 118 in the SD_nowater simulation (population 66%) and to the backbone carbonyl group of Arg 128 in the SD_implicit simulation (population 57%). None of these hydrogen bonds are present in the X-ray structure or the MD_water simulation (Asn 19: Experimental S^2^ 0.43, MD_water 0.49, SD_nowater 0.77, SD_implicit 0.76; Gln 121: Experimental S^2^ 0.36, MD_water 0.34, SD_nowater 0.78, SD_implicit 0.63). Overall, as for the ^3^ *J*-couplings, the SD simulations in vacuo, without or with implicit-solvation force-field term, show overall worse agreement with the experimentally derived *S*^*2*^ order-parameter values.

**Table 5:** Number of deviations, |*S*^*2*^(exp)—*S*^*2*^(MD or SD)|, for the 121 backbone *S*^*2*^_*NH*_-values and for the 79 side-chain *S*^*2*^-values, that is, 51 *S*^*2*^_*CH*_-values, 11 *S*^*2*^_*NH*_-values of Trp and Arg residues and 17 *S*^*2*^_*NH2*_-values of Asn and Gln residues (Smith et al. 2021b), in the three types of simulations of HEWL

	Simulation	Size of *S*^*2*^ deviation					
		0.05–0.1	0.1 – 0.2	0.2 – 0.3	0.3 – 0.4	0.4 – 0.5	> 0.5
Backbone *S*^*2*^_*NH*_ (121)	MD_water	27	27	13	6	2	0
	SD_nowater	37.0	25.8	8.0	1.2	0.8	0.0
	SD_implicit	35.8	28.9	8.0	2.0	0.2	0.0
Side-chain *S*^*2*^_*CH*_ (51)	MD_water	7	12	9	6	5	1
	SD_nowater	9.5	11.0	6.8	5.2	3.2	3.2
	SD_implicit	10.2	9.0	8.8	2.8	5.8	1.8
Side-chain *S*^*2*^_*NH*_ Trp/Arg (11)	MD_water	2	3	0	1	0	0
	SD_nowater	4.0	1.8	0.5	1.2	0.0	0.0
	SD_implicit	3.5	1.2	1.5	0.5	0.0	0.2
Side-chain *S*^*2*^_*NH2*_ Asn/Gln (17)	MD_water	6	5	2	1	0	0
	SD_nowater	1.5	4.8	1.8	2.8	2.5	1.0
	SD_implicit	2.8	5.2	3.8	2.8	0.8	0.8

The values for the SD simulations are averages over four simulations starting with different velocities

An early comparison with experimental data of various properties of HEWL as obtained by MD simulation in vacuo and in water (Smith et al. 1995) used the older GROMOS force-field versions 37C4 (MD in water) and 37D4 (MD in vacuo) and also a modified version of 37C4 (MD in water) with explicit inclusion of aromatic hydrogens and a modified interaction between water oxygen and the carbon atoms of the protein. These force-field versions were not yet calibrated to free-energy (energy and entropy) data of various compounds in solution. The experimental data compared to were 1158 NOE atom–atom distance upper bounds, 163 ^*3*^* J*-couplings and 159 S^2^ order-parameter values, 29% (NOEs), 23% (^*3*^* J*-couplings), and 20% (*S*^*2*^ order parameters) less data than in the current study (1630 NOEs, 41% more; 213 ^*3*^* J*-couplings, 31% more; 200 *S*^*2*^ order parameters, 26% more). The currently used force field (54A7) and X-ray crystal structure (*2VB1*) yield better agreement with the larger experimental data set than the older force field (37C4, 37C4 modified) and older X-ray crystal structure (*2LZT*) with the smaller experimental data set. For the X-ray structures, there are 21 (current) vs 15 (in 1995) distance upper bound violations in the range 0.05–0.1 nm, 12 (current) vs 25 (in 1995) violations in the range 0.1–0.3 nm, and 0 (current) vs 2 (in 1995) violations in the range > 0.3 nm. In the simulation of HEWL in water, the current force field (54A7) yields better agreement with the larger experimental data set than the older force fields (37C4, with or without modifications) with the smaller experimental data set. For the water MD simulations there are 44 (current) vs 40 and 31 (in 1995) distance bound violations in the range 0.05 – 0.1 nm, 37 (current) vs 47 and 64 (in 1995) violations in the range 0.1 – 0.3 nm, and 5 (current) vs 7 and 17 (in 1995) violations in the range > 0.3 nm. The current SD simulation in vacuo shows an agreement with the larger experimental data set that is comparable to that of the MD simulation in vacuo using the older force field (37D4) and the smaller experimental data set. For the SD and MD simulations in vacuo there are 47 (current) vs 41 (in 1995) distance bound violations in the range 0.05 – 0.1 nm, 66 (current) vs 61 (in 1995) violations in the range 0.1 – 0.3 nm, and 21 (current) vs 20 (in 1995) violations in the range > 0.3 nm.

### Comparison of dynamical properties calculated from the simulations

**Fig. 6:**
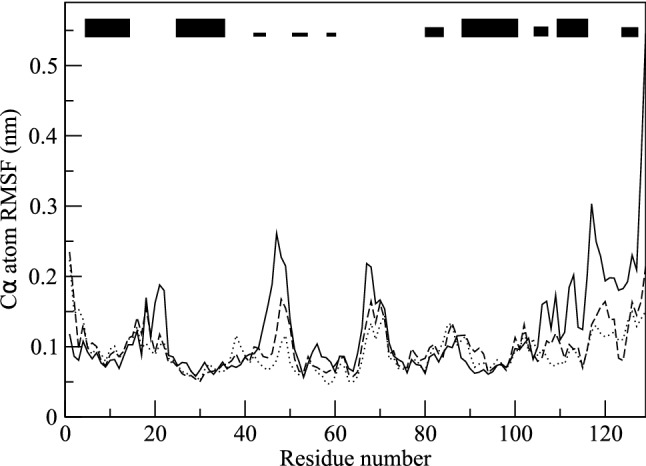
Backbone CA atom-positional root-mean-square fluctuations (RMSF) as function of residue sequence number for the three types of simulations MD_water (solid line), MD_nowater (dotted line) and MD_implicit (dashed line). The trajectory structures are translationally and rotationally superimposed using the backbone atoms (N, CA, C) of residues 3–126. The black bars at the top indicate secondary structure elements of HEWL (thick bars: α-helix; thinner bars: 3_10_-helix: narrow bars: ß-strand). The values for the SD simulations are averages over four simulations starting with different velocities

Table 2 shows the atom-positional root-mean-square fluctuations (RMSF) in the three types of simulations, MD_water, SD_nowater and SD_implicit, as averages over the backbone CA atoms and as averages over all atoms. The backbone CA atom-positional root-mean-square fluctuations as function of residue sequence number for the three types of simulations are shown in Fig. 6. As expected, both simulations without explicit water molecules (SD_nowater: dotted line, SD_implicit: dashed line) show less mobility of the atoms than the protein solvated in explicit water (MD_water: black line). This due to the vacuo boundary condition applied in the former simulations, which leads to a compaction of the protein and thus less mobility. The use of the implicit–solvation force-field term leads to somewhat more mobility than without such a term, but the mobility is still only 83% (CA atoms) and 88% (all atoms) of that in explicit water. Without implicit–solvation force-field term these values are 77% and 84%, respectively.

**Fig. 7:**
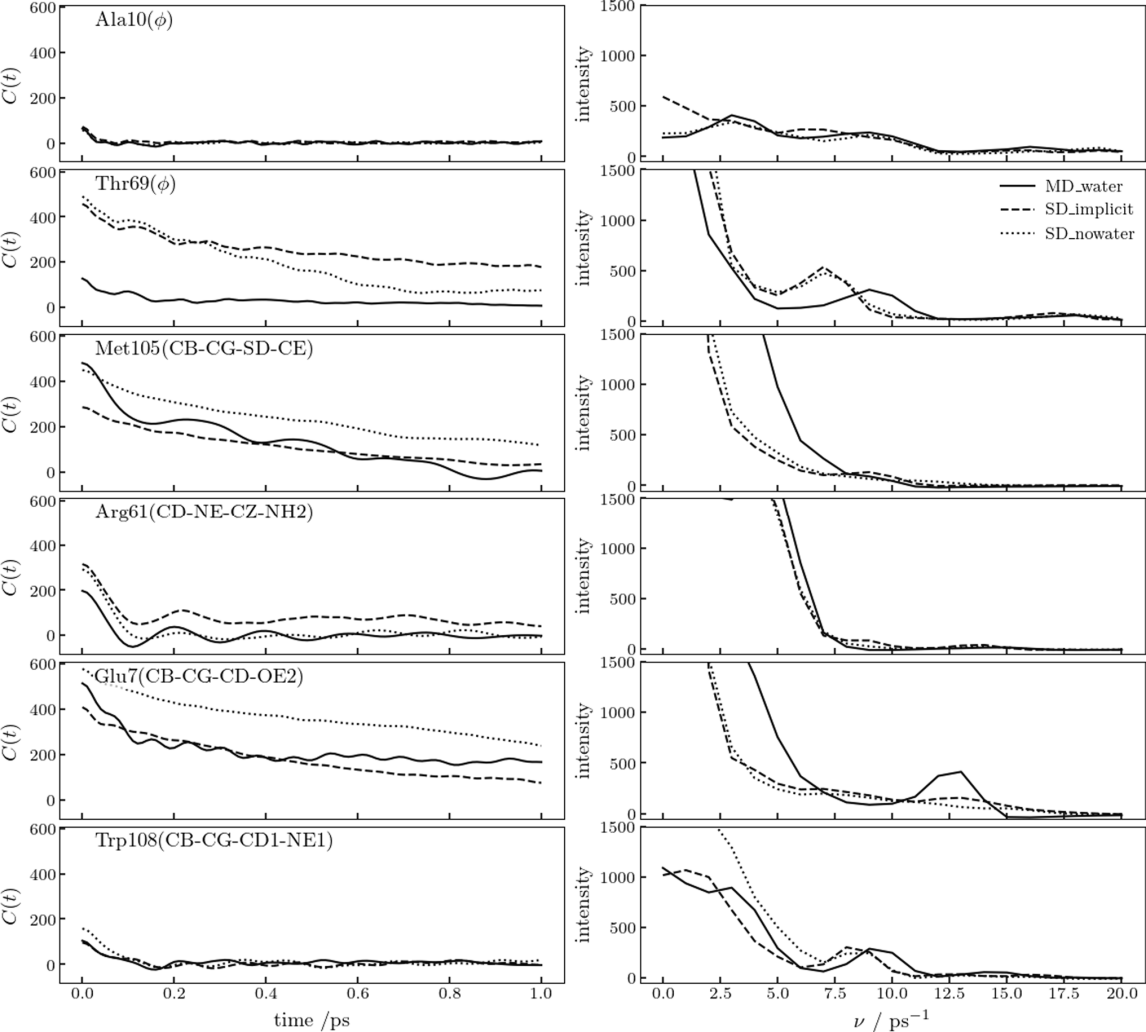
Auto-correlation function (left panels) and spectral density (right panels) of six torsional angles in HEWL in the three types of simulations. From top to bottom: *φ*(Ala 10), *φ*(Thr 69), *χ*_*3*_(CB-CG-SD-CE; Met 105), *χ*_*5*_(CD-NE-CZ-NH2; Arg 61), *χ*_*3*_(CB-CG-CD-OE2; Glu 7), *χ*_*3*_(CB-CG-CD1-NE1; Trp 108). Solid lines: *MD_water* simulation. Dotted lines: *SD_nowater* simulation. Dashed lines: *SD_implicit* simulation. Configurations from 25 ps towards the end of the simulations, separated by 0.01 ps were used to calculate the auto-correlation functions and only the first 2% of the auto-correlation function was used to calculate the spectral density

A more detailed picture of the differences in dynamics of the protein atoms in the different types of simulations can be obtained by calculating auto-correlation functions for various degrees of freedom in the protein. In Fig. 7, the auto-correlation functions and spectral densities of six torsional angles are shown: the backbone *φ*-angles of Ala 10 (in an α-helix) and of Thr 69 (in the long loop in the ß-domain), the side-chain *χ*_*3*_-angle of Met 105 (in the so-called hydrophobic box of HEWL), the side-chain *χ*_*5*_-angle of Arg 61 (at the end of the ß-sheet, which shows a much higher mobility than its backbone angles), the side-chain *χ*_*3*_-angle of Glu 7 (which is mobile despite being part of an α-helix), and the side-chain *χ*_*3*_-angle of Trp 108 (in the hydrophobic box). The auto-correlation function of the backbone *φ*-angle of Ala 10 in an α-helix is flat and almost identical for all three types of simulations, which is not surprising. The backbone *φ*-angle of Thr 69 in the long loop shows more long-time correlation, most in SD_implicit and least in MD_water. The peak in the spectral density between 5 and 12 ps^−1^ occurs in explicit solvent at a slightly higher frequency (9 ps^−1^) than in vacuo (7 ps^−1^). The *χ*_*3*_-angles in the side chains of Met 105 and Glu 7 display similar behaviour. The auto-correlations in MD_water and MD_implicit decay faster than in MD_nowater. The small oscillations in the auto-correlation function of Glu 7 in MD_water lead to a peak at 13 ps^−1^ in the spectral density. For the *χ*_*5*_-angle of Arg 61, the auto-correlation functions and spectral densities in the three types of simulations are rather similar, and the same observation holds, to a lesser extent, for the *χ*_*3*_-angle of Trp 108. Overall, the short-time dynamics does not differ greatly between the different types of simulations, while the difference between explicit solvent and no or implicit solvent is somewhat larger than between no solvent and implicit solvent.

## Conclusions

Generally, structure refinement of proteins in crystal or in (aqueous) solution is carried out for the solute molecule in vacuo, that is, without treating the solvent (water) degrees of freedom explicitly. Omission of solvent molecules may, however, lead to distortions in the protein structure, dynamics, internal energy and entropy. This has been investigated for the protein hen egg white lysozyme (HEWL), for which ample experimentally derived data are available, which may be used to evaluate the extent of the mentioned distortions.

Omission of bulk water in a simulation leads to a compaction of the protein, a lower radius of gyration and solvent-accessible-surface-area, an increase of protein-internal hydrogen bonding, an increase of the protein-internal energy and strain due to missing interactions with water molecules, and a reduction of the protein-internal entropy. A comparison with various experimentally derived data show a higher number of NOE distance upper bound violations: in explicit water 2.6% of the 1630 bounds, in SD_nowater 5.3% and in SD_implicit 4.0%. The experimentally derived 213 ^*3*^* J*-couplings and 200 *S*^*2*^ order parameters are much less well reproduced by simulation in vacuo, without or with implicit-solvation term, than by simulating the motion of the protein degrees of freedom and explicitly those of bulk water solvating the protein.

The rather large differences found between simulating a protein in explicit water on the one hand and simulating it in vacuo on the other hand can be understood from the particular properties of water: the rather large entropy content of bulk water at ambient temperature and pressure, the hydrogen-bonding capacity of individual water molecules and the dielectric screening of protein-internal electrostatic interactions by high-permittivity bulk water. These three features also explain why the addition of an implicit-solvation mean-force term to the force field applied does not help much to off-set the omission of explicit water molecules. The three mentioned fundamental flaws are inherent to any implicit-solvation model.

The results for HEWL presented here constitute only one example of the deficits of protein simulation or refinement models that ignore the influence of solvent (water) upon the protein properties in (aqueous) solution. HEWL is a challenging case regarding in vacuo simulation: it is a non-spherical, not compact protein with an overall charge of + 10e, containing a variety of secondary-structure elements and loops. This suggests that for relatively spherical, compact proteins, for example ubiquitin, the effects of omission of water molecules in simulation or refinement may be less pronounced. However, as the detailed comparisons with experimental data presented here show, even if significant overall changes to the structure are not observed, the torsion angles of residues in exposed turns and loops and the dynamical behaviour of exposed side chains may not be correctly represented. As these groups are often involved in protein–protein interactions or ligand or substrate binding, the correct modelling of their properties is of particular importance.

Therefore, in structure refinement of proteins in aqueous solution based on a limited set of experimentally derived data, as compared to the number of protein-internal degrees of freedom, the use of explicit water molecules is essential, for HEWL see e.g. (Smith et al. 2021a, b). In view of the abundance of X-ray reflections for proteins in crystalline form, the use of explicit water molecules in structure refinement based on X-ray data is less essential, but will enhance the physical reliability of the resulting structures, for bovine pancreatic trypsin inhibitor (BPTI) see e.g. (Gros et al. 1990; Schiffer and van Gunsteren 1999).

## Supplementary Information

Below is the link to the electronic supplementary material.

Supplementary file1 (DOCX 335 kb)

Supplementary file2 (DOCX 18 kb)

Supplementary file3 (DOCX 14 kb)

Supplementary file4 (DOCX 17 kb)

Supplementary file5 (DOCX 16 kb)

Supplementary file6 (DOCX 18 kb)

Supplementary file7 (DOCX 20 kb)

Supplementary file8 (DOCX 14 kb)

Supplementary file9 (DOCX 16 kb)
