# Supplementary material for: Molecular dynamics simulation or structure refinement of proteins: are solvent molecules required? A case study using hen lysozyme
Source: Eur Biophys J. 2022 Mar 18;51(3):265–82. doi: 10.1007/s00249-022-01593-1 (PMC9035012; doi:10.1007/s00249-022-01593-1)
Supplement: Supplementary file 1 — Supplementary file1 (DOCX 335 kb) [file 249_2022_1593_MOESM1_ESM.docx]

Table S2. Backbone *^3^J_HNHα_*-coupling values (95) in Hz derived and assigned based on NMR measurements and calculated from the *2VB1* X-ray structure, the MD simulation in explicit water using the GROMOS 54A7 force field (*MD_water*), the SD simulations in vacuo using the GROMOS 54B7 force field without (*SD_nowater*) and with (*SD_implicit*) a SASA implicit-solvation term. Experimental values from Table II of (Smith et al. 1991). The value within brackets in the column “Experimental value” represents the maximum in the Karplus relation (Pardi et al. 1984) used for the calculation of the *^3^J_HNHα_*-couplings. The root-mean-square fluctuations (RMSF) of the *^3^J*-couplings in the simulations are given within parentheses.

| Residue | Experimental value | *X-ray structure 2VB1* | *MD_water* | *SD_nowater* | *SD_implicit* |
| --- | --- | --- | --- | --- | --- |
| Val 2 | 10.0 (9.7) | 9.6 | 7.9 (1.6) | 7.5 (1.7) | 7.6 (1.6) |
| Phe 3 | 7.4 | 6.1 | 7.5 (1.7) | 6.4 (1.3) | 6.6 (1.4) |
| Cys 6 | 5.8 | 5.8 | 5.5 (1.1) | 4.5 (1.1) | 4.5 (1.0) |
| Glu 7 | 4.5 | 4.6 | 4.3 (1.0) | 4.7 (1.0) | 4.9 (1.0) |
| Leu 8 | 5.5 | 5.1 | 4.7 (1.0) | 4.8 (1.1) | 4.7 (1.0) |
| Ala 9 | 3.7 | 3.7 | 4.2 (0.9) | 4.2 (0.9) | 4.1 (0.9) |
| Ala 10 | 3.9 | 4.8 | 4.5 (0.9) | 4.5 (1.0) | 4.6 (0.9) |
| Ala 11 | 4.8 | 4.7 | 4.5 (0.9) | 4.6 (1.0) | 4.6 (1.0) |
| Met 12 | 4.6 | 4.5 | 4.9 (1.0) | 5.0 (1.1) | 4.8 (1.0) |
| Lys 13 | 4.2 | 4.8 | 4.7 (1.0) | 4.4 (0.9) | 4.3 (1.0) |
| Arg 14 | 4.4 | 4.1 | 4.6 (1.0) | 4.4 (1.0) | 4.2 (1.0) |
| His 15 | 9.2 | 7.6 | 7.4 (1.6) | 7.7 (1.5) | 6.9 (1.4) |
| Leu 17 | 7.6 | 7.5 | 7.0 (1.7) | 5.9 (1.4) | 6.7 (1.4) |
| Asp 18 | 5.7 | 5.3 | 6.0 (1.9) | 5.0 (1.4) | 5.2 (1.5) |
| Asn 19 | 7.0 | 6.6 | 6.4 (1.4) | 5.4 (1.3) | 5.3 (1.4) |
| Tyr 20 | 5.5 | 5.0 | 7.0 (2.3) | 8.3 (1.4) | 6.9 (1.7) |
| Arg 21 | 6.8 | 6.7 | 6.0 (1.3) | 4.5 (1.2) | 5.3 (1.3) |
| Tyr 23 | 8.6 | 9.5 | 7.8 (1.9) | 6.4 (1.4) | 7.3 (1.7) |
| Asn 27 | 5.4 | 4.0 | 4.2 (1.0) | 5.1 (1.0) | 5.1 (1.0) |
| Trp 28 | 6.0 | 5.2 | 5.2 (1.0) | 4.6 (1.0) | 5.3 (1.0) |
| Val 29 | 5.9 | 4.9 | 4.8 (0.9) | 4.7 (0.9) | 4.4 (0.9) |
| Cys 30 | 3.8 | 4.0 | 4.6 (0.9) | 4.6 (0.9) | 3.9 (0.8) |
| Ala 31 | 3.8 | 4.2 | 4.4 (0.9) | 4.6 (0.9) | 4.6 (1.0) |
| Ala 32 | 4.8 | 4.7 | 4.5 (0.9) | 4.6 (1.0) | 4.6 (0.9) |
| Lys 33 | 3.6 | 3.6 | 4.6 (1.0) | 4.7 (1.2) | 4.6 (1.1) |
| Phe 34 | 7.6 | 7.7 | 5.6 (1.4) | 6.1 (1.3) | 6.0 (1.3) |
| Glu 35 | 7.2 | 5.6 | 7.7 (1.5) | 8.7 (1.1) | 7.6 (1.4) |
| Ser 36 | 9.6 | 9.4 | 7.9 (1.7) | 7.7 (1.4) | 6.9 (1.6) |
| Phe 38 | 6.3 | 6.8 | 6.8 (0.2) | 6.7 (0.3) | 6.8 (0.2) |
| Asn 39 | 8.8 | 8.6 | 8.1 (1.5) | 7.3 (1.6) | 7.4 (1.8) |
| Thr 40 | 5.4 | 4.7 | 4.7 (1.1) | 4.3 (1.0) | 4.4 (1.1) |
| Gln 41 | 9.2 | 8.7 | 6.5 (1.5) | 5.1 (1.3) | 7.9 (1.4) |
| Ala 42 | 4.5 | 4.0 | 5.6 (1.6) | 5.5 (1.3) | 5.3 (1.6) |
| Thr 43 | 9.3 | 9.2 | 8.7 (1.3) | 5.3 (1.2) | 6.1 (1.8) |
| Asn 44 | 9.4 | 9.2 | 7.7 (1.7) | 5.8 (1.6) | 6.1 (1.9) |
| Arg 45 | 7.7 | 8.4 | 8.1 (1.4) | 7.0 (1.7) | 6.0 (2.1) |
| Asn 46 | 8.8 | 7.5 | 6.1 (1.6) | 8.9 (1.0) | 7.2 (1.9) |
| Thr 47 | 4.4 | 6.2 | 4.2 (1.0) | 4.1 (1.1) | 4.2 (1.3) |
| Asp 48 | 7.7 | 6.8 | 6.3 (1.4) | 6.0 (1.3) | 6.2 (1.4) |
| Ser 50 | 7.8 | 8.0 | 5.3 (1.4) | 6.7 (1.9) | 7.1 (2.2) |
| Thr 51 | 9.8 (9.7) | 9.0 | 8.3 (1.5) | 6.8 (1.8) | 6.5 (1.5) |
| Asp 52 | 9.6 | 9.0 | 8.0 (1.3) | 7.8 (1.5) | 7.0 (1.9) |
| Tyr 53 | 9.6 | 9.7 | 8.9 (1.0) | 8.8 (1.1) | 8.9 (1.1) |
| Leu 56 | 9.7 | 9.5 | 7.3 (1.2) | 8.5 (1.3) | 7.3 (1.4) |
| Gln 57 | 6.3 | 6.7 | 6.8 (0.2) | 5.1 (1.4) | 6.7 (0.3) |
| Ile 58 | 8.0 | 7.2 | 8.0 (1.2) | 6.4 (1.5) | 5.2 (1.2) |
| Ser 60 | 5.1 | 7.1 | 5.2 (1.1) | 6.0 (1.5) | 4.8 (1.1) |
| Arg 61 | 6.2 | 6.7 | 5.7 (1.5) | 6.1 (1.6) | 5.4 (1.2) |
| Cys 64 | 8.8 | 9.4 | 7.5 (1.5) | 9.2 (0.7) | 8.8 (1.2) |
| Asn 65 | 9.4 | 8.8 | 6.3 (1.3) | 6.1 (1.3) | 6.9 (1.4) |
| Asp 66 | 10.0 (9.7) | 9.6 | 8.1 (1.6) | 9.1 (0.8) | 7.3 (1.7) |
| Arg 68 | 9.7 | 9.6 | 6.9 (1.9) | 5.2 (1.5) | 4.6 (1.4) |
| Thr 69 | 9.3 | 9.5 | 6.0 (1.9) | 6.7 (1.9) | 8.1 (1.6) |
| Cys 76 | 8.8 | 7.2 | 7.4 (1.5) | 6.4 (1.3) | 5.8 (1.3) |
| Asn 77 | 7.4 | 6.9 | 6.8 (0.2) | 6.7 (0.2) | 6.7 (0.2) |
| Ile 78 | 8.0 | 8.3 | 4.7 (1.3) | 6.1 (1.3) | 4.4 (1.1) |
| Cys 80 | 3.6 | 4.5 | 4.5 (0.9) | 4.5 (0.9) | 4.7 (1.2) |
| Ser 81 | 3.6 | 4.1 | 4.0 (0.9) | 4.2 (0.9) | 4.1 (1.0) |
| Ala 82 | 5.4 | 4.7 | 4.7 (1.0) | 5.3 (1.2) | 4.3 (1.1) |
| Leu 83 | 7.2 | 6.4 | 5.3 (1.2) | 5.2 (1.2) | 5.1 (1.1) |
| Leu 84 | 9.2 | 9.4 | 5.6 (1.3) | 4.5 (1.1) | 5.9 (1.1) |
| Ser 85 | 5.8 | 5.2 | 6.3 (1.9) | 6.5 (1.6) | 5.7 (1.8) |
| Ser 86 | 5.8 | 5.8 | 4.9 (1.2) | 5.7 (1.1) | 5.4 (1.2) |
| Asp 87 | 8.9 | 6.4 | 6.5 (1.9) | 6.3 (1.2) | 6.4 (1.2) |
| Ile 88 | 6.5 | 8.0 | 7.3 (1.8) | 3.5 (1.2) | 5.4 (1.3) |
| Ala 90 | 4.2 | 5.2 | 4.5 (1.0) | 4.3 (1.0) | 6.2 (1.1) |
| Ser 91 | 5.5 | 4.7 | 4.2 (1.0) | 4.9 (1.0) | 4.8 (1.0) |
| Val 92 | 5.6 | 4.8 | 4.8 (1.0) | 5.0 (1.0) | 4.9 (1.0) |
| Asn 93 | 4.4 | 4.7 | 4.5 (0.9) | 4.4 (0.9) | 4.9 (1.0) |
| Cys 94 | 6.3 | 5.5 | 5.1 (1.0) | 5.1 (1.0) | 4.6 (0.9) |
| Lys 96 | 4.4 | 4.3 | 4.3 (0.9) | 4.1 (0.9) | 4.4 (1.0) |
| Lys 97 | 6.5 | 4.5 | 4.7 (1.0) | 5.5 (1.4) | 4.7 (1.0) |
| Val 99 | 5.2 | 6.1 | 5.9 (1.2) | 5.9 (1.3) | 5.1 (1.1) |
| Asp 101 | 7.0 | 7.9 | 6.1 (1.5) | 6.4 (1.3) | 7.7 (1.4) |
| Asn 103 | 8.2 | 6.6 | 5.4 (1.4) | 6.2 (1.3) | 6.8 (1.2) |
| Met 105 | 7.4 | 3.8 | 7.0 (1.4) | 4.3 (1.0) | 4.4 (1.2) |
| Ala 107 | 4.2 | 4.2 | 5.2 (1.3) | 5.4 (1.3) | 5.6 (1.4) |
| Trp 108 | 9.6 | 8.7 | 5.9 (1.3) | 8.8 (1.1) | 7.8 (1.8) |
| Val 109 | 4.0 | 3.8 | 4.7 (1.0) | 4.1 (1.0) | 4.4 (0.9) |
| Trp 111 | 7.1 | 4.8 | 5.4 (1.2) | 6.6 (1.4) | 6.4 (1.3) |
| Arg 112 | 4.5 | 4.3 | 4.1 (1.1) | 4.9 (1.1) | 6.1 (1.4) |
| Asn 113 | 5.8 | 7.2 | 7.3 (1.8) | 5.8 (1.1) | 7.1 (2.0) |
| Arg 114 | 9.6 | 9.6 | 7.3 (1.9) | 6.8 (0.2) | 3.6 (1.1) |
| Cys 115 | 9.8 (9.7) | 9.6 | 6.2 (1.7) | 5.4 (1.4) | 7.1 (1.5) |
| Thr 118 | 9.8 (9.7) | 9.5 | 6.8 (1.9) | 5.3 (1.6) | 3.9 (1.3) |
| Asp 119 | 6.7 | 6.5 | 5.9 (1.8) | 8.0 (1.9) | 4.9 (1.6) |
| Val 120 | 4.6 | 5.0 | 6.4 (2.2) | 4.3 (1.2) | 4.7 (1.5) |
| Gln 121 | 5.0 | 4.0 | 4.2 (1.2) | 3.8 (1.0) | 4.3 (1.3) |
| Ala 122 | 3.7 | 3.5 | 4.9 (1.3) | 5.6 (1.3) | 5.1 (1.3) |
| Trp 123 | 5.4 | 5.8 | 6.0 (1.6) | 7.0 (1.7) | 6.0 (1.8) |
| Ile 124 | 10.6 (9.7) | 9.6 | 6.7 (1.6) | 8.1 (1.3) | 8.2 (1.4) |
| Arg 125 | 4.4 | 4.7 | 5.9 (1.9) | 3.9 (1.2) | 3.9 (1.3) |
| Cys 127 | 7.7 | 7.6 | 5.6 (1.8) | 6.4 (1.3) | 6.7 (1.2) |
| Arg 128 | 8.0 | 6.0 | 6.8 (1.9) | 5.7 (1.2) | 5.3 (1.1) |
| Leu 129 | 9.0 | 9.6 | 7.5 (1.8) | 5.4 (1.6) | 5.0 (1.4) |
