# Supplementary material for: Molecular dynamics simulation or structure refinement of proteins: are solvent molecules required? A case study using hen lysozyme
Source: Eur Biophys J. 2022 Mar 18;51(3):265–82. doi: 10.1007/s00249-022-01593-1 (PMC9035012; doi:10.1007/s00249-022-01593-1)
Supplement: Supplementary file 2 — Supplementary file2 (DOCX 18 kb) [file 249_2022_1593_MOESM2_ESM.docx]

Table S3. Experimentally stereo-specifically unassigned backbone *^3^J_HNHα_*-coupling values (22) in Hz derived from NMR measurements and values calculated from the *2VB1* X-ray structure, the MD simulation in explicit water using the GROMOS 54A7 force field (*MD_water*), the SD simulations in vacuo using the GROMOS 54B7 force field without (*SD_nowater*) and with (*SD_implicit*) a SASA implicit-solvation term. Experimental values from Table II of (Smith et al. 1991). The root-mean-square fluctuations (RMSF) of the *^3^J*-couplings in the simulations are given within parentheses. Stereo-specific assignments were taken from (Smith et al. 2021a).

| Residue | Experimental value | *X-ray structure 2VB1* | *MD_water* | *SD_nowater* | *SD_implicit* |
| --- | --- | --- | --- | --- | --- |
| Gly 4 α2 Re | 8.0 | 7.3 | 7.5 (1.8) | 4.5 (1.6) | 3.7 (1.1) |
| α3 Si | 6.1 | 5.9 | 4.9 (1.7) | 6.5 (0.6) | 6.6 (0.5) |
| Gly 16 α2 Re | 6.1 | 5.9 | 5.3 (1.7) | 6.1 (1.1) | 4.7 (1.9) |
| α3 Si | 6.2 | 7.4 | 6.3 (2.0) | 6.2 (1.6) | 6.5 (2.1) |
| Gly 22 α2 Re | 6.0 | 6.2 | 6.1 (0.9) | 6.4 (0.5) | 5.7 (1.4) |
| α3 Si | 6.8 | 6.9 | 6.4 (1.6) | 5.9 (1.3) | 5.9 (1.7) |
| Gly 26 α2 Re | 3.3 | 4.4 | 3.9 (1.0) | 3.9 (1.0) | 4.5 (1.0) |
| α3 Si | 6.2 | 6.9 | 6.7 (0.3) | 6.7 (0.2) | 6.8 (0.2) |
| Gly 49 α2 Re | 5.3 | 5.6 | 3.1 (1.2) | 6.5 (0.7) | 6.6 (0.5) |
| α3 Si | 8.0 | 7.8 | 9.1 (1.0) | 4.5 (1.5) | 4.8 (1.4) |
| Gly 67 α2 Re | 5.5 | 6.6 | 6.0 (1.6) | 6.6 (0.4) | 6.5 (0.5) |
| α3 Si | 6.2 | 5.8 | 6.4 (1.4) | 5.1 (1.3) | 5.6 (1.3) |
| Gly 71 α2 Re | 5.9 | 6.7 | 6.2 (1.1) | 4.6 (1.9) | 4.2 (1.5) |
| α3 Si | 5.7 | 5.4 | 5.5 (1.8) | 7.1 (1.7) | 8.4 (1.3) |
| Gly 102 α2 Re | 6.2 | 5.6 | 4.2 (1.1) | 6.5 (0.5) | 6.7 (0.4) |
| α3 Si | 6.4 | 7.7 | 6.7 (0.3) | 5.7 (1.2) | 4.2 (1.2) |
| Gly 104 α2 Re | 6.4 | 6.5 | 6.7 (0.4) | 4.5 (1.2) | 6.7 (0.4) |
| α3 Si | 3.9 | 6.3 | 4.5 (1.2) | 6.7 (0.4) | 4.6 (1.3) |
| Gly 117 α2 Re | 6.2 | 6.2 | 5.3 (1.6) | 6.5 (0.7) | 6.6 (0.3) |
| α3 Si | 6.5 | 6.9 | 6.1 (1.3) | 5.4 (1.4) | 5.3 (1.2) |
| Gly 126 α2 Re | 6.8 | 6.2 | 6.5 (1.1) | 6.3 (0.6) | 6.2 (0.7) |
| α3 Si | 5.6 | 6.9 | 5.3 (1.8) | 6.4 (1.2) | 6.5 (1.2) |
