# Supplementary material for: Molecular dynamics simulation or structure refinement of proteins: are solvent molecules required? A case study using hen lysozyme
Source: Eur Biophys J. 2022 Mar 18;51(3):265–82. doi: 10.1007/s00249-022-01593-1 (PMC9035012; doi:10.1007/s00249-022-01593-1)
Supplement: Supplementary file 3 — Supplementary file3 (DOCX 14 kb) [file 249_2022_1593_MOESM3_ESM.docx]

Table S4. Side-chain *^3^J_HαHβ_*-coupling values (58) in Hz derived and stereo-specifically assigned based on NMR measurements and values calculated from the *2VB1* X-ray structure, the MD simulation in explicit water using the GROMOS 54A7 force field (*MD_water*), the SD simulations in vacuo using the GROMOS 54B7 force field without (*SD_nowater*) and with (*SD_implicit*) a SASA implicit-solvation term. Experimental values from Tables III and IV of (Smith et al. 1991). The root-mean-square fluctuations (RMSF) of the *^3^J*-couplings in the simulations are given within parentheses.

| Residue | Experimental value | *X-ray structure 2VB1* | *MD_water* | *SD_nowater* | *SD_implicit* |
| --- | --- | --- | --- | --- | --- |
| Val 2 | 10.8 | 12.9 | 9.3 (4.6) | 7.0 (5.0) | 8.5 (4.9) |
| Cys 6 β_2_ | 11.5 | 12.7 | 12.6 (0.6) | 11.7 (1.4) | 11.4 (3.3) |
| β_3_ | 3.5 | 2.5 | 3.4 (1.1) | 2.7 (1.5) | 4.5 (3.0) |
| His 15 β_2_ | 11.2 | 12.9 | 11.9 (1.3) | 6.6 (5.0) | 12.1 (2.0) |
| β_3_ | 2.6 | 3.1 | 2.4 (0.9) | 7.1 (4.2) | 3.2 (1.6) |
| Asp 18 β_2_ | 4.2 | 3.2 | 5.1 (4.1) | 5.6 (4.4) | 5.2 (4.1) |
| β_3_ | 11.0 | 12.9 | 7.5 (4.3) | 8.6 (4.6) | 7.9 (4.6) |
| Tyr 20 β_2_ | 2.3 | 3.0 | 7.2 (4.8) | 2.4 (0.6) | 3.7 (2.3) |
| β_3_ | 11.7 | 12.9 | 6.9 (4.5) | 12.1 (1.0) | 8.5 (4.6) |
| Tyr 23 β_2_ | 10.9 | 12.5 | 12.6 (0.5) | 12.6 (0.5) | 12.5 (0.5) |
| β_3_ | 2.7 | 2.2 | 3.1 (1.0) | 3.4 (1.1) | 3.0 (0.9) |
| Asn 27 β_2_ | 10.3 | 11.6 | 3.6 (2.9) | 5.0 (4.4) | 12.4 (0.6) |
| β_3_ | 2.4 | 1.8 | 4.3 (1.2) | 7.7 (3.4) | 4.8 (1.0) |
| Val 29 | 11.1 | 12.8 | 10.1 (4.3) | 11.9 (2.5) | 12.3 (1.1) |
| Cys 30 β_2_ | 5.3 | 3.0 | 3.2 (1.0) | 2.7 (0.8) | 3.4 (1.0) |
| β_3_ | 10.8 | 12.9 | 12.6 (0.6) | 12.5 (0.6) | 12.7 (0.3) |
| Phe 34 β_2_ | 10.7 | 12.8 | 10.7 (3.3) | 12.2 (0.9) | 12.2 (1.6) |
| β_3_ | 5.0 | 2.6 | 3.5 (3.1) | 2.5 (0.8) | 3.0 (1.5) |
| Asn 39 β_2_ | 4.5 | 2.6 | 2.5 (1.0) | 2.8 (0.9) | 4.7 (2.6) |
| β_3_ | 10.8 | 12.7 | 12.0 (1.1) | 11.9 (1.5) | 8.1 (5.0) |
| Thr 40 | 4.5 | 3.5 | 2.7 (0.8) | 3.2 (0.9) | 2.3 (0.6) |
| Thr 43 | 3.7 | 3.0 | 3.4 (2.6) | 3.1 (1.5) | 4.5 (4.0) |
| Asn 46 β_2_ | 11.2 | 12.9 | 2.6 (2.0) | 2.3 (0.7) | 10.8 (2.6) |
| β_3_ | 4.7 | 3.4 | 9.2 (1.9) | 11.5 (1.6) | 2.6 (1.6) |
| Thr 47 | 2.6 | 3.6 | 3.0 (1.5) | 2.5 (1.0) | 2.8 (0.9) |
| Asp 48 β_2_ | 2.6 | 5.3 | 4.2 (1.1) | 4.5 (2.7) | 8.4 (4.4) |
| β_3_ | 3.7 | 2.1 | 2.9 (1.0) | 3.3 (1.3) | 4.0 (3.1) |
| Thr 51 | 9.3 | 12.9 | 5.6 (4.6) | 2.5 (0.7) | 2.4 (0.8) |
| Asp 52 β_2_ | 11.6 | 12.6 | 12.6 (0.4) | 12.5 (0.7) | 12.5 (0.6) |
| β_3_ | 3.6 | 4.7 | 3.8 (1.1) | 2.8 (0.9) | 3.6 (1.2) |
| Tyr 53 β_2_ | 10.4 | 12.7 | 12.1 (0.9) | 12.6 (0.5) | 12.4 (0.6) |
| β_3_ | 3.0 | 2.5 | 2.4 (0.7) | 4.0 (1.2) | 2.6 (0.8) |
| Asn 59 β_2_ | 5.4 | 2.8 | 2.3 (0.5) | 6.1 (2.1) | 4.1 (2.7) |
| β_3_ | 11.3 | 12.8 | 12.3 (0.6) | 4.5 (3.7) | 11.6 (2.6) |
| Arg 61 β_2_ | 5.7 | 2.6 | 5.5 (4.3) | 7.8 (4.7) | 11.5 (2.6) |
| β_3_ | 10.8 | 12.7 | 8.4 (4.6) | 5.8 (4.3) | 3.2 (2.1) |
| Asp 66 β_2_ | 5.1 | 4.5 | 3.2 (1.2) | 2.4 (1.3) | 6.5 (4.5) |
| β_3_ | 4.5 | 2.5 | 11.5 (2.9) | 10.5 (2.7) | 6.4 (4.1) |
| Thr 69 | 9.3 | 12.9 | 6.1 (4.6) | 2.5 (0.8) | 2.7 (1.4) |
| Leu 75 β_2_ | 12.4 | 12.9 | 11.5 (2.4) | 11.6 (2.0) | 11.3 (2.5) |
| β_3_ | 2.1 | 3.1 | 3.0 (1.8) | 2.9 (1.4) | 3.0 (2.1) |
| Asp 87 β_2_ | 5.1 | 4.1 | 3.3 (1.3) | 6.3 (4.1) | 7.8 (4.8) |
| β_3_ | 11.5 | 12.8 | 12.2 (1.1) | 4.7 (3.8) | 6.6 (4.7) |
| Ile 88 | 4.5 | 4.5 | 4.3 (3.8) | 5.1 (4.4) | 8.7 (4.8) |
| Thr 89 | 9.5 | 12.8 | 4.8 (3.4) | 2.5 (0.9) | 6.9 (4.6) |
| Val 92 | 10.1 | 12.5 | 9.6 (4.5) | 11.6 (3.0) | 5.1 (4.5) |
| Cys 94 β_2_ | 4.0 | 2.9 | 2.6 (0.7) | 3.2 (0.9) | 3.1 (1.0) |
| β_3_ | 12.2 | 12.8 | 12.4 (0.6) | 12.6 (0.4) | 12.5 (0.5) |
| Val 99 | 6.3 | 12.8 | 3.0 (1.6) | 3.0 (1.9) | 9.1 (4.5) |
| Val 109 | 8.0 | 3.2 | 9.0 (4.7) | 5.6 (4.5) | 4.1 (3.3) |
| Thr 118 | 4.2 | 5.2 | 2.9 (1.1) | 2.7 (0.9) | 2.9 (0.9) |
| Asp 119 β_2_ | 4.9 | 3.1 | 4.5 (3.8) | 8.2 (4.9) | 7.2 (4.3) |
| β_3_ | 11.7 | 12.9 | 10.1 (3.8) | 6.1 (4.4) | 3.1 (1.4) |
| Trp 123 β_2_ | 10.6 | 11.9 | 12.2 (1.2) | 12 (1.2) | 12.5 (0.5) |
| β_3_ | 2.9 | 1.9 | 3.7 (1.6) | 5.2 (1.6) | 3.8 (1.2) |
| Ile 124 | 4.6 | 4.4 | 4.1 (2.7) | 3.4 (2.3) | 4.4 (3.6) |
| Cys 127 β_2_ | 11.6 | 12.9 | 12.6 (0.6) | 9.3 (4.5) | 4.4 (3.1) |
| β_3_ | 4.8 | 3.3 | 3.2 (1.0) | 3.5 (1.1) | 3.6 (1.2) |
