# Supplementary material for: Molecular dynamics simulation or structure refinement of proteins: are solvent molecules required? A case study using hen lysozyme
Source: Eur Biophys J. 2022 Mar 18;51(3):265–82. doi: 10.1007/s00249-022-01593-1 (PMC9035012; doi:10.1007/s00249-022-01593-1)
Supplement: Supplementary file 4 — Supplementary file4 (DOCX 17 kb) [file 249_2022_1593_MOESM4_ESM.docx]

Table S5. Experimentally stereo-specifically unassigned side-chain *^3^J_HαHβ_*-coupling values (40) in Hz derived from NMR measurements and values calculated from the *2VB1* X-ray structure, the MD simulation in explicit water using the GROMOS 54A7 force field (*MD_water*), the SD simulations in vacuo using the GROMOS 54B7 force field without (*SD_nowater*) and with (*SD_implicit*) a SASA implicit-solvation term. Experimental values from Table III of (Smith et al. 1991). The root-mean-square fluctuations (RMSF) of the *^3^J*-couplings in the simulations are given within parentheses. Stereo-specific assignments were taken from (Smith et al. 2021a). *Only Glu 7 could not be stereo-specifically assigned.

| Residue | Experimental value | *X-ray structure 2VB1* | *MD_water* | *SD_nowater* | *SD_implicit* |
| --- | --- | --- | --- | --- | --- |
| Glu 7 β_2_ | *6.7 | 12.7 | 7.8 (4.7) | 10.6 (3.7) | 10.4 (4.0) |
| β_3_ | *6.4 | 2.6 | 7.0 (4.6) | 4.1 (3.3) | 4.5 (3.6) |
| Lys 13 β_2_ | 5.1 | 3.1 | 5.7 (4.5) | 8.5 (4.6) | 3.6 (2.9) |
| β_3_ | 9.2 | 12.9 | 8.6 (4.7) | 5.8 (4.6) | 11.1 (3.2) |
| Asn 19 β_2_ | 7.3 | 1.8 | 8.3 (4.6) | 6.7 (3.1) | 5.6 (3.7) |
| β_3_ | 6.4 | 11.4 | 5.9 (4.6) | 4.5 (1.9) | 7.4 (4.1) |
| Trp 28 β_2_ | 10.7 | 12.8 | 12.6 (0.5) | 12.2 (0.8) | 12.4 (0.7) |
| β_3_ | 4.1 | 2.8 | 3.9 (1.2) | 2.7 (0.9) | 3.2 (1.1) |
| Asn 37 β_2_ | 8.1 | 1.9 | 9.1 (4.6) | 12.4 (0.6) | 11.4 (2.9) |
| β_3_ | 4.2 | 10.0 | 5.1 (3.9) | 4.5 (1.3) | 4.8 (1.7) |
| Arg 45 β_2_ | 6.9 | 12.8 | 8.0 (4.8) | 7.9 (4.7) | 10.5 (3.7) |
| β_3_ | 6.7 | 2.8 | 6.4 (4.6) | 6.2 (4.6) | 4.0 (3.3) |
| Cys 64 β_2_ | 4.6 | 3.6 | 4.4 (1.0) | 3.8 (0.8) | 3.5 (0.9) |
| β_3_ | 2.7 | 3.2 | 2.7 (0.7) | 3.2 (0.7) | 3.4 (0.9) |
| Asn 65 β_2_ | 4.5 | 3.1 | 4.2 (3.1) | 2.5 (0.8) | 2.5 (1.1) |
| β_3_ | 11.4 | 12.9 | 11.3 (3.2) | 12.2 (0.7) | 10.8 (2.6) |
| Arg 68 β_2_ | 6.5 | 12.8 | 10.1 (4.1) | 7.1 (4.5) | 6.2 (4.4) |
| β_3_ | 4.8 | 2.6 | 4.5 (3.6) | 3.8 (2.3) | 4.6 (3.1) |
| Ser 72 β_2_ | 5.4 | 4.9 | 7.5 (4.7) | 9.0 (2.9) | 4.9 (4.3) |
| β_3_ | 7.6 | 12.5 | 5.2 (3.6) | 3.1 (2.2) | 9.3 (4.4) |
| Asn 74 β_2_ | 10.5 | 2.2 | 11.3 (3.2) | 2.0 (0.7) | 5.7 (4.2) |
| β_3_ | 3.9 | 12.4 | 4.0 (2.2) | 10.7 (1.3) | 7.3 (4.6) |
| Asn 77 β_2_ | 8.3 | 2.0 | 10.8 (3.4) | 11.6 (1.4) | 12.0 (1.5) |
| β_3_ | 5.9 | 12.2 | 3.8 (2.6) | 2.3 (1.1) | 2.7 (1.1) |
| Ser 85 β_2_ | 5.7 | 12.9 | 4.9 (3.9) | 3.3 (1.4) | 3.8 (1.8) |
| β_3_ | 7.4 | 3.5 | 9.6 (4.3) | 11.1 (3.6) | 8.6 (4.9) |
| Ser 86 β_2_ | 6.4 | 12.8 | 8.7 (4.6) | 11.1 (3.3) | 4.4 (3.7) |
| β_3_ | 4.1 | 2.8 | 3.9 (2.1) | 3.3 (1.3) | 4.7 (1.6) |
| Asn 93 β_2_ | 10.8 | 12.7 | 10.7 (3.6) | 10.5 (3.8) | 12.3 (1.0) |
| β_3_ | 3.5 | 2.5 | 4.1 (3.6) | 3.9 (3.0) | 4.7 (1.3) |
| Ser 100 β_2_ | 7.7 | 6.1 | 7.7 (4.6) | 4.6 (3.5) | 10.4 (3.9) |
| β_3_ | 4.0 | 1.8 | 5.2 (4.0) | 3.8 (1.2) | 3.9 (1.7) |
| Asp 101 β_2_ | 5.6 | 10.5 | 2.5 (0.8) | 8.6 (4.7) | 9.0 (4.5) |
| β_3_ | 6.6 | 1.8 | 12.2 (0.9) | 6.3 (4.6) | 4.8 (4.0) |
| Asn 106 β_2_ | 10.5 | 9.3 | 4.7 (2.8) | 11.9 (1.7) | 12.6 (0.5) |
| β_3_ | 3.6 | 2.1 | 3.9 (2.7) | 2.6 (1.0) | 3.5 (1.1) |
| Arg 125 β_2_ | 7.9 | 2.6 | 10.4 (3.7) | 11.4 (3.0) | 11.8 (2.6) |
| β_3_ | 6.1 | 12.7 | 4.0 (3.3) | 3.5 (2.4) | 3.3 (1.8) |
| Arg 128 β_2_ | 7.9 | 12.9 | 9.3 (4.4) | 12.5 (0.7) | 12.3 (0.7) |
| β_3_ | 7.2 | 3.2 | 4.8 (3.9) | 2.9 (0.9) | 2.6 (0.8) |
