# Supplementary material for: Molecular dynamics simulation or structure refinement of proteins: are solvent molecules required? A case study using hen lysozyme
Source: Eur Biophys J. 2022 Mar 18;51(3):265–82. doi: 10.1007/s00249-022-01593-1 (PMC9035012; doi:10.1007/s00249-022-01593-1)
Supplement: Supplementary file 5 — Supplementary file5 (DOCX 16 kb) [file 249_2022_1593_MOESM5_ESM.docx]

Table S6: Backbone *S*^2^*_NH_* order-parameter values (121) derived from relaxation measurements (Buck et al. 1995) and values calculated from the MD simulation in explicit water using the GROMOS 54A7 force field (*MD_water*), the SD simulations in vacuo using the GROMOS 54B7 force field without (*SD_nowater*) and with (*SD_implicit*) a SASA implicit-solvation term. Experimental order-parameter values larger than 0.95 were set to 0.95 (second column between brackets).

| Residue | Experimental value | *MD_water* | *SD_nowater* | *SD_implicit* |
| --- | --- | --- | --- | --- |
| Val 2 | 0.83 | 0.73 | 0.55 | 0.65 |
| Phe 3 | 0.83 | 0.81 | 0.80 | 0.80 |
| Gly 4 | 0.83 | 0.66 | 0.79 | 0.82 |
| Arg 5 | 0.85 | 0.76 | 0.78 | 0.80 |
| Cys 6 | 0.86 | 0.83 | 0.80 | 0.82 |
| Glu 7 | 0.88 | 0.83 | 0.86 | 0.88 |
| Leu 8 | 0.89 | 0.89 | 0.82 | 0.84 |
| Ala 9 | 0.93 | 0.89 | 0.84 | 0.89 |
| Ala 10 | 0.89 | 0.89 | 0.87 | 0.90 |
| Ala 11 | 0.89 | 0.89 | 0.85 | 0.88 |
| Met 12 | 0.91 | 0.89 | 0.84 | 0.89 |
| Lys 13 | 0.92 | 0.89 | 0.86 | 0.88 |
| Arg 14 | 0.82 | 0.89 | 0.85 | 0.87 |
| His 15 | 0.84 | 0.82 | 0.79 | 0.79 |
| Leu 17 | 0.89 | 0.53 | 0.71 | 0.78 |
| Asp 18 | 0.86 | 0.55 | 0.83 | 0.77 |
| Asn 19 | 0.84 | 0.71 | 0.61 | 0.70 |
| Tyr 20 | 0.85 | 0.46 | 0.69 | 0.56 |
| Arg 21 | 0.89 | 0.72 | 0.76 | 0.73 |
| Gly 22 | 0.99 (0.95) | 0.71 | 0.68 | 0.64 |
| Tyr 23 | 0.88 | 0.57 | 0.77 | 0.75 |
| Ser 24 | 0.89 | 0.68 | 0.75 | 0.78 |
| Leu 25 | 0.87 | 0.73 | 0.87 | 0.86 |
| Gly 26 | 0.91 | 0.85 | 0.81 | 0.82 |
| Asn 27 | 0.94 | 0.84 | 0.85 | 0.85 |
| Trp 28 | 0.87 | 0.86 | 0.85 | 0.87 |
| Val 29 | 0.90 | 0.90 | 0.89 | 0.89 |
| Ala 31 | 0.93 | 0.90 | 0.89 | 0.85 |
| Ala 32 | 0.94 | 0.81 | 0.88 | 0.83 |
| Lys 33 | 0.91 | 0.88 | 0.87 | 0.89 |
| Phe 34 | 0.92 | 0.84 | 0.87 | 0.85 |
| Glu 35 | 0.88 | 0.83 | 0.82 | 0.85 |
| Ser 36 | 0.86 | 0.73 | 0.62 | 0.80 |
| Asn 37 | 0.96 (0.95) | 0.76 | 0.74 | 0.69 |
| Phe 38 | 0.90 | 0.85 | 0.76 | 0.85 |
| Asn 39 | 0.89 | 0.72 | 0.73 | 0.73 |
| Thr 40 | 0.91 | 0.85 | 0.85 | 0.84 |
| Gln 41 | 0.86 | 0.82 | 0.84 | 0.76 |
| Ala 42 | 0.87 | 0.72 | 0.82 | 0.66 |
| Thr 43 | 0.83 | 0.61 | 0.80 | 0.67 |
| Asn 44 | 0.83 | 0.67 | 0.78 | 0.47 |
| Arg 45 | 0.78 | 0.71 | 0.76 | 0.67 |
| Asn 46 | 0.83 | 0.75 | 0.81 | 0.74 |
| Thr 47 | 0.78 | 0.80 | 0.79 | 0.75 |
| Asp 48 | 0.77 | 0.79 | 0.72 | 0.71 |
| Gly 49 | 0.82 | 0.72 | 0.75 | 0.77 |
| Thr 51 | 0.89 | 0.66 | 0.77 | 0.75 |
| Asp 52 | 0.89 | 0.83 | 0.86 | 0.76 |
| Tyr 53 | 0.87 | 0.84 | 0.78 | 0.84 |
| Gly 54 | 0.91 | 0.86 | 0.81 | 0.79 |
| Ile 55 | 0.94 | 0.84 | 0.83 | 0.84 |
| Leu 56 | 0.92 | 0.89 | 0.66 | 0.78 |
| Gln 57 | 0.94 | 0.86 | 0.60 | 0.84 |
| Ile 58 | 0.90 | 0.86 | 0.84 | 0.70 |
| Asn 59 | 0.91 | 0.86 | 0.68 | 0.74 |
| Ser 60 | 0.93 | 0.87 | 0.84 | 0.85 |
| Arg 61 | 0.95 | 0.77 | 0.78 | 0.86 |
| Trp 62 | 0.85 | 0.85 | 0.80 | 0.81 |
| Trp 63 | 0.90 | 0.74 | 0.87 | 0.66 |
| Cys 64 | 0.91 | 0.81 | 0.85 | 0.73 |
| Asn 65 | 0.86 | 0.84 | 0.89 | 0.82 |
| Asp 66 | 0.89 | 0.61 | 0.81 | 0.69 |
| Gly 67 | 0.85 | 0.56 | 0.77 | 0.75 |
| Arg 68 | 0.78 | 0.63 | 0.72 | 0.75 |
| Thr 69 | 0.76 | 0.78 | 0.60 | 0.54 |
| Gly 71 | 0.72 | 0.62 | 0.43 | 0.66 |
| Ser 72 | 0.76 | 0.62 | 0.74 | 0.86 |
| Arg 73 | 0.88 | 0.79 | 0.74 | 0.68 |
| Asn 74 | 0.87 | 0.84 | 0.72 | 0.54 |
| Leu 75 | 0.94 | 0.76 | 0.78 | 0.67 |
| Cys 76 | 0.92 | 0.86 | 0.88 | 0.87 |
| Asn 77 | 0.90 | 0.86 | 0.85 | 0.77 |
| Ile 78 | 0.91 | 0.84 | 0.86 | 0.86 |
| Cys 80 | 0.91 | 0.86 | 0.90 | 0.85 |
| Ser 81 | 0.86 | 0.86 | 0.84 | 0.83 |
| Ala 82 | 0.88 | 0.81 | 0.81 | 0.83 |
| Leu 83 | 0.83 | 0.83 | 0.83 | 0.84 |
| Leu 84 | 0.83 | 0.81 | 0.85 | 0.81 |
| Ser 85 | 0.55 | 0.45 | 0.70 | 0.70 |
| Ser 86 | 0.80 | 0.75 | 0.74 | 0.78 |
| Asp 87 | 0.80 | 0.36 | 0.82 | 0.81 |
| Ile 88 | 0.80 | 0.64 | 0.82 | 0.85 |
| Thr 89 | 0.92 | 0.81 | 0.84 | 0.77 |
| Ala 90 | 0.91 | 0.85 | 0.84 | 0.80 |
| Ser 91 | 0.85 | 0.85 | 0.86 | 0.86 |
| Val 92 | 0.93 | 0.88 | 0.88 | 0.88 |
| Asn 93 | 0.93 | 0.88 | 0.89 | 0.86 |
| Cys 94 | 0.92 | 0.90 | 0.89 | 0.89 |
| Ala 95 | 0.92 | 0.91 | 0.89 | 0.89 |
| Lys 96 | 0.92 | 0.90 | 0.88 | 0.89 |
| Lys 97 | 0.94 | 0.85 | 0.83 | 0.87 |
| Ile 98 | 0.92 | 0.88 | 0.84 | 0.88 |
| Ser 100 | 0.89 | 0.82 | 0.48 | 0.84 |
| Asp 101 | 0.85 | 0.74 | 0.79 | 0.85 |
| Gly 102 | 0.72 | 0.75 | 0.79 | 0.83 |
| Asn 103 | 0.52 | 0.78 | 0.82 | 0.82 |
| Gly 104 | 0.81 | 0.76 | 0.84 | 0.84 |
| Met 105 | 0.88 | 0.84 | 0.81 | 0.78 |
| Asn 106 | 0.96 (0.95) | 0.82 | 0.84 | 0.81 |
| Ala 107 | 0.91 | 0.83 | 0.85 | 0.76 |
| Trp 108 | 0.84 | 0.84 | 0.81 | 0.55 |
| Val 109 | 0.85 | 0.83 | 0.80 | 0.89 |
| Trp 111 | 0.84 | 0.82 | 0.85 | 0.81 |
| Arg 112 | 0.89 | 0.82 | 0.82 | 0.85 |
| Asn 113 | 0.89 | 0.76 | 0.87 | 0.59 |
| Arg 114 | 0.87 | 0.63 | 0.82 | 0.82 |
| Cys 115 | 0.79 | 0.72 | 0.87 | 0.86 |
| Lys 116 | 0.84 | 0.58 | 0.81 | 0.82 |
| Gly 117 | 0.81 | 0.55 | 0.73 | 0.72 |
| Thr 118 | 0.72 | 0.60 | 0.79 | 0.82 |
| Asp 119 | 0.80 | 0.65 | 0.75 | 0.71 |
| Val 120 | 0.80 | 0.67 | 0.75 | 0.75 |
| Gln 121 | 0.91 | 0.76 | 0.85 | 0.82 |
| Ala 122 | 0.92 | 0.74 | 0.82 | 0.83 |
| Trp 123 | 0.90 | 0.70 | 0.71 | 0.75 |
| Ile 124 | 0.90 | 0.78 | 0.81 | 0.82 |
| Arg 125 | 0.87 | 0.60 | 0.75 | 0.76 |
| Gly 126 | 0.82 | 0.59 | 0.82 | 0.82 |
| Cys 127 | 0.77 | 0.54 | 0.72 | 0.76 |
| Arg 128 | 0.76 | 0.45 | 0.82 | 0.81 |
| Leu 129 | 0.60 | 0.25 | 0.77 | 0.80 |
