# Supplementary material for: Molecular dynamics simulation or structure refinement of proteins: are solvent molecules required? A case study using hen lysozyme
Source: Eur Biophys J. 2022 Mar 18;51(3):265–82. doi: 10.1007/s00249-022-01593-1 (PMC9035012; doi:10.1007/s00249-022-01593-1)
Supplement: Supplementary file 6 — Supplementary file6 (DOCX 18 kb) [file 249_2022_1593_MOESM6_ESM.docx]

Table S7. Side-chain *S^2^_CH_* order-parameter values (51) derived from relaxation measurements (Moorman et al. 2012) and values calculated from the MD simulation in explicit water using the GROMOS 54A7 force field (*MD_water*), the SD simulations in vacuo using the GROMOS 54B7 force field without (*SD_nowater*) and with (*SD_implicit*) a SASA implicit-solvation term. Order-parameter target values larger than 0.95 were set to 0.95 (second column between brackets).

| Residue and methyl group | Experimental value | *MD_water* | *SD_nowater* | *SD_implicit* |
| --- | --- | --- | --- | --- |
| Val 2 CG2 | 0.598 | 0.39 | 0.34 | 0.46 |
| Leu 8 CD1 | 0.767 | 0.58 | 0.53 | 0.72 |
| Leu 8 CD2 | 0.803 | 0.63 | 0.47 | 0.73 |
| Ala 9 CB | 1.0 (0.95) | 0.93 | 0.91 | 0.94 |
| Ala 10 CB | 0.901 | 0.91 | 0.91 | 0.93 |
| Ala 11 CB | 0.861 | 0.91 | 0.90 | 0.92 |
| Met 12 CE | 0.812 | 0.33 | 0.32 | 0.36 |
| Leu 17 CD1 | 0.630 | 0.46 | 0.35 | 0.34 |
| Leu 17 CD2 | 0.632 | 0.49 | 0.36 | 0.40 |
| Leu 25 CD1 | 1.0 (0.95) | 0.40 | 0.52 | 0.41 |
| Leu 25 CD2 | 0.609 | 0.42 | 0.59 | 0.41 |
| Val 29 CG1 | 0.871 | 0.57 | 0.78 | 0.86 |
| Val 29 CG2 | 0.791 | 0.57 | 0.77 | 0.86 |
| Ala 31 CB | 0.98 (0.95) | 0.94 | 0.94 | 0.94 |
| Thr 43 CG2 | 0.361 | 0.68 | 0.79 | 0.72 |
| Thr 47 CG2 | 0.327 | 0.73 | 0.74 | 0.78 |
| Thr 51 CG2 | 0.778 | 0.49 | 0.88 | 0.82 |
| Ile 55 CG2 | 0.739 | 0.49 | 0.70 | 0.90 |
| Ile 55 CD | 0.323 | 0.55 | 0.38 | 0.43 |
| Leu 56 CD1 | 0.734 | 0.79 | 0.50 | 0.50 |
| Leu 56 CD2 | 0.681 | 0.75 | 0.58 | 0.47 |
| Ile 58 CG2 | 1.0 (0.95) | 0.84 | 0.92 | 0.72 |
| Ile 58 CD | 0.160 | 0.81 | 0.83 | 0.58 |
| Thr 69 CG2 | 0.98 (0.95) | 0.72 | 0.83 | 0.82 |
| Leu 75 CD1 | 0.590 | 0.62 | 0.50 | 0.34 |
| Ile 78 CG2 | 0.810 | 0.85 | 0.68 | 0.74 |
| Ile 78 CD | 0.416 | 0.43 | 0.40 | 0.45 |
| Leu 83 CD1 | 0.884 | 0.68 | 0.81 | 0.60 |
| Leu 83 CD2 | 0.783 | 0.66 | 0.74 | 0.60 |
| Leu 84 CD1 | 1.0 (0.95) | 0.46 | 0.41 | 0.45 |
| Leu 84 CD2 | 0.879 | 0.45 | 0.39 | 0.43 |
| Ile 88 CG2 | 0.697 | 0.55 | 0.59 | 0.61 |
| Ile 88 CD | 0.722 | 0.27 | 0.39 | 0.46 |
| Thr 89 CG2 | 1.0 (0.95) | 0.71 | 0.84 | 0.43 |
| Ala 90 CB | 0.919 | 0.91 | 0.91 | 0.86 |
| Val 92 CG1 | 0.764 | 0.63 | 0.72 | 0.70 |
| Val 92 CG2 | 0.707 | 0.61 | 0.71 | 0.69 |
| Ala 95 CB | 0.680 | 0.94 | 0.94 | 0.93 |
| Ile 98 CG2 | 0.740 | 0.90 | 0.79 | 0.87 |
| Ile 98 CD | 0.815 | 0.89 | 0.82 | 0.87 |
| Val 99 CG1 | 0.487 | 0.85 | 0.75 | 0.74 |
| Val 99 CG2 | 0.517 | 0.85 | 0.76 | 0.74 |
| Met 105 CE | 0.630 | 0.80 | 0.56 | 0.71 |
| Ala 107 CB | 0.832 | 0.88 | 0.90 | 0.87 |
| Val 109 CG2 | 0.354 | 0.36 | 0.33 | 0.69 |
| Val 120 CG1 | 0.660 | 0.69 | 0.84 | 0.53 |
| Ala 122 CB | 0.879 | 0.78 | 0.89 | 0.90 |
| Ile 124 CG2 | 0.753 | 0.75 | 0.67 | 0.76 |
| Ile 124 CD | 0.351 | 0.48 | 0.57 | 0.83 |
| Leu 129 CD1 | 0.525 | 0.12 | 0.45 | 0.40 |
| Leu 129 CD2 | 0.507 | 0.11 | 0.41 | 0.35 |
