# Supplementary material for: Molecular dynamics simulation or structure refinement of proteins: are solvent molecules required? A case study using hen lysozyme
Source: Eur Biophys J. 2022 Mar 18;51(3):265–82. doi: 10.1007/s00249-022-01593-1 (PMC9035012; doi:10.1007/s00249-022-01593-1)
Supplement: Supplementary file 7 — Supplementary file7 (DOCX 20 kb) [file 249_2022_1593_MOESM7_ESM.docx]

Table S8. *S^2^_NH_*-values (11) for Trp (NE1-HE1) and Arg (NE-HE) side chains derived from relaxation measurements (Buck et al. 1995) and values calculated from the MD simulation in explicit water using the GROMOS 54A7 force field (*MD_water*), the SD simulations in vacuo using the GROMOS 54B7 force field without (*SD_nowater*) and with (*SD_implicit*) a SASA implicit-solvation term.

| Residue | Experimental value | *MD_water* | *SD_nowater* | *SD_implicit* |
| --- | --- | --- | --- | --- |
| Trp 28 | 0.90 | 0.88 | 0.80 | 0.83 |
| Trp 62 | 0.41 | 0.73 | 0.76 | 0.79 |
| Trp 63 | 0.88 | 0.83 | 0.87 | 0.85 |
| Trp 108 | 0.87 | 0.87 | 0.79 | 0.84 |
| Trp 111 | 0.88 | 0.83 | 0.79 | 0.88 |
| Trp 123 | 0.85 | 0.70 | 0.77 | 0.88 |
| Arg 61 | 0.28 | 0.22 | 0.35 | 0.30 |
| Arg 73 | 0.12 | 0.24 | 0.15 | 0.64 |
| Arg 112 | 0.31 | 0.28 | 0.50 | 0.40 |
| Arg 114 | 0.27 | 0.13 | 0.44 | 0.35 |
| Arg 125 | 0.05 | 0.12 | 0.38 | 0.34 |
