# Supplementary material for: Molecular dynamics simulation or structure refinement of proteins: are solvent molecules required? A case study using hen lysozyme
Source: Eur Biophys J. 2022 Mar 18;51(3):265–82. doi: 10.1007/s00249-022-01593-1 (PMC9035012; doi:10.1007/s00249-022-01593-1)
Supplement: Supplementary file 8 — Supplementary file8 (DOCX 14 kb) [file 249_2022_1593_MOESM8_ESM.docx]

Table S9. *S^2^_NH_*-values (17) for Asn (ND2-HD21, -HD22) and Gln (NE2-HE21, -HE22) side chains derived from relaxation measurements (Buck et al. 1995) and values calculated from the MD simulation in explicit water using the GROMOS 54A7 force field (*MD_water*), the SD simulations in vacuo using the GROMOS 54B7 force field without (*SD_nowater*) and with (*SD_implicit*) a SASA implicit-solvation term. The experimental values correspond to either HD/E21 or HD/E22. Stereo-specific assignments in the second column were taken from (Smith et al. 2021b) and is based on the best agreement with the values of the *MD_water* simulation (third column).

| Residue | Experimental value | *MD_water* | *SD_nowater* | *SD_implicit* |
| --- | --- | --- | --- | --- |
| Asn 19 HD21 | 0.43 | 0.49 | 0.77 | 0.76 |
| Asn 19 HD22 |  | 0.24 | 0.64 | 0.54 |
| Asn 27 HD21 |  | 0.86 | 0.77 | 0.90 |
| Asn 27 HD22 | 0.72 | 0.82 | 0.57 | 0.86 |
| Asn 37 HD21 | 0.51 | 0.37 | 0.88 | 0.73 |
| Asn 37 HD22 |  | 0.21 | 0.80 | 0.66 |
| Asn 39 HD21 | 0.74 | 0.80 | 0.80 | 0.40 |
| Asn 39 HD22 |  | 0.61 | 0.66 | 0.36 |
| Gln 41 HE21 |  | 0.31 | 0.67 | 0.61 |
| Gln 41 HE22 | 0.19 | 0.21 | 0.65 | 0.54 |
| Asn 44 HD21 |  | 0.75 | 0.71 | 0.75 |
| Asn 44 HD22 | 0.51 | 0.71 | 0.70 | 0.69 |
| Asn 46 HD21 |  | 0.85 | 0.85 | 0.79 |
| Asn 46 HD22 | 0.62 | 0.82 | 0.67 | 0.61 |
| Gln 57 HE21 | 0.82 | 0.79 | 0.80 | 0.87 |
| Gln 57 HE22 |  | 0.76 | 0.79 | 0.75 |
| Asn 59 HD21 |  | 0.92 | 0.76 | 0.77 |
| Asn 59 HD22 | 0.78 | 0.90 | 0.63 | 0.53 |
| Asn 65 HD21 |  | 0.76 | 0.86 | 0.77 |
| Asn 65 HD22 | 0.57 | 0.42 | 0.80 | 0.71 |
| Asn 74 HD21 | 0.74 | 0.66 | 0.88 | 0.66 |
| Asn 74 HD22 |  | 0.41 | 0.69 | 0.54 |
| Asn 77 HD21 |  | 0.54 | 0.69 | 0.75 |
| Asn 77 HD22 | 0.24 | 0.31 | 0.52 | 0.62 |
| Asn 93 HD21 | 0.59 | 0.53 | 0.55 | 0.87 |
| Asn 93 HD22 |  | 0.34 | 0.40 | 0.67 |
| Asn 103 HD21 |  | 0.72 | 0.79 | 0.70 |
| Asn 103 HD22 | 0.26 | 0.61 | 0.71 | 0.55 |
| Asn 106 HD21 | 0.58 | 0.68 | 0.77 | 0.87 |
| Asn 106 HD22 |  | 0.46 | 0.72 | 0.82 |
| Asn 113 HD21 | 0.47 | 0.40 | 0.83 | 0.75 |
| Asn 113 HD22 |  | 0.21 | 0.72 | 0.65 |
| Gln 121 HE21 | 0.36 | 0.34 | 0.78 | 0.63 |
| Gln 121 HE22 |  | 0.18 | 0.52 | 0.59 |
