# Supplementary material for: Molecular dynamics simulation or structure refinement of proteins: are solvent molecules required? A case study using hen lysozyme
Source: Eur Biophys J. 2022 Mar 18;51(3):265–82. doi: 10.1007/s00249-022-01593-1 (PMC9035012; doi:10.1007/s00249-022-01593-1)
Supplement: Supplementary file 9 — Supplementary file9 (DOCX 16 kb) [file 249_2022_1593_MOESM9_ESM.docx]

**Supporting Information**

Tables S1. List of 1630 NOE atom-atom distance upper bounds (in nm) derived from NMR experiments and *r*^-3^ averaged distances (in nm) for HEWL calculated from the *2VB1* X-ray structure, the MD simulation in explicit water using the GROMOS 54A7 force field (*MD_water*), the SD simulations in vacuo using the GROMOS 54B7 force field without (*SD_nowater*) and with (*SD_implicit*) a SASA implicit-solvation term.

Hen lysozyme NOE data set (data set from Schwalbe H, Grimshaw SB, Buck M, Spencer A, Boyd J, Dobson CM, Redfield C, Smith LJ (2001) A refined solution structure of hen lysozyme determined using residual dipolar coupling data. Protein Science 10:677-688).

Note that NOE number 297 has been corrected and is now 28 HZ3 – 56 HD rather than 28 HZ3 – 56 HG.

| NOE number | Residue and  atom 1 | | Residue and  atom 2 | | Upper bound | X-ray  *2VB1* | *MD_water* | *SD_nowater* | *SD_implicit* |
| --- | --- | --- | --- | --- | --- | --- | --- | --- | --- |
| 1 | 1 | HA | 2 | HN | 0.25 | 0.22 | 0.23 | 0.23 | 0.22 |
| 2 | 2 | HN | 2 | HA | 0.45 | 0.29 | 0.28 | 0.28 | 0.28 |
| 3 | 2 | HA | 2 | HB | 0.30 | 0.29 | 0.27 | 0.26 | 0.27 |
| 4 | 2 | HA | 2 | HG2@ | 0.55 | 0.29 | 0.30 | 0.32 | 0.31 |
| 5 | 2 | HA | 2 | HG1@ | 0.55 | 0.28 | 0.30 | 0.29 | 0.29 |
| 6 | 2 | HA | 3 | HN | 0.25 | 0.22 | 0.22 | 0.22 | 0.21 |
| 7 | 2 | HA | 40 | HN | 0.45 | 0.34 | 0.37 | 0.60 | 0.40 |
| 8 | 2 | HA | 39 | HA | 0.25 | 0.24 | 0.25 | 0.41 | 0.28 |
| 9 | 2 | HG2@ | 39 | HA | 0.60 | 0.37 | 0.42 | 0.68 | 0.47 |
| 10 | 2 | HG1@ | 39 | HA | 0.75 | 0.42 | 0.45 | 0.61 | 0.45 |
| 11 | 2 | HN | 2 | HB | 0.30 | 0.26 | 0.25 | 0.28 | 0.27 |
| 12 | 2 | HN | 2 | HG2@ | 0.60 | 0.34 | 0.32 | 0.29 | 0.30 |
| 13 | 2 | HN | 2 | HG1@ | 0.75 | 0.45 | 0.37 | 0.38 | 0.38 |
| 14 | 2 | HG2@ | 3 | HN | 0.75 | 0.51 | 0.44 | 0.48 | 0.50 |
| 15 | 2 | HG1@ | 3 | HN | 0.60 | 0.38 | 0.38 | 0.41 | 0.42 |
| 16 | 2 | HG2@ | 38 | HN | 0.75 | 0.78 | 0.72 | 0.82 | 0.79 |
| 17 | 2 | HG1@ | 38 | HN | 0.60 | 0.61 | 0.65 | 0.68 | 0.66 |
| 18 | 2 | HG2@ | 40 | HN | 0.75 | 0.50 | 0.56 | 0.86 | 0.60 |
| 19 | 2 | HB | 2 | HG2@ | 0.60 | 0.23 | 0.24 | 0.24 | 0.24 |
| 20 | 2 | HB | 2 | HG1@ | 0.60 | 0.24 | 0.24 | 0.24 | 0.24 |
| 21 | 2 | HN | 40 | HN | 0.55 | 0.49 | 0.51 | 0.77 | 0.52 |
| 22 | 2 | HG1@ | 40 | HN | 0.85 | 0.59 | 0.62 | 0.79 | 0.62 |
| 23 | 3 | HN | 3 | HA | 0.45 | 0.28 | 0.28 | 0.27 | 0.28 |
| 24 | 3 | HA | 3 | HB2 | 0.45 | 0.29 | 0.29 | 0.29 | 0.28 |
| 25 | 3 | HA | 3 | HB1 | 0.30 | 0.26 | 0.26 | 0.25 | 0.26 |
| 26 | 3 | HN | 3 | HB2 | 0.30 | 0.22 | 0.22 | 0.23 | 0.22 |
| 27 | 3 | HN | 3 | HB1 | 0.45 | 0.35 | 0.34 | 0.34 | 0.32 |
| 28 | 3 | HD@ | 3 | HE@ | 1.13 | 0.28 | 0.28 | 0.28 | 0.28 |
| 29 | 3 | HB2 | 3 | HD@ | 0.89 | 0.20 | 0.21 | 0.21 | 0.21 |
| 30 | 3 | HB1 | 3 | HD@ | 0.74 | 0.20 | 0.21 | 0.21 | 0.21 |
| 31 | 3 | HD@ | 8 | HN | 0.89 | 0.44 | 0.54 | 0.53 | 0.48 |
| 32 | 3 | HN | 3 | HD@ | 0.89 | 0.33 | 0.33 | 0.30 | 0.34 |
| 33 | 3 | HD@ | 4 | HN | 0.89 | 0.41 | 0.39 | 0.50 | 0.49 |
| 34 | 3 | HD@ | 40 | HN | 0.89 | 0.60 | 0.60 | 0.58 | 0.61 |
| 35 | 3 | HN | 39 | HA | 0.45 | 0.36 | 0.36 | 0.38 | 0.36 |
| 36 | 3 | HB1 | 8 | HN | 0.45 | 0.32 | 0.40 | 0.35 | 0.34 |
| 37 | 3 | HD@ | 7 | HB@ | 0.94 | 0.46 | 0.57 | 0.48 | 0.45 |
| 38 | 3 | HD@ | 8 | HD@ | 1.42 | 0.44 | 0.46 | 0.47 | 0.52 |
| 39 | 3 | HZ | 8 | HD@ | 0.98 | 0.61 | 0.64 | 0.67 | 0.75 |
| 40 | 3 | HB2 | 8 | HD@ | 0.98 | 0.40 | 0.42 | 0.44 | 0.45 |
| 41 | 3 | HD@ | 88 | HG2@ | 1.19 | 0.53 | 0.63 | 0.64 | 0.68 |
| 42 | 3 | HE@ | 88 | HG2@ | 1.04 | 0.35 | 0.46 | 0.49 | 0.56 |
| 43 | 3 | HZ | 88 | HG2@ | 0.55 | 0.33 | 0.43 | 0.45 | 0.54 |
| 44 | 3 | HE@ | 55 | HB | 0.74 | 0.68 | 0.65 | 0.68 | 0.67 |
| 45 | 3 | HD@ | 55 | HB | 0.74 | 0.65 | 0.60 | 0.68 | 0.58 |
| 46 | 3 | HE@ | 39 | HA | 1.19 | 0.79 | 0.73 | 0.47 | 0.80 |
| 47 | 3 | HN | 40 | HN | 0.55 | 0.42 | 0.46 | 0.59 | 0.43 |
| 48 | 3 | HB2 | 38 | HB@ | 0.50 | 0.30 | 0.33 | 0.45 | 0.45 |
| 49 | 3 | HB2 | 88 | HD@ | 0.85 | 0.78 | 0.72 | 0.62 | 0.67 |
| 50 | 3 | HN | 4 | HA@ | 0.75 | 0.59 | 0.57 | 0.51 | 0.50 |
| 51 | 3 | HE@ | 40 | HN | 0.99 | 0.68 | 0.66 | 0.50 | 0.71 |
| 52 | 3 | HE@ | 88 | HD@ | 1.29 | 0.53 | 0.49 | 0.51 | 0.45 |
| 53 | 3 | HD@ | 88 | HD@ | 1.29 | 0.68 | 0.62 | 0.56 | 0.55 |
| 54 | 4 | HN | 4 | HA@ | 0.65 | 0.24 | 0.24 | 0.23 | 0.23 |
| 55 | 4 | HA@ | 5 | HN | 0.65 | 0.26 | 0.25 | 0.27 | 0.26 |
| 56 | 4 | HN | 7 | HB@ | 0.50 | 0.33 | 0.35 | 0.37 | 0.41 |
| 57 | 5 | HN | 5 | HA | 0.30 | 0.28 | 0.27 | 0.21 | 0.21 |
| 58 | 6 | HA | 6 | HB2 | 0.45 | 0.28 | 0.29 | 0.28 | 0.28 |
| 59 | 6 | HA | 6 | HB1 | 0.30 | 0.24 | 0.24 | 0.25 | 0.25 |
| 60 | 6 | HA | 9 | HN | 0.45 | 0.32 | 0.36 | 0.33 | 0.34 |
| 61 | 6 | HA | 10 | HN | 0.45 | 0.41 | 0.39 | 0.40 | 0.38 |
| 62 | 6 | HN | 6 | HB2 | 0.30 | 0.24 | 0.24 | 0.22 | 0.24 |
| 63 | 6 | HN | 6 | HB1 | 0.45 | 0.36 | 0.34 | 0.34 | 0.32 |
| 64 | 6 | HB1 | 127 | HA | 0.45 | 0.33 | 0.48 | 0.54 | 0.56 |
| 65 | 6 | HA | 9 | HB@ | 0.55 | 0.32 | 0.35 | 0.34 | 0.32 |
| 66 | 6 | HN | 6 | HA | 0.45 | 0.28 | 0.27 | 0.27 | 0.27 |
| 67 | 6 | HB1 | 7 | HN | 0.45 | 0.36 | 0.34 | 0.36 | 0.36 |
| 68 | 6 | HN | 7 | HN | 0.30 | 0.26 | 0.26 | 0.28 | 0.28 |
| 69 | 7 | HN | 7 | HA | 0.30 | 0.28 | 0.26 | 0.27 | 0.27 |
| 70 | 7 | HN | 7 | HB@ | 0.50 | 0.29 | 0.25 | 0.27 | 0.27 |
| 71 | 7 | HA | 7 | HB@ | 0.50 | 0.26 | 0.25 | 0.26 | 0.26 |
| 72 | 7 | HA | 11 | HN | 0.45 | 0.42 | 0.39 | 0.42 | 0.41 |
| 73 | 7 | HA | 10 | HN | 0.45 | 0.35 | 0.37 | 0.35 | 0.37 |
| 74 | 7 | HA | 10 | HB@ | 0.75 | 0.39 | 0.37 | 0.36 | 0.38 |
| 75 | 7 | HB@ | 8 | HN | 0.65 | 0.30 | 0.30 | 0.30 | 0.29 |
| 76 | 7 | HN | 8 | HN | 0.30 | 0.27 | 0.30 | 0.28 | 0.28 |
| 77 | 8 | HN | 8 | HA | 0.30 | 0.28 | 0.27 | 0.27 | 0.27 |
| 78 | 8 | HN | 8 | HB1 | 0.30 | 0.25 | 0.26 | 0.28 | 0.26 |
| 79 | 8 | HN | 8 | HB2 | 0.30 | 0.26 | 0.23 | 0.22 | 0.22 |
| 80 | 8 | HA | 8 | HB1 | 0.45 | 0.29 | 0.29 | 0.28 | 0.29 |
| 81 | 8 | HA | 8 | HB2 | 0.45 | 0.24 | 0.25 | 0.26 | 0.25 |
| 82 | 8 | HA | 8 | HD@ | 0.78 | 0.35 | 0.35 | 0.35 | 0.34 |
| 83 | 8 | HN | 8 | HD@ | 0.98 | 0.49 | 0.46 | 0.44 | 0.46 |
| 84 | 8 | HA | 11 | HN | 0.45 | 0.34 | 0.36 | 0.34 | 0.36 |
| 85 | 8 | HA | 9 | HN | 0.45 | 0.35 | 0.35 | 0.35 | 0.35 |
| 86 | 8 | HN | 9 | HN | 0.30 | 0.27 | 0.29 | 0.29 | 0.29 |
| 87 | 8 | HN | 10 | HN | 0.45 | 0.43 | 0.45 | 0.44 | 0.46 |
| 88 | 8 | HA | 11 | HB@ | 0.60 | 0.36 | 0.35 | 0.35 | 0.36 |
| 89 | 8 | HB@ | 9 | HN | 0.65 | 0.32 | 0.31 | 0.30 | 0.31 |
| 90 | 8 | HD@ | 12 | HN | 0.98 | 0.53 | 0.59 | 0.62 | 0.58 |
| 91 | 8 | HD@ | 9 | HN | 0.98 | 0.52 | 0.51 | 0.52 | 0.51 |
| 92 | 8 | HD@ | 12 | HE@ | 0.93 | 0.40 | 0.46 | 0.50 | 0.54 |
| 93 | 8 | HD@ | 88 | HD@ | 1.28 | 0.50 | 0.51 | 0.65 | 0.56 |
| 94 | 8 | HD@ | 88 | HG2@ | 1.28 | 0.52 | 0.57 | 0.76 | 0.64 |
| 95 | 8 | HG | 8 | HD@ | 0.83 | 0.18 | 0.18 | 0.18 | 0.18 |
| 96 | 8 | HD@ | 38 | HE@ | 1.22 | 0.60 | 0.60 | 0.72 | 0.66 |
| 97 | 9 | HN | 9 | HA | 0.30 | 0.27 | 0.27 | 0.27 | 0.27 |
| 98 | 9 | HN | 9 | HB@ | 0.55 | 0.27 | 0.27 | 0.27 | 0.27 |
| 99 | 9 | HA | 9 | HB@ | 0.55 | 0.24 | 0.24 | 0.24 | 0.24 |
| 100 | 9 | HA | 12 | HN | 0.45 | 0.37 | 0.37 | 0.36 | 0.36 |
| 101 | 9 | HA | 10 | HN | 0.45 | 0.35 | 0.35 | 0.35 | 0.35 |
| 102 | 9 | HN | 10 | HN | 0.30 | 0.28 | 0.30 | 0.29 | 0.30 |
| 103 | 9 | HN | 11 | HN | 0.45 | 0.43 | 0.45 | 0.44 | 0.45 |
| 104 | 9 | HN | 129 | HD@ | 0.98 | 0.64 | 1.13 | 0.76 | 0.84 |
| 105 | 9 | HB@ | 124 | HG2@ | 0.85 | 0.36 | 0.37 | 0.66 | 0.36 |
| 106 | 10 | HN | 10 | HA | 0.30 | 0.28 | 0.27 | 0.27 | 0.27 |
| 107 | 10 | HN | 10 | HB@ | 0.55 | 0.28 | 0.27 | 0.27 | 0.27 |
| 108 | 10 | HA | 10 | HB@ | 0.55 | 0.24 | 0.24 | 0.24 | 0.24 |
| 109 | 10 | HA | 12 | HN | 0.45 | 0.45 | 0.46 | 0.46 | 0.45 |
| 110 | 10 | HA | 11 | HN | 0.45 | 0.35 | 0.35 | 0.35 | 0.35 |
| 111 | 10 | HN | 11 | HN | 0.30 | 0.27 | 0.29 | 0.29 | 0.29 |
| 112 | 10 | HN | 12 | HN | 0.45 | 0.43 | 0.44 | 0.46 | 0.44 |
| 113 | 10 | HN | 129 | HD@ | 0.83 | 0.50 | 1.08 | 0.67 | 0.62 |
| 114 | 10 | HA | 129 | HD@ | 0.78 | 0.42 | 1.10 | 0.64 | 0.50 |
| 115 | 11 | HN | 11 | HA | 0.30 | 0.28 | 0.27 | 0.27 | 0.27 |
| 116 | 11 | HN | 11 | HB@ | 0.55 | 0.27 | 0.27 | 0.27 | 0.27 |
| 117 | 11 | HA | 11 | HB@ | 0.55 | 0.24 | 0.24 | 0.24 | 0.24 |
| 118 | 11 | HA | 12 | HN | 0.30 | 0.35 | 0.35 | 0.35 | 0.35 |
| 119 | 11 | HN | 12 | HN | 0.30 | 0.27 | 0.29 | 0.29 | 0.29 |
| 120 | 11 | HN | 13 | HN | 0.45 | 0.41 | 0.44 | 0.44 | 0.44 |
| 121 | 11 | HB@ | 12 | HN | 0.55 | 0.33 | 0.32 | 0.32 | 0.33 |
| 122 | 11 | HB@ | 13 | HN | 0.75 | 0.54 | 0.56 | 0.56 | 0.56 |
| 123 | 11 | HA | 14 | HN | 0.45 | 0.34 | 0.36 | 0.36 | 0.37 |
| 124 | 11 | HB@ | 88 | HD@ | 0.85 | 0.36 | 0.40 | 0.46 | 0.38 |
| 125 | 11 | HN | 88 | HD@ | 0.85 | 0.55 | 0.62 | 0.66 | 0.61 |
| 126 | 12 | HN | 12 | HA | 0.30 | 0.28 | 0.27 | 0.27 | 0.27 |
| 127 | 12 | HA | 13 | HN | 0.45 | 0.35 | 0.35 | 0.35 | 0.35 |
| 128 | 12 | HN | 13 | HN | 0.30 | 0.27 | 0.29 | 0.29 | 0.29 |
| 129 | 12 | HA | 15 | HB2 | 0.45 | 0.30 | 0.31 | 0.35 | 0.33 |
| 130 | 12 | HN | 88 | HD@ | 0.75 | 0.38 | 0.50 | 0.69 | 0.54 |
| 131 | 12 | HA | 88 | HD@ | 0.60 | 0.32 | 0.47 | 0.72 | 0.54 |
| 132 | 12 | HE@ | 88 | HD@ | 0.90 | 0.51 | 0.58 | 0.87 | 0.76 |
| 133 | 12 | HE@ | 17 | HD@ | 0.98 | 0.54 | 0.54 | 0.68 | 0.47 |
| 134 | 12 | HA | 17 | HD@ | 0.98 | 0.51 | 0.56 | 0.62 | 0.53 |
| 135 | 12 | HA | 17 | HG | 0.45 | 0.41 | 0.55 | 0.58 | 0.61 |
| 136 | 12 | HN | 17 | HD@ | 1.08 | 0.74 | 0.75 | 0.84 | 0.73 |
| 137 | 12 | HN | 14 | HN | 0.55 | 0.43 | 0.44 | 0.45 | 0.45 |
| 138 | 13 | HN | 13 | HA | 0.30 | 0.28 | 0.27 | 0.26 | 0.27 |
| 139 | 13 | HN | 13 | HB@ | 0.50 | 0.24 | 0.24 | 0.26 | 0.23 |
| 140 | 13 | HA | 13 | HB@ | 0.50 | 0.25 | 0.26 | 0.26 | 0.25 |
| 141 | 13 | HN | 14 | HN | 0.30 | 0.27 | 0.29 | 0.30 | 0.29 |
| 142 | 13 | HA | 14 | HN | 0.45 | 0.35 | 0.35 | 0.35 | 0.35 |
| 143 | 13 | HN | 129 | HD@ | 0.98 | 0.60 | 1.46 | 0.73 | 0.71 |
| 144 | 13 | HB@ | 14 | HN | 0.50 | 0.32 | 0.29 | 0.31 | 0.30 |
| 145 | 13 | HN | 25 | HD@ | 0.83 | 0.38 | 0.40 | 0.49 | 0.41 |
| 146 | 13 | HN | 88 | HD@ | 0.85 | 0.60 | 0.76 | 0.90 | 0.80 |
| 147 | 14 | HN | 14 | HA | 0.45 | 0.27 | 0.27 | 0.26 | 0.26 |
| 148 | 14 | HN | 14 | HB@ | 0.50 | 0.30 | 0.28 | 0.27 | 0.27 |
| 149 | 14 | HN | 15 | HN | 0.30 | 0.27 | 0.29 | 0.29 | 0.29 |
| 150 | 15 | HN | 15 | HA | 0.45 | 0.28 | 0.28 | 0.28 | 0.27 |
| 151 | 15 | HN | 15 | HB2 | 0.30 | 0.25 | 0.23 | 0.23 | 0.23 |
| 152 | 15 | HN | 15 | HB1 | 0.45 | 0.36 | 0.35 | 0.29 | 0.34 |
| 153 | 15 | HA | 15 | HB2 | 0.30 | 0.29 | 0.29 | 0.27 | 0.29 |
| 154 | 15 | HN | 16 | HN | 0.45 | 0.27 | 0.29 | 0.30 | 0.30 |
| 155 | 15 | HB2 | 92 | HG1@ | 0.60 | 0.34 | 0.39 | 0.40 | 0.36 |
| 156 | 15 | HB2 | 92 | HG2@ | 0.75 | 0.35 | 0.38 | 0.48 | 0.48 |
| 157 | 15 | HE1 | 88 | HG1@ | 0.65 | 0.41 | 0.70 | 0.66 | 0.51 |
| 158 | 15 | HE1 | 88 | HD@ | 0.60 | 0.55 | 0.72 | 0.76 | 0.48 |
| 159 | 15 | HE1 | 92 | HG2@ | 0.75 | 0.66 | 0.75 | 0.62 | 0.78 |
| 160 | 15 | HE1 | 92 | HG1@ | 0.75 | 0.77 | 0.78 | 0.59 | 0.73 |
| 161 | 16 | HN | 16 | HA@ | 0.65 | 0.24 | 0.24 | 0.23 | 0.24 |
| 162 | 16 | HN | 17 | HD@ | 1.28 | 0.67 | 0.71 | 0.74 | 0.70 |
| 163 | 16 | HN | 17 | HN | 0.45 | 0.26 | 0.28 | 0.31 | 0.28 |
| 164 | 17 | HN | 17 | HA | 0.30 | 0.28 | 0.28 | 0.28 | 0.28 |
| 165 | 17 | HN | 17 | HB1 | 0.45 | 0.36 | 0.28 | 0.30 | 0.29 |
| 166 | 17 | HN | 17 | HB2 | 0.45 | 0.24 | 0.25 | 0.23 | 0.23 |
| 167 | 17 | HA | 17 | HB1 | 0.45 | 0.26 | 0.28 | 0.27 | 0.27 |
| 168 | 17 | HA | 17 | HB2 | 0.45 | 0.29 | 0.26 | 0.27 | 0.26 |
| 169 | 17 | HA | 17 | HD@ | 0.98 | 0.35 | 0.34 | 0.34 | 0.35 |
| 170 | 17 | HN | 17 | HG | 0.25 | 0.28 | 0.36 | 0.31 | 0.38 |
| 171 | 17 | HG | 17 | HD@ | 0.83 | 0.18 | 0.18 | 0.18 | 0.18 |
| 172 | 17 | HD@ | 28 | HZ2 | 0.98 | 0.50 | 0.47 | 0.48 | 0.48 |
| 173 | 17 | HD@ | 28 | HE3 | 0.98 | 0.44 | 0.66 | 0.63 | 0.69 |
| 174 | 17 | HD@ | 28 | HH2 | 0.98 | 0.52 | 0.64 | 0.40 | 0.63 |
| 175 | 17 | HD@ | 18 | HN | 0.98 | 0.59 | 0.51 | 0.56 | 0.53 |
| 176 | 17 | HD@ | 28 | HE1 | 0.98 | 0.50 | 0.37 | 0.66 | 0.43 |
| 177 | 17 | HA | 18 | HN | 0.30 | 0.33 | 0.24 | 0.23 | 0.23 |
| 178 | 17 | HN | 18 | HN | 0.30 | 0.26 | 0.30 | 0.37 | 0.38 |
| 179 | 17 | HG | 18 | HN | 0.45 | 0.49 | 0.40 | 0.46 | 0.39 |
| 180 | 17 | HD@ | 28 | HZ3 | 0.98 | 0.50 | 0.72 | 0.49 | 0.72 |
| 181 | 17 | HD@ | 92 | HG1@ | 1.13 | 0.37 | 0.46 | 0.50 | 0.46 |
| 182 | 17 | HD@ | 92 | HG2@ | 1.13 | 0.46 | 0.52 | 0.70 | 0.43 |
| 183 | 17 | HB@ | 28 | HE3 | 0.65 | 0.55 | 0.86 | 0.63 | 0.83 |
| 184 | 17 | HD@ | 28 | HD1 | 0.98 | 0.52 | 0.41 | 0.82 | 0.48 |
| 185 | 17 | HB@ | 17 | HD@ | 1.18 | 0.26 | 0.26 | 0.26 | 0.26 |
| 186 | 17 | HB@ | 17 | HG | 0.45 | 0.25 | 0.25 | 0.25 | 0.25 |
| 187 | 17 | HD@ | 19 | HN | 1.28 | 0.75 | 0.79 | 0.52 | 0.59 |
| 188 | 17 | HD@ | 20 | HD@ | 1.42 | 0.57 | 0.74 | 0.52 | 0.62 |
| 189 | 17 | HD@ | 20 | HB2 | 0.83 | 0.51 | 0.70 | 0.41 | 0.50 |
| 190 | 17 | HB@ | 28 | HZ3 | 0.75 | 0.67 | 0.95 | 0.48 | 0.90 |
| 191 | 17 | HD@ | 92 | HB | 1.08 | 0.55 | 0.54 | 0.69 | 0.53 |
| 192 | 17 | HD@ | 88 | HD@ | 1.58 | 0.69 | 0.88 | 1.27 | 0.99 |
| 193 | 17 | HD@ | 96 | HN | 1.08 | 0.53 | 0.48 | 0.59 | 0.53 |
| 194 | 17 | HA | 28 | HE1 | 0.55 | 0.42 | 0.56 | 0.87 | 0.70 |
| 195 | 17 | HD@ | 20 | HE@ | 1.72 | 0.73 | 0.76 | 0.65 | 0.78 |
| 196 | 17 | HD@ | 55 | HG2@ | 1.58 | 0.89 | 1.15 | 1.16 | 1.24 |
| 197 | 18 | HN | 18 | HA | 0.45 | 0.28 | 0.27 | 0.27 | 0.27 |
| 198 | 18 | HN | 18 | HB2 | 0.30 | 0.26 | 0.25 | 0.23 | 0.24 |
| 199 | 18 | HN | 18 | HB1 | 0.30 | 0.26 | 0.27 | 0.27 | 0.27 |
| 200 | 18 | HA | 18 | HB2 | 0.30 | 0.24 | 0.26 | 0.26 | 0.25 |
| 201 | 18 | HA | 18 | HB1 | 0.45 | 0.29 | 0.26 | 0.28 | 0.26 |
| 202 | 18 | HA | 19 | HN | 0.25 | 0.22 | 0.24 | 0.30 | 0.25 |
| 203 | 18 | HN | 25 | HD@ | 0.98 | 0.48 | 0.60 | 0.63 | 0.67 |
| 204 | 18 | HN | 25 | HN | 0.55 | 0.55 | 0.63 | 0.83 | 0.76 |
| 205 | 18 | HA | 28 | HE1 | 0.55 | 0.44 | 0.60 | 0.95 | 0.89 |
| 206 | 19 | HN | 19 | HA | 0.30 | 0.22 | 0.24 | 0.25 | 0.25 |
| 207 | 19 | HN | 19 | HB@ | 0.65 | 0.34 | 0.28 | 0.33 | 0.27 |
| 208 | 19 | HA | 19 | HB@ | 0.50 | 0.26 | 0.26 | 0.26 | 0.26 |
| 209 | 19 | HN | 20 | HN | 0.30 | 0.26 | 0.37 | 0.42 | 0.38 |
| 210 | 19 | HN | 24 | HN | 0.75 | 0.43 | 0.61 | 0.89 | 0.80 |
| 211 | 19 | HN | 28 | HE1 | 0.55 | 0.38 | 0.70 | 0.75 | 0.74 |
| 212 | 20 | HN | 20 | HB2 | 0.30 | 0.26 | 0.25 | 0.30 | 0.30 |
| 213 | 20 | HN | 20 | HB1 | 0.30 | 0.26 | 0.30 | 0.28 | 0.27 |
| 214 | 20 | HA | 20 | HB2 | 0.25 | 0.24 | 0.26 | 0.25 | 0.24 |
| 215 | 20 | HA | 20 | HB1 | 0.45 | 0.29 | 0.26 | 0.29 | 0.27 |
| 216 | 20 | HB2 | 20 | HD@ | 0.74 | 0.21 | 0.21 | 0.21 | 0.21 |
| 217 | 20 | HB1 | 20 | HD@ | 0.74 | 0.21 | 0.21 | 0.21 | 0.21 |
| 218 | 20 | HA | 20 | HD@ | 0.89 | 0.27 | 0.28 | 0.27 | 0.29 |
| 219 | 20 | HN | 20 | HA | 0.45 | 0.28 | 0.28 | 0.28 | 0.28 |
| 220 | 20 | HD@ | 21 | HN | 0.89 | 0.35 | 0.38 | 0.27 | 0.31 |
| 221 | 20 | HN | 28 | HE1 | 0.45 | 0.31 | 0.60 | 0.50 | 0.58 |
| 222 | 20 | HA | 21 | HN | 0.25 | 0.21 | 0.22 | 0.23 | 0.23 |
| 223 | 20 | HE@ | 96 | HB@ | 1.09 | 0.37 | 0.51 | 0.57 | 0.59 |
| 224 | 20 | HN | 21 | HN | 0.55 | 0.45 | 0.43 | 0.44 | 0.44 |
| 225 | 20 | HE@ | 21 | HN | 0.99 | 0.47 | 0.56 | 0.39 | 0.43 |
| 226 | 21 | HN | 21 | HA | 0.25 | 0.22 | 0.22 | 0.27 | 0.26 |
| 227 | 21 | HA | 21 | HB@ | 0.50 | 0.25 | 0.26 | 0.24 | 0.25 |
| 228 | 21 | HA | 22 | HN | 0.45 | 0.28 | 0.24 | 0.21 | 0.22 |
| 229 | 21 | HN | 22 | HN | 0.30 | 0.28 | 0.32 | 0.44 | 0.39 |
| 230 | 21 | HN | 22 | HA@ | 0.75 | 0.51 | 0.54 | 0.59 | 0.57 |
| 231 | 21 | HA | 23 | HN | 0.75 | 0.46 | 0.42 | 0.45 | 0.45 |
| 232 | 21 | HN | 23 | HN | 0.75 | 0.44 | 0.45 | 0.49 | 0.50 |
| 233 | 22 | HN | 22 | HA@ | 0.65 | 0.24 | 0.23 | 0.24 | 0.24 |
| 234 | 23 | HN | 23 | HA | 0.45 | 0.29 | 0.28 | 0.28 | 0.28 |
| 235 | 23 | HN | 23 | HB2 | 0.45 | 0.25 | 0.25 | 0.24 | 0.24 |
| 236 | 23 | HN | 23 | HB1 | 0.45 | 0.36 | 0.36 | 0.35 | 0.35 |
| 237 | 23 | HA | 23 | HB1 | 0.30 | 0.25 | 0.25 | 0.24 | 0.24 |
| 238 | 23 | HA | 23 | HD@ | 0.89 | 0.26 | 0.28 | 0.28 | 0.27 |
| 239 | 23 | HE@ | 111 | HH2 | 0.89 | 0.51 | 0.48 | 0.71 | 0.55 |
| 240 | 23 | HB2 | 23 | HD@ | 0.74 | 0.21 | 0.21 | 0.21 | 0.21 |
| 241 | 23 | HB1 | 23 | HD@ | 0.74 | 0.21 | 0.21 | 0.21 | 0.21 |
| 242 | 23 | HB1 | 28 | HE1 | 0.45 | 0.30 | 0.39 | 0.31 | 0.32 |
| 243 | 23 | HD@ | 24 | HN | 0.89 | 0.46 | 0.43 | 0.49 | 0.49 |
| 244 | 23 | HD@ | 111 | HZ2 | 0.89 | 0.57 | 0.56 | 0.62 | 0.49 |
| 245 | 23 | HD@ | 105 | HB@ | 1.09 | 0.47 | 0.57 | 0.42 | 0.56 |
| 246 | 23 | HE@ | 111 | HZ2 | 0.74 | 0.53 | 0.39 | 0.58 | 0.64 |
| 247 | 23 | HE@ | 105 | HB@ | 1.09 | 0.41 | 0.43 | 0.36 | 0.49 |
| 248 | 23 | HD@ | 28 | HE1 | 0.89 | 0.44 | 0.57 | 0.48 | 0.53 |
| 249 | 23 | HN | 28 | HZ2 | 0.30 | 0.47 | 0.72 | 0.63 | 0.66 |
| 250 | 23 | HE@ | 111 | HE1 | 0.99 | 0.67 | 0.45 | 0.50 | 0.73 |
| 251 | 23 | HE@ | 99 | HG@ | 1.27 | 0.53 | 0.56 | 0.62 | 0.59 |
| 252 | 23 | HN | 28 | HE1 | 0.55 | 0.38 | 0.66 | 0.50 | 0.55 |
| 253 | 23 | HN | 24 | HN | 0.55 | 0.43 | 0.44 | 0.39 | 0.36 |
| 254 | 24 | HN | 24 | HA | 0.30 | 0.27 | 0.28 | 0.26 | 0.28 |
| 255 | 25 | HN | 25 | HA | 0.30 | 0.27 | 0.25 | 0.27 | 0.26 |
| 256 | 25 | HN | 25 | HB@ | 0.50 | 0.23 | 0.27 | 0.24 | 0.27 |
| 257 | 25 | HN | 26 | HN | 0.45 | 0.29 | 0.30 | 0.29 | 0.30 |
| 258 | 25 | HN | 25 | HG | 0.30 | 0.45 | 0.26 | 0.35 | 0.25 |
| 259 | 25 | HN | 25 | HD@ | 0.98 | 0.47 | 0.40 | 0.44 | 0.39 |
| 260 | 25 | HA | 25 | HB@ | 0.65 | 0.26 | 0.26 | 0.26 | 0.26 |
| 261 | 25 | HB@ | 25 | HD@ | 1.03 | 0.26 | 0.26 | 0.26 | 0.26 |
| 262 | 25 | HB@ | 26 | HN | 0.50 | 0.32 | 0.32 | 0.32 | 0.29 |
| 263 | 25 | HA | 26 | HN | 0.45 | 0.35 | 0.34 | 0.34 | 0.35 |
| 264 | 25 | HD@ | 28 | HD1 | 0.83 | 0.54 | 0.53 | 0.66 | 0.59 |
| 265 | 25 | HN | 28 | HE1 | 0.55 | 0.56 | 0.49 | 0.53 | 0.53 |
| 266 | 25 | HA | 28 | HE1 | 0.55 | 0.47 | 0.43 | 0.44 | 0.47 |
| 267 | 26 | HN | 26 | HA@ | 0.65 | 0.24 | 0.23 | 0.23 | 0.23 |
| 268 | 26 | HN | 27 | HN | 0.30 | 0.28 | 0.30 | 0.29 | 0.28 |
| 269 | 26 | HN | 29 | HG@ | 0.98 | 0.66 | 0.63 | 0.65 | 0.64 |
| 270 | 26 | HN | 120 | HG@ | 0.98 | 0.60 | 0.51 | 0.54 | 0.38 |
| 271 | 26 | HN | 30 | HN | 0.75 | 0.59 | 0.60 | 0.61 | 0.60 |
| 272 | 26 | HN | 29 | HN | 0.55 | 0.49 | 0.50 | 0.50 | 0.49 |
| 273 | 26 | HN | 28 | HN | 0.55 | 0.43 | 0.45 | 0.44 | 0.45 |
| 274 | 27 | HN | 27 | HA | 0.45 | 0.27 | 0.27 | 0.27 | 0.27 |
| 275 | 27 | HN | 27 | HB2 | 0.30 | 0.23 | 0.33 | 0.23 | 0.25 |
| 276 | 27 | HN | 27 | HB1 | 0.45 | 0.35 | 0.26 | 0.29 | 0.35 |
| 277 | 27 | HA | 27 | HB2 | 0.30 | 0.29 | 0.25 | 0.26 | 0.29 |
| 278 | 27 | HA | 27 | HB1 | 0.30 | 0.26 | 0.23 | 0.27 | 0.24 |
| 279 | 27 | HB2 | 28 | HN | 0.30 | 0.28 | 0.33 | 0.33 | 0.27 |
| 280 | 27 | HA | 30 | HN | 0.45 | 0.37 | 0.37 | 0.35 | 0.34 |
| 281 | 27 | HN | 28 | HN | 0.30 | 0.28 | 0.29 | 0.27 | 0.28 |
| 282 | 27 | HA | 30 | HB2 | 0.45 | 0.38 | 0.37 | 0.35 | 0.32 |
| 283 | 27 | HN | 120 | HG@ | 0.98 | 0.43 | 0.75 | 0.44 | 0.52 |
| 284 | 27 | HA | 111 | HE1 | 0.30 | 0.31 | 0.52 | 0.51 | 0.39 |
| 285 | 27 | HA | 123 | HE1 | 0.75 | 0.74 | 0.54 | 0.76 | 0.44 |
| 286 | 27 | HB1 | 111 | HE1 | 0.30 | 0.26 | 0.37 | 0.34 | 0.33 |
| 287 | 27 | HN | 28 | HE1 | 0.75 | 0.61 | 0.62 | 0.59 | 0.59 |
| 288 | 28 | HN | 28 | HA | 0.30 | 0.28 | 0.27 | 0.27 | 0.27 |
| 289 | 28 | HA | 28 | HB@ | 0.65 | 0.26 | 0.25 | 0.26 | 0.26 |
| 290 | 28 | HB@ | 29 | HN | 0.65 | 0.31 | 0.30 | 0.28 | 0.29 |
| 291 | 28 | HN | 29 | HN | 0.30 | 0.27 | 0.29 | 0.28 | 0.29 |
| 292 | 28 | HH2 | 28 | HZ2 | 0.45 | 0.25 | 0.25 | 0.25 | 0.25 |
| 293 | 28 | HE3 | 28 | HZ3 | 0.25 | 0.25 | 0.25 | 0.25 | 0.25 |
| 294 | 28 | HZ3 | 28 | HH2 | 0.30 | 0.25 | 0.25 | 0.25 | 0.25 |
| 295 | 28 | HE3 | 28 | HA | 0.30 | 0.27 | 0.25 | 0.43 | 0.31 |
| 296 | 28 | HD1 | 28 | HB@ | 0.65 | 0.31 | 0.29 | 0.34 | 0.31 |
| 297 | 28 | HZ3 | 56 | HD@ | 0.59 | 0.41 | 0.44 | 0.52 | 0.50 |
| 298 | 28 | HE1 | 28 | HZ2 | 0.30 | 0.29 | 0.28 | 0.29 | 0.28 |
| 299 | 28 | HE1 | 28 | HN | 0.45 | 0.47 | 0.47 | 0.46 | 0.44 |
| 300 | 28 | HE1 | 28 | HD1 | 0.25 | 0.26 | 0.25 | 0.25 | 0.25 |
| 301 | 28 | HE3 | 56 | HD@ | 0.83 | 0.34 | 0.32 | 0.41 | 0.44 |
| 302 | 28 | HE3 | 32 | HB@ | 0.75 | 0.65 | 0.66 | 0.65 | 0.65 |
| 303 | 28 | HH2 | 95 | HB@ | 0.75 | 0.41 | 0.42 | 0.39 | 0.32 |
| 304 | 28 | HE3 | 95 | HB@ | 0.75 | 0.46 | 0.52 | 0.45 | 0.52 |
| 305 | 28 | HZ3 | 95 | HB@ | 0.60 | 0.32 | 0.47 | 0.32 | 0.36 |
| 306 | 28 | HZ3 | 108 | HE1 | 0.75 | 0.54 | 0.42 | 1.03 | 0.67 |
| 307 | 28 | HZ3 | 98 | HG2@ | 1.05 | 0.66 | 0.49 | 0.89 | 0.75 |
| 308 | 28 | HH2 | 99 | HG@ | 0.83 | 0.37 | 0.39 | 0.50 | 0.42 |
| 309 | 28 | HH2 | 99 | HB | 0.30 | 0.38 | 0.53 | 0.59 | 0.43 |
| 310 | 28 | HZ2 | 99 | HG@ | 0.83 | 0.41 | 0.28 | 0.39 | 0.33 |
| 311 | 28 | HZ3 | 99 | HN | 0.55 | 0.61 | 0.62 | 0.71 | 0.67 |
| 312 | 28 | HE3 | 88 | HD@ | 1.05 | 0.87 | 1.04 | 1.03 | 1.02 |
| 313 | 29 | HN | 29 | HA | 0.45 | 0.28 | 0.27 | 0.27 | 0.27 |
| 314 | 29 | HA | 29 | HB | 0.45 | 0.29 | 0.27 | 0.28 | 0.28 |
| 315 | 29 | HN | 29 | HG1@ | 0.75 | 0.44 | 0.39 | 0.42 | 0.43 |
| 316 | 29 | HN | 29 | HG2@ | 0.60 | 0.30 | 0.29 | 0.28 | 0.29 |
| 317 | 29 | HA | 30 | HN | 0.45 | 0.35 | 0.35 | 0.35 | 0.35 |
| 318 | 29 | HN | 30 | HN | 0.30 | 0.27 | 0.28 | 0.29 | 0.30 |
| 319 | 29 | HB | 30 | HN | 0.30 | 0.27 | 0.28 | 0.26 | 0.27 |
| 320 | 29 | HG@ | 30 | HN | 0.83 | 0.42 | 0.41 | 0.41 | 0.42 |
| 321 | 30 | HN | 30 | HA | 0.30 | 0.28 | 0.27 | 0.27 | 0.26 |
| 322 | 30 | HN | 30 | HB2 | 0.30 | 0.23 | 0.23 | 0.22 | 0.22 |
| 323 | 30 | HN | 30 | HB1 | 0.30 | 0.24 | 0.23 | 0.24 | 0.23 |
| 324 | 30 | HB2 | 31 | HN | 0.45 | 0.38 | 0.38 | 0.38 | 0.38 |
| 325 | 30 | HB1 | 31 | HN | 0.30 | 0.26 | 0.25 | 0.25 | 0.25 |
| 326 | 30 | HN | 120 | HG@ | 0.83 | 0.60 | 0.82 | 0.57 | 0.89 |
| 327 | 30 | HB1 | 123 | HE1 | 0.55 | 0.52 | 0.49 | 0.65 | 0.50 |
| 328 | 31 | HN | 31 | HA | 0.30 | 0.27 | 0.27 | 0.27 | 0.27 |
| 329 | 31 | HA | 31 | HB@ | 0.55 | 0.24 | 0.24 | 0.24 | 0.24 |
| 330 | 31 | HA | 32 | HN | 0.45 | 0.35 | 0.35 | 0.35 | 0.35 |
| 331 | 31 | HA | 34 | HN | 0.45 | 0.37 | 0.36 | 0.36 | 0.38 |
| 332 | 31 | HN | 32 | HN | 0.45 | 0.28 | 0.29 | 0.29 | 0.28 |
| 333 | 32 | HN | 32 | HA | 0.30 | 0.28 | 0.27 | 0.27 | 0.27 |
| 334 | 32 | HN | 32 | HB@ | 0.55 | 0.27 | 0.26 | 0.27 | 0.27 |
| 335 | 32 | HA | 32 | HB@ | 0.55 | 0.24 | 0.24 | 0.24 | 0.24 |
| 336 | 32 | HA | 33 | HN | 0.45 | 0.35 | 0.35 | 0.35 | 0.35 |
| 337 | 32 | HA | 35 | HB@ | 0.50 | 0.35 | 0.44 | 0.51 | 0.43 |
| 338 | 32 | HN | 33 | HN | 0.45 | 0.27 | 0.30 | 0.30 | 0.30 |
| 339 | 32 | HB@ | 33 | HN | 0.60 | 0.34 | 0.33 | 0.33 | 0.34 |
| 340 | 32 | HB@ | 38 | HE@ | 1.04 | 0.54 | 0.44 | 0.63 | 0.50 |
| 341 | 32 | HB@ | 35 | HN | 0.75 | 0.54 | 0.59 | 0.60 | 0.59 |
| 342 | 32 | HB@ | 38 | HD@ | 0.99 | 0.51 | 0.45 | 0.60 | 0.51 |
| 343 | 32 | HN | 56 | HD@ | 0.83 | 0.48 | 0.54 | 0.56 | 0.70 |
| 344 | 33 | HN | 33 | HA | 0.30 | 0.27 | 0.27 | 0.27 | 0.27 |
| 345 | 33 | HA | 34 | HN | 0.45 | 0.35 | 0.35 | 0.35 | 0.35 |
| 346 | 33 | HN | 34 | HN | 0.30 | 0.28 | 0.30 | 0.30 | 0.30 |
| 347 | 33 | HA | 38 | HE@ | 0.89 | 0.35 | 0.39 | 0.40 | 0.36 |
| 348 | 33 | HN | 38 | HE@ | 0.89 | 0.42 | 0.39 | 0.50 | 0.40 |
| 349 | 33 | HN | 38 | HD@ | 0.89 | 0.51 | 0.50 | 0.59 | 0.52 |
| 350 | 34 | HN | 34 | HA | 0.45 | 0.28 | 0.27 | 0.27 | 0.27 |
| 351 | 34 | HN | 34 | HB2 | 0.30 | 0.24 | 0.23 | 0.23 | 0.23 |
| 352 | 34 | HN | 34 | HB1 | 0.45 | 0.36 | 0.33 | 0.35 | 0.34 |
| 353 | 34 | HA | 34 | HB2 | 0.25 | 0.29 | 0.28 | 0.29 | 0.29 |
| 354 | 34 | HA | 34 | HB1 | 0.30 | 0.25 | 0.26 | 0.26 | 0.25 |
| 355 | 34 | HN | 35 | HN | 0.30 | 0.24 | 0.28 | 0.29 | 0.28 |
| 356 | 34 | HD@ | 34 | HE@ | 1.18 | 0.28 | 0.28 | 0.28 | 0.28 |
| 357 | 34 | HB2 | 34 | HD@ | 0.74 | 0.21 | 0.21 | 0.21 | 0.21 |
| 358 | 34 | HB1 | 34 | HD@ | 0.74 | 0.21 | 0.21 | 0.21 | 0.21 |
| 359 | 34 | HA | 34 | HD@ | 0.74 | 0.26 | 0.27 | 0.27 | 0.27 |
| 360 | 34 | HE@ | 114 | HB@ | 1.09 | 0.43 | 0.45 | 0.39 | 0.54 |
| 361 | 34 | HE@ | 123 | HZ2 | 0.89 | 0.32 | 0.59 | 0.56 | 0.64 |
| 362 | 35 | HN | 35 | HA | 0.45 | 0.28 | 0.28 | 0.28 | 0.28 |
| 363 | 35 | HN | 35 | HB@ | 0.45 | 0.29 | 0.29 | 0.28 | 0.28 |
| 364 | 35 | HA | 35 | HB@ | 0.65 | 0.26 | 0.26 | 0.26 | 0.26 |
| 365 | 35 | HN | 36 | HN | 0.30 | 0.26 | 0.28 | 0.26 | 0.29 |
| 366 | 35 | HB@ | 38 | HE@ | 1.09 | 0.96 | 1.00 | 1.12 | 1.01 |
| 367 | 35 | HN | 38 | HE@ | 0.89 | 0.78 | 0.81 | 0.88 | 0.80 |
| 368 | 36 | HN | 36 | HA | 0.45 | 0.28 | 0.27 | 0.28 | 0.27 |
| 369 | 36 | HN | 36 | HB1 | 0.45 | 0.31 | 0.28 | 0.33 | 0.29 |
| 370 | 36 | HN | 36 | HB2 | 0.45 | 0.38 | 0.34 | 0.28 | 0.31 |
| 371 | 36 | HA | 37 | HN | 0.45 | 0.32 | 0.32 | 0.23 | 0.32 |
| 372 | 36 | HB1 | 39 | HN | 0.45 | 0.40 | 0.42 | 0.52 | 0.46 |
| 373 | 36 | HB2 | 39 | HN | 0.45 | 0.27 | 0.29 | 0.51 | 0.37 |
| 374 | 36 | HN | 37 | HN | 0.30 | 0.21 | 0.25 | 0.36 | 0.27 |
| 375 | 36 | HB1 | 55 | HA | 0.30 | 0.25 | 0.36 | 0.38 | 0.33 |
| 376 | 36 | HB2 | 55 | HA | 0.30 | 0.27 | 0.35 | 0.35 | 0.31 |
| 377 | 36 | HA | 42 | HB@ | 0.75 | 0.35 | 0.35 | 0.36 | 0.34 |
| 378 | 37 | HN | 37 | HA | 0.25 | 0.22 | 0.21 | 0.26 | 0.21 |
| 379 | 37 | HA | 37 | HB@ | 0.50 | 0.26 | 0.25 | 0.26 | 0.25 |
| 380 | 37 | HA | 38 | HN | 0.45 | 0.27 | 0.26 | 0.21 | 0.26 |
| 381 | 37 | HN | 38 | HN | 0.45 | 0.27 | 0.27 | 0.40 | 0.27 |
| 382 | 37 | HA | 39 | HN | 0.45 | 0.44 | 0.45 | 0.42 | 0.43 |
| 383 | 38 | HN | 38 | HA | 0.25 | 0.21 | 0.20 | 0.20 | 0.20 |
| 384 | 38 | HN | 38 | HB@ | 0.65 | 0.37 | 0.36 | 0.36 | 0.36 |
| 385 | 38 | HA | 38 | HB@ | 0.45 | 0.26 | 0.26 | 0.26 | 0.26 |
| 386 | 38 | HB@ | 38 | HD@ | 1.09 | 0.19 | 0.19 | 0.19 | 0.19 |
| 387 | 39 | HN | 39 | HA | 0.30 | 0.29 | 0.28 | 0.28 | 0.28 |
| 388 | 39 | HN | 39 | HB2 | 0.30 | 0.26 | 0.26 | 0.31 | 0.27 |
| 389 | 39 | HN | 39 | HB1 | 0.30 | 0.24 | 0.25 | 0.29 | 0.25 |
| 390 | 39 | HA | 39 | HB2 | 0.30 | 0.25 | 0.24 | 0.24 | 0.24 |
| 391 | 39 | HA | 39 | HB1 | 0.45 | 0.29 | 0.29 | 0.29 | 0.27 |
| 392 | 39 | HA | 41 | HN | 0.45 | 0.36 | 0.40 | 0.44 | 0.42 |
| 393 | 39 | HB1 | 42 | HN | 0.45 | 0.34 | 0.33 | 0.41 | 0.37 |
| 394 | 39 | HA | 40 | HN | 0.25 | 0.21 | 0.21 | 0.24 | 0.22 |
| 395 | 39 | HA | 42 | HN | 0.55 | 0.47 | 0.49 | 0.52 | 0.51 |
| 396 | 39 | HN | 40 | HN | 0.55 | 0.44 | 0.44 | 0.44 | 0.45 |
| 397 | 39 | HA | 40 | HA | 0.55 | 0.41 | 0.41 | 0.40 | 0.41 |
| 398 | 40 | HN | 40 | HA | 0.45 | 0.27 | 0.27 | 0.27 | 0.26 |
| 399 | 40 | HA | 40 | HB | 0.25 | 0.25 | 0.26 | 0.25 | 0.26 |
| 400 | 40 | HA | 40 | HG2@ | 0.55 | 0.30 | 0.28 | 0.29 | 0.28 |
| 401 | 40 | HN | 41 | HN | 0.30 | 0.28 | 0.28 | 0.27 | 0.30 |
| 402 | 40 | HA | 55 | HN | 0.45 | 0.30 | 0.34 | 0.43 | 0.33 |
| 403 | 40 | HA | 42 | HN | 0.45 | 0.37 | 0.40 | 0.38 | 0.42 |
| 404 | 40 | HN | 40 | HG2@ | 0.55 | 0.30 | 0.32 | 0.31 | 0.32 |
| 405 | 40 | HB | 40 | HG2@ | 0.55 | 0.24 | 0.24 | 0.24 | 0.24 |
| 406 | 40 | HN | 42 | HN | 0.55 | 0.44 | 0.45 | 0.46 | 0.47 |
| 407 | 41 | HN | 41 | HA | 0.45 | 0.29 | 0.27 | 0.27 | 0.28 |
| 408 | 41 | HN | 42 | HN | 0.30 | 0.26 | 0.28 | 0.29 | 0.26 |
| 409 | 42 | HN | 42 | HA | 0.30 | 0.27 | 0.27 | 0.27 | 0.27 |
| 410 | 42 | HN | 42 | HB@ | 0.55 | 0.27 | 0.27 | 0.27 | 0.27 |
| 411 | 42 | HA | 42 | HB@ | 0.55 | 0.24 | 0.24 | 0.24 | 0.24 |
| 412 | 42 | HA | 43 | HN | 0.25 | 0.22 | 0.22 | 0.21 | 0.21 |
| 413 | 42 | HB@ | 43 | HN | 0.55 | 0.37 | 0.37 | 0.42 | 0.41 |
| 414 | 42 | HB@ | 43 | HA | 0.75 | 0.47 | 0.49 | 0.53 | 0.52 |
| 415 | 43 | HN | 43 | HA | 0.45 | 0.28 | 0.28 | 0.26 | 0.27 |
| 416 | 43 | HA | 43 | HB | 0.25 | 0.25 | 0.26 | 0.26 | 0.26 |
| 417 | 43 | HA | 44 | HN | 0.25 | 0.23 | 0.23 | 0.25 | 0.28 |
| 418 | 43 | HA | 54 | HN | 0.45 | 0.34 | 0.43 | 0.61 | 0.26 |
| 419 | 43 | HA | 53 | HA | 0.30 | 0.30 | 0.34 | 0.41 | 0.33 |
| 420 | 43 | HN | 43 | HB | 0.45 | 0.38 | 0.35 | 0.34 | 0.31 |
| 421 | 43 | HN | 43 | HG2@ | 0.75 | 0.39 | 0.38 | 0.31 | 0.34 |
| 422 | 43 | HB | 43 | HG2@ | 0.55 | 0.24 | 0.24 | 0.24 | 0.24 |
| 423 | 43 | HA | 51 | HG2@ | 0.60 | 0.43 | 0.51 | 0.55 | 0.90 |
| 424 | 44 | HN | 44 | HA | 0.30 | 0.29 | 0.28 | 0.28 | 0.24 |
| 425 | 44 | HN | 44 | HB@ | 0.65 | 0.29 | 0.25 | 0.24 | 0.31 |
| 426 | 44 | HA | 44 | HB@ | 0.45 | 0.25 | 0.25 | 0.26 | 0.25 |
| 427 | 44 | HN | 52 | HB2 | 0.45 | 0.30 | 0.29 | 0.31 | 0.42 |
| 428 | 44 | HN | 51 | HG2@ | 0.60 | 0.34 | 0.50 | 0.66 | 0.86 |
| 429 | 44 | HA | 45 | HN | 0.25 | 0.22 | 0.23 | 0.24 | 0.22 |
| 430 | 44 | HN | 45 | HN | 0.55 | 0.41 | 0.45 | 0.46 | 0.37 |
| 431 | 45 | HN | 45 | HA | 0.45 | 0.29 | 0.28 | 0.28 | 0.27 |
| 432 | 45 | HN | 45 | HB@ | 0.50 | 0.30 | 0.27 | 0.26 | 0.28 |
| 433 | 45 | HA | 45 | HB@ | 0.65 | 0.25 | 0.26 | 0.26 | 0.26 |
| 434 | 45 | HA | 46 | HN | 0.30 | 0.22 | 0.21 | 0.22 | 0.22 |
| 435 | 45 | HA | 52 | HN | 0.45 | 0.37 | 0.35 | 0.36 | 0.30 |
| 436 | 45 | HA | 51 | HA | 0.25 | 0.26 | 0.28 | 0.30 | 0.34 |
| 437 | 45 | HA | 51 | HG2@ | 0.60 | 0.36 | 0.42 | 0.48 | 0.55 |
| 438 | 46 | HN | 46 | HA | 0.45 | 0.28 | 0.28 | 0.29 | 0.28 |
| 439 | 46 | HN | 46 | HB2 | 0.30 | 0.24 | 0.22 | 0.27 | 0.23 |
| 440 | 46 | HN | 46 | HB1 | 0.45 | 0.35 | 0.28 | 0.26 | 0.34 |
| 441 | 46 | HA | 46 | HB2 | 0.45 | 0.29 | 0.26 | 0.25 | 0.28 |
| 442 | 46 | HA | 46 | HB1 | 0.45 | 0.25 | 0.28 | 0.29 | 0.26 |
| 443 | 46 | HA | 47 | HN | 0.25 | 0.23 | 0.25 | 0.23 | 0.23 |
| 444 | 46 | HB2 | 47 | HN | 0.30 | 0.37 | 0.38 | 0.39 | 0.38 |
| 445 | 46 | HB1 | 47 | HN | 0.30 | 0.27 | 0.29 | 0.33 | 0.26 |
| 446 | 46 | HB2 | 48 | HN | 0.30 | 0.31 | 0.47 | 0.48 | 0.44 |
| 447 | 46 | HB1 | 48 | HN | 0.30 | 0.26 | 0.32 | 0.35 | 0.34 |
| 448 | 46 | HN | 51 | HA | 0.45 | 0.26 | 0.32 | 0.35 | 0.42 |
| 449 | 46 | HA | 48 | HN | 0.55 | 0.40 | 0.46 | 0.44 | 0.46 |
| 450 | 46 | HN | 48 | HN | 0.75 | 0.50 | 0.53 | 0.53 | 0.56 |
| 451 | 46 | HN | 49 | HN | 0.55 | 0.47 | 0.43 | 0.50 | 0.58 |
| 452 | 47 | HN | 47 | HA | 0.30 | 0.28 | 0.27 | 0.27 | 0.25 |
| 453 | 47 | HA | 47 | HB | 0.25 | 0.25 | 0.26 | 0.26 | 0.26 |
| 454 | 47 | HB | 47 | HG2@ | 0.55 | 0.24 | 0.24 | 0.24 | 0.24 |
| 455 | 47 | HN | 48 | HN | 0.30 | 0.26 | 0.28 | 0.27 | 0.31 |
| 456 | 47 | HN | 47 | HG2@ | 0.60 | 0.31 | 0.32 | 0.33 | 0.31 |
| 457 | 47 | HN | 49 | HN | 0.55 | 0.43 | 0.42 | 0.47 | 0.50 |
| 458 | 48 | HN | 48 | HA | 0.45 | 0.28 | 0.27 | 0.27 | 0.28 |
| 459 | 48 | HN | 48 | HB2 | 0.45 | 0.36 | 0.34 | 0.32 | 0.25 |
| 460 | 48 | HN | 48 | HB1 | 0.45 | 0.26 | 0.25 | 0.26 | 0.30 |
| 461 | 48 | HA | 48 | HB2 | 0.25 | 0.23 | 0.24 | 0.24 | 0.27 |
| 462 | 48 | HA | 48 | HB1 | 0.25 | 0.24 | 0.24 | 0.24 | 0.25 |
| 463 | 49 | HN | 49 | HA@ | 0.65 | 0.24 | 0.25 | 0.23 | 0.23 |
| 464 | 49 | HN | 50 | HN | 0.45 | 0.27 | 0.26 | 0.32 | 0.30 |
| 465 | 50 | HN | 50 | HA | 0.45 | 0.28 | 0.27 | 0.27 | 0.27 |
| 466 | 50 | HN | 50 | HB@ | 0.65 | 0.30 | 0.29 | 0.31 | 0.29 |
| 467 | 50 | HA | 51 | HN | 0.45 | 0.25 | 0.24 | 0.24 | 0.22 |
| 468 | 51 | HN | 51 | HA | 0.45 | 0.29 | 0.28 | 0.28 | 0.27 |
| 469 | 51 | HN | 51 | HB | 0.45 | 0.28 | 0.31 | 0.35 | 0.34 |
| 470 | 51 | HA | 51 | HB | 0.30 | 0.29 | 0.27 | 0.25 | 0.26 |
| 471 | 51 | HB | 51 | HG2@ | 0.55 | 0.24 | 0.24 | 0.24 | 0.24 |
| 472 | 51 | HA | 51 | HG2@ | 0.55 | 0.31 | 0.28 | 0.28 | 0.28 |
| 473 | 51 | HA | 52 | HN | 0.25 | 0.22 | 0.22 | 0.23 | 0.22 |
| 474 | 51 | HG2@ | 52 | HN | 0.55 | 0.31 | 0.40 | 0.44 | 0.48 |
| 475 | 51 | HG2@ | 53 | HE@ | 1.04 | 0.35 | 0.48 | 0.55 | 0.63 |
| 476 | 51 | HG2@ | 53 | HD@ | 1.19 | 0.43 | 0.54 | 0.74 | 0.76 |
| 477 | 51 | HN | 51 | HG2@ | 0.75 | 0.46 | 0.38 | 0.34 | 0.34 |
| 478 | 51 | HB | 52 | HN | 0.45 | 0.40 | 0.34 | 0.29 | 0.37 |
| 479 | 51 | HB | 53 | HE@ | 0.74 | 0.28 | 0.37 | 0.36 | 0.44 |
| 480 | 51 | HN | 53 | HE@ | 0.99 | 0.51 | 0.60 | 0.56 | 0.72 |
| 481 | 51 | HG2@ | 59 | HA | 0.85 | 0.64 | 0.72 | 0.76 | 0.66 |
| 482 | 52 | HN | 52 | HA | 0.45 | 0.29 | 0.28 | 0.28 | 0.27 |
| 483 | 52 | HN | 52 | HB2 | 0.30 | 0.26 | 0.24 | 0.24 | 0.25 |
| 484 | 52 | HN | 52 | HB1 | 0.45 | 0.36 | 0.35 | 0.35 | 0.35 |
| 485 | 52 | HA | 52 | HB2 | 0.45 | 0.29 | 0.29 | 0.29 | 0.29 |
| 486 | 52 | HA | 53 | HN | 0.25 | 0.23 | 0.22 | 0.22 | 0.22 |
| 487 | 52 | HA | 59 | HA | 0.30 | 0.24 | 0.23 | 0.31 | 0.25 |
| 488 | 52 | HA | 53 | HE@ | 0.89 | 0.59 | 0.65 | 0.54 | 0.62 |
| 489 | 52 | HA | 57 | HN | 0.55 | 0.63 | 0.66 | 0.65 | 0.63 |
| 490 | 52 | HA | 53 | HD@ | 0.89 | 0.46 | 0.48 | 0.46 | 0.48 |
| 491 | 52 | HN | 53 | HN | 0.55 | 0.45 | 0.44 | 0.45 | 0.45 |
| 492 | 52 | HB1 | 59 | HA | 0.55 | 0.41 | 0.40 | 0.51 | 0.46 |
| 493 | 53 | HN | 53 | HB2 | 0.30 | 0.26 | 0.23 | 0.26 | 0.24 |
| 494 | 53 | HN | 53 | HB1 | 0.45 | 0.37 | 0.35 | 0.36 | 0.35 |
| 495 | 53 | HA | 53 | HB2 | 0.45 | 0.29 | 0.29 | 0.29 | 0.29 |
| 496 | 53 | HA | 53 | HB1 | 0.30 | 0.26 | 0.26 | 0.24 | 0.25 |
| 497 | 53 | HB2 | 53 | HD@ | 0.74 | 0.20 | 0.21 | 0.21 | 0.21 |
| 498 | 53 | HB1 | 53 | HD@ | 0.74 | 0.20 | 0.21 | 0.21 | 0.21 |
| 499 | 53 | HA | 54 | HN | 0.30 | 0.22 | 0.22 | 0.24 | 0.21 |
| 500 | 53 | HN | 59 | HA | 0.45 | 0.37 | 0.33 | 0.39 | 0.36 |
| 501 | 53 | HN | 57 | HA | 0.45 | 0.30 | 0.37 | 0.37 | 0.36 |
| 502 | 53 | HN | 58 | HN | 0.45 | 0.27 | 0.31 | 0.32 | 0.34 |
| 503 | 53 | HD@ | 53 | HE@ | 1.13 | 0.28 | 0.28 | 0.28 | 0.28 |
| 504 | 53 | HD@ | 53 | HA | 0.89 | 0.28 | 0.27 | 0.28 | 0.27 |
| 505 | 53 | HD@ | 60 | HN | 0.89 | 0.59 | 0.54 | 0.57 | 0.55 |
| 506 | 53 | HN | 53 | HD@ | 0.89 | 0.35 | 0.34 | 0.32 | 0.33 |
| 507 | 53 | HE@ | 60 | HN | 0.89 | 0.59 | 0.61 | 0.52 | 0.56 |
| 508 | 53 | HE@ | 60 | HB@ | 0.94 | 0.45 | 0.47 | 0.43 | 0.47 |
| 509 | 53 | HE@ | 66 | HB@ | 1.09 | 0.36 | 0.56 | 0.52 | 0.47 |
| 510 | 53 | HD@ | 59 | HA | 0.99 | 0.56 | 0.58 | 0.56 | 0.58 |
| 511 | 53 | HN | 57 | HN | 0.75 | 0.46 | 0.54 | 0.61 | 0.52 |
| 512 | 53 | HN | 60 | HN | 0.75 | 0.52 | 0.41 | 0.50 | 0.47 |
| 513 | 54 | HN | 54 | HA@ | 0.65 | 0.24 | 0.24 | 0.24 | 0.23 |
| 514 | 54 | HA@ | 55 | HN | 0.65 | 0.24 | 0.24 | 0.24 | 0.24 |
| 515 | 54 | HN | 55 | HN | 0.55 | 0.46 | 0.45 | 0.45 | 0.45 |
| 516 | 55 | HN | 55 | HA | 0.45 | 0.28 | 0.26 | 0.27 | 0.26 |
| 517 | 55 | HA | 55 | HB | 0.25 | 0.24 | 0.26 | 0.26 | 0.23 |
| 518 | 55 | HN | 55 | HB | 0.30 | 0.24 | 0.28 | 0.30 | 0.23 |
| 519 | 55 | HN | 55 | HG1@ | 0.65 | 0.43 | 0.26 | 0.26 | 0.43 |
| 520 | 55 | HG2@ | 55 | HD@ | 1.05 | 0.36 | 0.36 | 0.37 | 0.41 |
| 521 | 55 | HG2@ | 56 | HA | 0.60 | 0.48 | 0.52 | 0.58 | 0.50 |
| 522 | 55 | HG2@ | 56 | HN | 0.60 | 0.32 | 0.40 | 0.43 | 0.28 |
| 523 | 55 | HN | 55 | HG2@ | 0.60 | 0.29 | 0.34 | 0.35 | 0.28 |
| 524 | 55 | HG2@ | 56 | HD@ | 1.28 | 0.51 | 0.60 | 0.73 | 0.53 |
| 525 | 55 | HG1@ | 56 | HD@ | 1.03 | 0.61 | 0.57 | 0.71 | 0.52 |
| 526 | 55 | HN | 56 | HN | 0.55 | 0.27 | 0.29 | 0.28 | 0.32 |
| 527 | 55 | HN | 57 | HN | 0.55 | 0.40 | 0.42 | 0.59 | 0.43 |
| 528 | 55 | HG2@ | 88 | HD@ | 1.15 | 0.60 | 0.54 | 0.52 | 0.53 |
| 529 | 56 | HN | 56 | HA | 0.45 | 0.28 | 0.28 | 0.28 | 0.28 |
| 530 | 56 | HN | 56 | HB@ | 0.65 | 0.30 | 0.29 | 0.27 | 0.28 |
| 531 | 56 | HA | 56 | HB@ | 0.65 | 0.26 | 0.26 | 0.26 | 0.26 |
| 532 | 56 | HA | 56 | HD@ | 0.98 | 0.34 | 0.35 | 0.34 | 0.34 |
| 533 | 56 | HA | 57 | HN | 0.45 | 0.31 | 0.31 | 0.23 | 0.30 |
| 534 | 56 | HN | 57 | HN | 0.30 | 0.24 | 0.26 | 0.39 | 0.28 |
| 535 | 56 | HD@ | 91 | HB@ | 1.03 | 0.58 | 0.51 | 0.95 | 0.59 |
| 536 | 56 | HB@ | 56 | HD@ | 1.03 | 0.26 | 0.26 | 0.26 | 0.26 |
| 537 | 56 | HB@ | 56 | HG | 0.65 | 0.25 | 0.25 | 0.25 | 0.25 |
| 538 | 56 | HD@ | 108 | HE3 | 0.98 | 0.68 | 0.88 | 0.95 | 1.02 |
| 539 | 56 | HD@ | 108 | HZ2 | 0.98 | 0.45 | 0.51 | 0.49 | 0.72 |
| 540 | 56 | HB@ | 108 | HZ2 | 0.65 | 0.38 | 0.32 | 0.53 | 0.55 |
| 541 | 56 | HD@ | 108 | HE1 | 0.98 | 0.44 | 0.58 | 0.53 | 0.95 |
| 542 | 56 | HB@ | 108 | HE1 | 0.65 | 0.28 | 0.41 | 0.58 | 0.77 |
| 543 | 57 | HN | 57 | HA | 0.25 | 0.22 | 0.21 | 0.25 | 0.21 |
| 544 | 57 | HN | 57 | HB@ | 0.65 | 0.36 | 0.36 | 0.29 | 0.35 |
| 545 | 57 | HA | 58 | HN | 0.30 | 0.25 | 0.22 | 0.22 | 0.27 |
| 546 | 57 | HN | 58 | HN | 0.45 | 0.30 | 0.31 | 0.37 | 0.31 |
| 547 | 57 | HN | 58 | HG1@ | 0.65 | 0.50 | 0.47 | 0.53 | 0.56 |
| 548 | 57 | HN | 58 | HD@ | 0.60 | 0.60 | 0.58 | 0.65 | 0.66 |
| 549 | 58 | HN | 58 | HA | 0.45 | 0.28 | 0.28 | 0.28 | 0.28 |
| 550 | 58 | HN | 58 | HB | 0.45 | 0.23 | 0.24 | 0.24 | 0.24 |
| 551 | 58 | HA | 59 | HN | 0.30 | 0.22 | 0.21 | 0.22 | 0.21 |
| 552 | 58 | HG2@ | 63 | HD1 | 0.75 | 0.38 | 0.37 | 0.54 | 0.87 |
| 553 | 58 | HG2@ | 59 | HN | 0.75 | 0.32 | 0.37 | 0.36 | 0.40 |
| 554 | 58 | HD@ | 95 | HN | 0.75 | 0.37 | 0.44 | 0.45 | 0.44 |
| 555 | 58 | HN | 59 | HA | 0.75 | 0.52 | 0.50 | 0.51 | 0.54 |
| 556 | 58 | HN | 60 | HN | 0.55 | 0.71 | 0.65 | 0.70 | 0.70 |
| 557 | 59 | HN | 59 | HA | 0.45 | 0.29 | 0.27 | 0.28 | 0.27 |
| 558 | 59 | HN | 59 | HB2 | 0.30 | 0.26 | 0.23 | 0.32 | 0.24 |
| 559 | 59 | HN | 59 | HB1 | 0.30 | 0.24 | 0.25 | 0.24 | 0.24 |
| 560 | 59 | HA | 59 | HB2 | 0.30 | 0.25 | 0.25 | 0.23 | 0.24 |
| 561 | 59 | HA | 59 | HB1 | 0.45 | 0.29 | 0.29 | 0.25 | 0.28 |
| 562 | 59 | HA | 60 | HN | 0.25 | 0.22 | 0.21 | 0.22 | 0.22 |
| 563 | 59 | HB2 | 60 | HN | 0.45 | 0.39 | 0.41 | 0.29 | 0.40 |
| 564 | 59 | HB1 | 60 | HN | 0.55 | 0.38 | 0.39 | 0.40 | 0.39 |
| 565 | 59 | HB1 | 63 | HD1 | 0.25 | 0.27 | 0.30 | 0.29 | 0.83 |
| 566 | 59 | HA | 61 | HN | 0.55 | 0.41 | 0.43 | 0.42 | 0.40 |
| 567 | 59 | HA | 63 | HE1 | 0.75 | 0.70 | 0.68 | 0.72 | 1.22 |
| 568 | 59 | HN | 60 | HN | 0.55 | 0.46 | 0.46 | 0.46 | 0.46 |
| 569 | 60 | HN | 60 | HA | 0.30 | 0.28 | 0.26 | 0.27 | 0.27 |
| 570 | 60 | HN | 60 | HB@ | 0.65 | 0.30 | 0.30 | 0.30 | 0.29 |
| 571 | 60 | HA | 60 | HB@ | 0.45 | 0.23 | 0.23 | 0.24 | 0.23 |
| 572 | 60 | HN | 61 | HN | 0.45 | 0.28 | 0.29 | 0.28 | 0.28 |
| 573 | 60 | HA | 65 | HA | 0.75 | 0.47 | 0.55 | 0.50 | 0.63 |
| 574 | 60 | HA | 64 | HA | 0.55 | 0.48 | 0.52 | 0.53 | 0.68 |
| 575 | 60 | HN | 62 | HN | 0.75 | 0.52 | 0.44 | 0.49 | 0.45 |
| 576 | 60 | HN | 64 | HN | 0.75 | 0.49 | 0.50 | 0.50 | 0.73 |
| 577 | 61 | HN | 61 | HA | 0.45 | 0.29 | 0.27 | 0.27 | 0.27 |
| 578 | 61 | HN | 61 | HB2 | 0.30 | 0.26 | 0.23 | 0.23 | 0.24 |
| 579 | 61 | HN | 61 | HB1 | 0.30 | 0.26 | 0.28 | 0.31 | 0.33 |
| 580 | 61 | HN | 62 | HN | 0.30 | 0.28 | 0.27 | 0.29 | 0.28 |
| 581 | 61 | HA | 72 | HA | 0.30 | 0.31 | 0.29 | 0.36 | 0.48 |
| 582 | 61 | HN | 63 | HN | 0.55 | 0.37 | 0.47 | 0.45 | 0.70 |
| 583 | 62 | HN | 62 | HA | 0.45 | 0.27 | 0.27 | 0.27 | 0.28 |
| 584 | 62 | HH2 | 62 | HZ2 | 0.25 | 0.24 | 0.25 | 0.25 | 0.25 |
| 585 | 62 | HZ3 | 62 | HH2 | 0.45 | 0.25 | 0.25 | 0.25 | 0.25 |
| 586 | 63 | HN | 63 | HA | 0.45 | 0.28 | 0.28 | 0.27 | 0.25 |
| 587 | 63 | HA | 63 | HB@ | 0.50 | 0.26 | 0.26 | 0.26 | 0.26 |
| 588 | 63 | HE3 | 63 | HZ3 | 0.25 | 0.25 | 0.25 | 0.25 | 0.25 |
| 589 | 63 | HH2 | 63 | HZ2 | 0.45 | 0.25 | 0.25 | 0.25 | 0.25 |
| 590 | 63 | HE3 | 63 | HB@ | 0.65 | 0.35 | 0.34 | 0.34 | 0.28 |
| 591 | 63 | HD1 | 63 | HB@ | 0.65 | 0.31 | 0.30 | 0.30 | 0.35 |
| 592 | 63 | HE3 | 63 | HA | 0.30 | 0.25 | 0.29 | 0.27 | 0.47 |
| 593 | 63 | HE3 | 76 | HN | 0.45 | 0.32 | 0.35 | 0.29 | 0.38 |
| 594 | 63 | HZ3 | 75 | HN | 0.45 | 0.52 | 0.58 | 0.57 | 0.71 |
| 595 | 63 | HZ3 | 63 | HA | 0.45 | 0.46 | 0.50 | 0.48 | 0.68 |
| 596 | 63 | HB@ | 76 | HA | 0.65 | 0.62 | 0.56 | 0.63 | 0.51 |
| 597 | 63 | HA | 75 | HN | 0.30 | 0.24 | 0.31 | 0.30 | 0.34 |
| 598 | 63 | HA | 76 | HN | 0.45 | 0.29 | 0.31 | 0.32 | 0.40 |
| 599 | 63 | HE3 | 75 | HB2 | 0.30 | 0.24 | 0.27 | 0.27 | 0.34 |
| 600 | 63 | HZ3 | 75 | HB1 | 0.45 | 0.29 | 0.29 | 0.29 | 0.39 |
| 601 | 63 | HZ3 | 75 | HB2 | 0.45 | 0.27 | 0.36 | 0.35 | 0.49 |
| 602 | 63 | HD1 | 98 | HG1@ | 0.65 | 0.50 | 0.49 | 0.46 | 0.40 |
| 603 | 63 | HE3 | 98 | HG1@ | 0.65 | 0.34 | 0.35 | 0.42 | 0.39 |
| 604 | 63 | HA | 76 | HB@ | 0.45 | 0.49 | 0.47 | 0.50 | 0.58 |
| 605 | 63 | HZ2 | 98 | HG2@ | 0.60 | 0.40 | 0.39 | 0.46 | 0.65 |
| 606 | 63 | HZ2 | 98 | HG1@ | 0.65 | 0.42 | 0.46 | 0.42 | 0.55 |
| 607 | 63 | HE1 | 63 | HB@ | 0.75 | 0.49 | 0.49 | 0.48 | 0.50 |
| 608 | 63 | HE1 | 98 | HG2@ | 0.85 | 0.48 | 0.43 | 0.39 | 0.53 |
| 609 | 63 | HE1 | 98 | HG1@ | 0.95 | 0.46 | 0.47 | 0.41 | 0.47 |
| 610 | 63 | HD1 | 98 | HD@ | 0.75 | 0.46 | 0.45 | 0.45 | 0.51 |
| 611 | 63 | HE3 | 75 | HD@ | 0.83 | 0.52 | 0.47 | 0.52 | 0.50 |
| 612 | 63 | HZ3 | 75 | HD@ | 0.98 | 0.44 | 0.41 | 0.48 | 0.53 |
| 613 | 63 | HE3 | 75 | HN | 0.55 | 0.38 | 0.46 | 0.44 | 0.54 |
| 614 | 63 | HN | 75 | HN | 0.75 | 0.44 | 0.52 | 0.49 | 0.58 |
| 615 | 63 | HZ3 | 101 | HB@ | 0.75 | 0.38 | 0.39 | 0.48 | 0.54 |
| 616 | 63 | HE3 | 98 | HG2@ | 1.05 | 0.63 | 0.59 | 0.64 | 0.68 |
| 617 | 63 | HH2 | 98 | HG2@ | 0.85 | 0.47 | 0.47 | 0.61 | 0.77 |
| 618 | 64 | HN | 64 | HA | 0.30 | 0.29 | 0.28 | 0.28 | 0.28 |
| 619 | 64 | HA | 64 | HB@ | 0.50 | 0.21 | 0.21 | 0.21 | 0.21 |
| 620 | 64 | HA | 78 | HN | 0.45 | 0.33 | 0.47 | 0.56 | 0.47 |
| 621 | 64 | HB@ | 80 | HN | 0.65 | 0.41 | 0.42 | 0.35 | 0.38 |
| 622 | 64 | HA | 78 | HG2@ | 0.60 | 0.65 | 0.70 | 0.67 | 0.51 |
| 623 | 64 | HN | 64 | HB@ | 0.65 | 0.34 | 0.31 | 0.33 | 0.33 |
| 624 | 64 | HA | 65 | HN | 0.30 | 0.24 | 0.23 | 0.23 | 0.24 |
| 625 | 64 | HA | 76 | HN | 0.55 | 0.39 | 0.36 | 0.38 | 0.35 |
| 626 | 64 | HA | 77 | HN | 0.55 | 0.45 | 0.45 | 0.46 | 0.38 |
| 627 | 64 | HA | 65 | HA | 0.75 | 0.43 | 0.42 | 0.43 | 0.42 |
| 628 | 64 | HA | 74 | HA | 0.30 | 0.31 | 0.42 | 0.26 | 0.38 |
| 629 | 64 | HA | 74 | HB@ | 0.75 | 0.47 | 0.62 | 0.47 | 0.63 |
| 630 | 65 | HN | 65 | HA | 0.45 | 0.29 | 0.28 | 0.27 | 0.28 |
| 631 | 65 | HN | 65 | HB@ | 0.50 | 0.25 | 0.24 | 0.23 | 0.24 |
| 632 | 65 | HA | 65 | HB@ | 0.65 | 0.26 | 0.25 | 0.26 | 0.25 |
| 633 | 65 | HA | 66 | HN | 0.30 | 0.22 | 0.21 | 0.22 | 0.22 |
| 634 | 65 | HA | 67 | HN | 0.45 | 0.34 | 0.46 | 0.38 | 0.40 |
| 635 | 65 | HN | 78 | HG2@ | 0.75 | 0.68 | 0.68 | 0.75 | 0.54 |
| 636 | 65 | HB@ | 66 | HN | 0.75 | 0.42 | 0.41 | 0.40 | 0.37 |
| 637 | 65 | HN | 66 | HN | 0.55 | 0.44 | 0.44 | 0.45 | 0.45 |
| 638 | 65 | HN | 78 | HN | 0.55 | 0.37 | 0.47 | 0.62 | 0.48 |
| 639 | 66 | HN | 66 | HA | 0.45 | 0.28 | 0.28 | 0.28 | 0.27 |
| 640 | 66 | HN | 66 | HB1 | 0.45 | 0.30 | 0.26 | 0.28 | 0.34 |
| 641 | 66 | HA | 66 | HB2 | 0.25 | 0.23 | 0.24 | 0.25 | 0.27 |
| 642 | 66 | HA | 66 | HB1 | 0.25 | 0.24 | 0.28 | 0.28 | 0.27 |
| 643 | 66 | HA | 67 | HN | 0.45 | 0.32 | 0.25 | 0.29 | 0.33 |
| 644 | 66 | HN | 67 | HN | 0.30 | 0.22 | 0.30 | 0.25 | 0.21 |
| 645 | 66 | HN | 67 | HA@ | 0.75 | 0.45 | 0.50 | 0.45 | 0.42 |
| 646 | 66 | HN | 68 | HN | 0.75 | 0.35 | 0.52 | 0.50 | 0.52 |
| 647 | 66 | HN | 69 | HN | 0.75 | 0.43 | 0.54 | 0.42 | 0.57 |
| 648 | 67 | HN | 67 | HA@ | 0.65 | 0.24 | 0.24 | 0.23 | 0.23 |
| 649 | 67 | HN | 68 | HA | 0.55 | 0.52 | 0.52 | 0.57 | 0.56 |
| 650 | 68 | HN | 68 | HA | 0.45 | 0.28 | 0.28 | 0.27 | 0.27 |
| 651 | 68 | HA | 68 | HB@ | 0.50 | 0.26 | 0.26 | 0.24 | 0.24 |
| 652 | 68 | HN | 68 | HB@ | 0.65 | 0.32 | 0.28 | 0.28 | 0.28 |
| 653 | 68 | HA | 69 | HN | 0.45 | 0.31 | 0.34 | 0.31 | 0.33 |
| 654 | 69 | HA | 69 | HB | 0.45 | 0.30 | 0.26 | 0.26 | 0.25 |
| 655 | 69 | HN | 69 | HB | 0.45 | 0.26 | 0.29 | 0.35 | 0.35 |
| 656 | 69 | HB | 69 | HG2@ | 0.55 | 0.24 | 0.24 | 0.24 | 0.24 |
| 657 | 71 | HN | 71 | HA@ | 0.50 | 0.24 | 0.23 | 0.25 | 0.25 |
| 658 | 71 | HA@ | 72 | HN | 0.65 | 0.31 | 0.31 | 0.28 | 0.27 |
| 659 | 71 | HN | 72 | HN | 0.30 | 0.27 | 0.28 | 0.37 | 0.41 |
| 660 | 72 | HN | 72 | HA | 0.45 | 0.28 | 0.27 | 0.21 | 0.21 |
| 661 | 72 | HN | 72 | HB@ | 0.50 | 0.24 | 0.28 | 0.34 | 0.33 |
| 662 | 72 | HA | 72 | HB@ | 0.50 | 0.26 | 0.25 | 0.26 | 0.26 |
| 663 | 72 | HA | 73 | HN | 0.45 | 0.21 | 0.22 | 0.25 | 0.25 |
| 664 | 72 | HA | 74 | HN | 0.55 | 0.36 | 0.41 | 0.37 | 0.44 |
| 665 | 74 | HN | 74 | HA | 0.25 | 0.22 | 0.20 | 0.21 | 0.25 |
| 666 | 74 | HA | 74 | HB@ | 0.65 | 0.25 | 0.25 | 0.26 | 0.26 |
| 667 | 74 | HA | 75 | HN | 0.45 | 0.25 | 0.27 | 0.25 | 0.25 |
| 668 | 74 | HA | 76 | HN | 0.45 | 0.35 | 0.38 | 0.36 | 0.40 |
| 669 | 74 | HN | 74 | HB@ | 0.65 | 0.31 | 0.35 | 0.32 | 0.25 |
| 670 | 74 | HN | 75 | HN | 0.45 | 0.32 | 0.29 | 0.28 | 0.38 |
| 671 | 74 | HN | 76 | HN | 0.75 | 0.52 | 0.51 | 0.51 | 0.59 |
| 672 | 75 | HN | 75 | HA | 0.45 | 0.28 | 0.27 | 0.26 | 0.27 |
| 673 | 75 | HN | 75 | HB2 | 0.30 | 0.25 | 0.23 | 0.23 | 0.23 |
| 674 | 75 | HN | 75 | HB1 | 0.45 | 0.36 | 0.34 | 0.34 | 0.34 |
| 675 | 75 | HA | 75 | HB2 | 0.45 | 0.29 | 0.29 | 0.29 | 0.28 |
| 676 | 75 | HA | 75 | HB1 | 0.45 | 0.24 | 0.26 | 0.25 | 0.25 |
| 677 | 75 | HN | 75 | HG | 0.30 | 0.24 | 0.24 | 0.24 | 0.28 |
| 678 | 75 | HA | 76 | HN | 0.45 | 0.34 | 0.34 | 0.35 | 0.35 |
| 679 | 75 | HN | 76 | HN | 0.45 | 0.26 | 0.29 | 0.29 | 0.27 |
| 680 | 75 | HB2 | 76 | HN | 0.45 | 0.32 | 0.31 | 0.29 | 0.27 |
| 681 | 75 | HN | 75 | HD@ | 0.98 | 0.40 | 0.40 | 0.39 | 0.41 |
| 682 | 75 | HB1 | 76 | HB@ | 0.50 | 0.63 | 0.57 | 0.57 | 0.56 |
| 683 | 75 | HG | 76 | HN | 0.75 | 0.46 | 0.47 | 0.47 | 0.46 |
| 684 | 75 | HD@ | 76 | HN | 1.08 | 0.57 | 0.56 | 0.55 | 0.53 |
| 685 | 76 | HN | 76 | HA | 0.45 | 0.28 | 0.28 | 0.27 | 0.27 |
| 686 | 76 | HN | 76 | HB@ | 0.50 | 0.29 | 0.27 | 0.27 | 0.28 |
| 687 | 76 | HN | 77 | HN | 0.30 | 0.26 | 0.27 | 0.28 | 0.28 |
| 688 | 76 | HN | 78 | HG2@ | 0.75 | 0.71 | 0.77 | 0.67 | 0.64 |
| 689 | 76 | HA | 76 | HB@ | 0.45 | 0.26 | 0.26 | 0.26 | 0.25 |
| 690 | 76 | HA | 77 | HN | 0.45 | 0.33 | 0.31 | 0.31 | 0.32 |
| 691 | 76 | HB@ | 78 | HG2@ | 0.75 | 0.49 | 0.56 | 0.42 | 0.37 |
| 692 | 76 | HN | 77 | HA | 0.55 | 0.46 | 0.45 | 0.47 | 0.46 |
| 693 | 76 | HN | 77 | HB@ | 0.95 | 0.55 | 0.60 | 0.61 | 0.61 |
| 694 | 76 | HN | 78 | HN | 0.55 | 0.42 | 0.49 | 0.50 | 0.50 |
| 695 | 77 | HA | 77 | HB@ | 0.45 | 0.26 | 0.25 | 0.26 | 0.26 |
| 696 | 77 | HB@ | 78 | HN | 0.65 | 0.44 | 0.33 | 0.32 | 0.34 |
| 697 | 77 | HN | 77 | HB@ | 0.65 | 0.33 | 0.35 | 0.36 | 0.35 |
| 698 | 77 | HN | 78 | HN | 0.45 | 0.29 | 0.35 | 0.35 | 0.35 |
| 699 | 77 | HA | 78 | HN | 0.30 | 0.27 | 0.34 | 0.34 | 0.34 |
| 700 | 78 | HN | 78 | HA | 0.45 | 0.29 | 0.27 | 0.28 | 0.27 |
| 701 | 78 | HA | 78 | HB | 0.25 | 0.26 | 0.25 | 0.24 | 0.25 |
| 702 | 78 | HA | 78 | HG2@ | 0.55 | 0.29 | 0.28 | 0.32 | 0.34 |
| 703 | 78 | HN | 78 | HG1@ | 0.50 | 0.25 | 0.24 | 0.30 | 0.32 |
| 704 | 78 | HN | 78 | HG2@ | 0.75 | 0.42 | 0.31 | 0.30 | 0.31 |
| 705 | 79 | HA | 79 | HB@ | 0.50 | 0.24 | 0.25 | 0.24 | 0.24 |
| 706 | 79 | HA | 80 | HN | 0.25 | 0.22 | 0.22 | 0.23 | 0.22 |
| 707 | 80 | HN | 80 | HA | 0.45 | 0.27 | 0.27 | 0.27 | 0.27 |
| 708 | 80 | HA | 83 | HN | 0.45 | 0.34 | 0.34 | 0.33 | 0.41 |
| 709 | 80 | HN | 81 | HN | 0.45 | 0.28 | 0.28 | 0.28 | 0.27 |
| 710 | 81 | HN | 81 | HA | 0.30 | 0.27 | 0.26 | 0.27 | 0.26 |
| 711 | 81 | HN | 82 | HN | 0.30 | 0.28 | 0.30 | 0.28 | 0.29 |
| 712 | 81 | HA | 84 | HN | 0.55 | 0.35 | 0.36 | 0.36 | 0.40 |
| 713 | 81 | HN | 83 | HN | 0.55 | 0.43 | 0.45 | 0.42 | 0.46 |
| 714 | 82 | HN | 82 | HA | 0.30 | 0.28 | 0.27 | 0.27 | 0.27 |
| 715 | 82 | HN | 82 | HB@ | 0.55 | 0.28 | 0.27 | 0.27 | 0.27 |
| 716 | 82 | HA | 82 | HB@ | 0.55 | 0.24 | 0.24 | 0.24 | 0.24 |
| 717 | 82 | HN | 83 | HN | 0.30 | 0.26 | 0.28 | 0.27 | 0.29 |
| 718 | 82 | HB@ | 83 | HN | 0.75 | 0.35 | 0.35 | 0.33 | 0.36 |
| 719 | 82 | HA | 84 | HN | 0.55 | 0.36 | 0.40 | 0.41 | 0.36 |
| 720 | 83 | HN | 83 | HA | 0.30 | 0.27 | 0.27 | 0.27 | 0.27 |
| 721 | 83 | HN | 83 | HB@ | 0.50 | 0.30 | 0.28 | 0.28 | 0.27 |
| 722 | 83 | HA | 83 | HB@ | 0.50 | 0.26 | 0.26 | 0.26 | 0.26 |
| 723 | 83 | HA | 83 | HD@ | 0.78 | 0.35 | 0.35 | 0.34 | 0.31 |
| 724 | 83 | HA | 84 | HN | 0.45 | 0.33 | 0.34 | 0.34 | 0.32 |
| 725 | 83 | HN | 84 | HN | 0.30 | 0.27 | 0.27 | 0.28 | 0.29 |
| 726 | 83 | HN | 83 | HG | 0.30 | 0.24 | 0.24 | 0.23 | 0.29 |
| 727 | 83 | HN | 83 | HD@ | 0.98 | 0.40 | 0.39 | 0.39 | 0.38 |
| 728 | 84 | HN | 84 | HA | 0.45 | 0.29 | 0.27 | 0.27 | 0.27 |
| 729 | 84 | HA | 84 | HB@ | 0.65 | 0.25 | 0.26 | 0.26 | 0.26 |
| 730 | 84 | HN | 84 | HB@ | 0.50 | 0.30 | 0.28 | 0.25 | 0.28 |
| 731 | 84 | HA | 85 | HN | 0.45 | 0.31 | 0.31 | 0.33 | 0.27 |
| 732 | 85 | HN | 85 | HA | 0.30 | 0.28 | 0.27 | 0.28 | 0.27 |
| 733 | 85 | HN | 85 | HB@ | 0.50 | 0.28 | 0.26 | 0.26 | 0.33 |
| 734 | 85 | HA | 85 | HB@ | 0.50 | 0.26 | 0.25 | 0.25 | 0.24 |
| 735 | 85 | HA | 86 | HN | 0.25 | 0.23 | 0.23 | 0.24 | 0.27 |
| 736 | 86 | HN | 86 | HA | 0.30 | 0.27 | 0.27 | 0.27 | 0.27 |
| 737 | 86 | HN | 86 | HB@ | 0.65 | 0.30 | 0.29 | 0.29 | 0.30 |
| 738 | 86 | HA | 86 | HB@ | 0.45 | 0.26 | 0.25 | 0.26 | 0.24 |
| 739 | 86 | HN | 87 | HN | 0.30 | 0.26 | 0.29 | 0.34 | 0.32 |
| 740 | 87 | HA | 87 | HB2 | 0.45 | 0.24 | 0.24 | 0.25 | 0.27 |
| 741 | 87 | HA | 87 | HB1 | 0.30 | 0.29 | 0.29 | 0.25 | 0.27 |
| 742 | 87 | HA | 88 | HN | 0.25 | 0.23 | 0.22 | 0.24 | 0.24 |
| 743 | 87 | HN | 87 | HA | 0.45 | 0.28 | 0.27 | 0.28 | 0.28 |
| 744 | 87 | HN | 87 | HB2 | 0.45 | 0.27 | 0.25 | 0.25 | 0.23 |
| 745 | 87 | HN | 87 | HB1 | 0.45 | 0.24 | 0.24 | 0.27 | 0.29 |
| 746 | 88 | HN | 88 | HG2@ | 0.60 | 0.33 | 0.33 | 0.32 | 0.36 |
| 747 | 88 | HN | 88 | HD@ | 0.75 | 0.45 | 0.43 | 0.42 | 0.39 |
| 748 | 88 | HG1@ | 89 | HN | 0.65 | 0.38 | 0.44 | 0.35 | 0.40 |
| 749 | 88 | HG2@ | 89 | HN | 0.75 | 0.52 | 0.44 | 0.37 | 0.43 |
| 750 | 88 | HD@ | 89 | HN | 0.75 | 0.59 | 0.54 | 0.46 | 0.51 |
| 751 | 88 | HN | 88 | HG1@ | 0.50 | 0.26 | 0.29 | 0.28 | 0.26 |
| 752 | 88 | HD@ | 88 | HG1@ | 0.95 | 0.22 | 0.22 | 0.22 | 0.22 |
| 753 | 88 | HD@ | 92 | HG2@ | 0.85 | 0.33 | 0.47 | 0.55 | 0.62 |
| 754 | 88 | HB | 88 | HD@ | 0.60 | 0.29 | 0.29 | 0.29 | 0.30 |
| 755 | 88 | HD@ | 88 | HG2@ | 0.85 | 0.35 | 0.39 | 0.38 | 0.36 |
| 756 | 88 | HB | 88 | HG2@ | 0.60 | 0.24 | 0.24 | 0.24 | 0.24 |
| 757 | 88 | HB | 88 | HG1@ | 0.65 | 0.25 | 0.25 | 0.25 | 0.24 |
| 758 | 89 | HA | 92 | HN | 0.45 | 0.38 | 0.35 | 0.36 | 0.45 |
| 759 | 89 | HA | 92 | HB | 0.30 | 0.32 | 0.33 | 0.33 | 0.48 |
| 760 | 89 | HA | 90 | HN | 0.45 | 0.35 | 0.35 | 0.35 | 0.23 |
| 761 | 89 | HN | 90 | HN | 0.45 | 0.31 | 0.29 | 0.29 | 0.40 |
| 762 | 89 | HN | 89 | HG2@ | 0.75 | 0.44 | 0.33 | 0.32 | 0.36 |
| 763 | 89 | HA | 89 | HG2@ | 0.55 | 0.31 | 0.28 | 0.28 | 0.28 |
| 764 | 89 | HB | 89 | HG2@ | 0.55 | 0.24 | 0.24 | 0.24 | 0.24 |
| 765 | 89 | HG2@ | 90 | HN | 0.75 | 0.39 | 0.46 | 0.46 | 0.38 |
| 766 | 89 | HG2@ | 93 | HN | 0.75 | 0.51 | 0.61 | 0.66 | 0.72 |
| 767 | 90 | HN | 90 | HA | 0.25 | 0.28 | 0.27 | 0.27 | 0.21 |
| 768 | 90 | HN | 90 | HB@ | 0.55 | 0.28 | 0.27 | 0.27 | 0.32 |
| 769 | 90 | HA | 90 | HB@ | 0.55 | 0.24 | 0.24 | 0.24 | 0.24 |
| 770 | 90 | HA | 93 | HN | 0.45 | 0.34 | 0.36 | 0.35 | 0.35 |
| 771 | 90 | HN | 91 | HN | 0.30 | 0.27 | 0.29 | 0.29 | 0.33 |
| 772 | 90 | HB@ | 91 | HN | 0.60 | 0.34 | 0.32 | 0.33 | 0.35 |
| 773 | 91 | HN | 91 | HA | 0.30 | 0.27 | 0.26 | 0.27 | 0.27 |
| 774 | 91 | HN | 91 | HB@ | 0.65 | 0.29 | 0.27 | 0.25 | 0.29 |
| 775 | 91 | HA | 92 | HN | 0.45 | 0.35 | 0.35 | 0.35 | 0.35 |
| 776 | 91 | HA | 94 | HN | 0.45 | 0.35 | 0.37 | 0.36 | 0.35 |
| 777 | 91 | HB@ | 92 | HN | 0.65 | 0.31 | 0.30 | 0.32 | 0.34 |
| 778 | 91 | HN | 92 | HN | 0.30 | 0.28 | 0.30 | 0.29 | 0.29 |
| 779 | 92 | HN | 92 | HA | 0.30 | 0.28 | 0.27 | 0.27 | 0.27 |
| 780 | 92 | HN | 92 | HB | 0.25 | 0.24 | 0.25 | 0.24 | 0.24 |
| 781 | 92 | HA | 92 | HB | 0.30 | 0.29 | 0.27 | 0.28 | 0.25 |
| 782 | 92 | HA | 92 | HG2@ | 0.55 | 0.29 | 0.30 | 0.29 | 0.29 |
| 783 | 92 | HA | 92 | HG1@ | 0.55 | 0.31 | 0.30 | 0.30 | 0.33 |
| 784 | 92 | HN | 92 | HG2@ | 0.55 | 0.31 | 0.30 | 0.29 | 0.34 |
| 785 | 92 | HN | 92 | HG1@ | 0.75 | 0.44 | 0.38 | 0.41 | 0.33 |
| 786 | 92 | HA | 95 | HN | 0.30 | 0.34 | 0.34 | 0.33 | 0.32 |
| 787 | 92 | HN | 93 | HN | 0.30 | 0.27 | 0.29 | 0.29 | 0.30 |
| 788 | 92 | HA | 95 | HB@ | 0.55 | 0.34 | 0.33 | 0.31 | 0.32 |
| 789 | 92 | HG2@ | 93 | HN | 0.75 | 0.47 | 0.41 | 0.46 | 0.42 |
| 790 | 92 | HB | 93 | HN | 0.30 | 0.28 | 0.28 | 0.27 | 0.33 |
| 791 | 92 | HG1@ | 93 | HN | 0.75 | 0.41 | 0.40 | 0.41 | 0.33 |
| 792 | 92 | HG1@ | 96 | HA | 0.60 | 0.62 | 0.69 | 0.65 | 0.75 |
| 793 | 92 | HB | 92 | HG2@ | 0.55 | 0.23 | 0.24 | 0.24 | 0.24 |
| 794 | 92 | HB | 92 | HG1@ | 0.60 | 0.24 | 0.24 | 0.24 | 0.23 |
| 795 | 92 | HN | 94 | HN | 0.55 | 0.42 | 0.44 | 0.45 | 0.45 |
| 796 | 93 | HN | 93 | HA | 0.25 | 0.28 | 0.27 | 0.26 | 0.27 |
| 797 | 93 | HN | 93 | HB2 | 0.30 | 0.24 | 0.23 | 0.23 | 0.26 |
| 798 | 93 | HN | 93 | HB1 | 0.25 | 0.35 | 0.32 | 0.33 | 0.34 |
| 799 | 93 | HA | 93 | HB2 | 0.30 | 0.29 | 0.28 | 0.28 | 0.29 |
| 800 | 93 | HA | 93 | HB1 | 0.25 | 0.25 | 0.26 | 0.26 | 0.24 |
| 801 | 93 | HB2 | 94 | HN | 0.45 | 0.29 | 0.28 | 0.28 | 0.26 |
| 802 | 93 | HA | 96 | HN | 0.45 | 0.34 | 0.37 | 0.36 | 0.34 |
| 803 | 93 | HN | 94 | HN | 0.30 | 0.27 | 0.30 | 0.30 | 0.29 |
| 804 | 94 | HN | 94 | HA | 0.30 | 0.28 | 0.27 | 0.27 | 0.27 |
| 805 | 94 | HN | 94 | HB2 | 0.30 | 0.25 | 0.23 | 0.24 | 0.23 |
| 806 | 94 | HN | 94 | HB1 | 0.30 | 0.24 | 0.25 | 0.24 | 0.24 |
| 807 | 94 | HA | 94 | HB2 | 0.30 | 0.23 | 0.24 | 0.23 | 0.23 |
| 808 | 94 | HA | 95 | HN | 0.45 | 0.35 | 0.35 | 0.35 | 0.35 |
| 809 | 94 | HB1 | 95 | HN | 0.30 | 0.27 | 0.26 | 0.26 | 0.25 |
| 810 | 94 | HA | 97 | HN | 0.45 | 0.31 | 0.34 | 0.34 | 0.34 |
| 811 | 94 | HN | 95 | HN | 0.30 | 0.27 | 0.29 | 0.29 | 0.29 |
| 812 | 95 | HN | 95 | HA | 0.30 | 0.27 | 0.26 | 0.26 | 0.26 |
| 813 | 95 | HN | 95 | HB@ | 0.55 | 0.27 | 0.27 | 0.27 | 0.27 |
| 814 | 95 | HA | 95 | HB@ | 0.55 | 0.24 | 0.24 | 0.24 | 0.24 |
| 815 | 95 | HN | 96 | HN | 0.30 | 0.28 | 0.30 | 0.30 | 0.30 |
| 816 | 95 | HN | 97 | HN | 0.45 | 0.42 | 0.45 | 0.45 | 0.45 |
| 817 | 95 | HB@ | 96 | HN | 0.60 | 0.33 | 0.33 | 0.33 | 0.33 |
| 818 | 95 | HB@ | 108 | HH2 | 0.55 | 0.35 | 0.61 | 0.88 | 0.53 |
| 819 | 95 | HB@ | 108 | HZ2 | 0.60 | 0.34 | 0.67 | 0.79 | 0.65 |
| 820 | 95 | HA | 108 | HZ2 | 0.30 | 0.27 | 0.59 | 0.68 | 0.72 |
| 821 | 95 | HN | 98 | HG2@ | 1.05 | 0.76 | 0.74 | 0.69 | 0.74 |
| 822 | 96 | HN | 96 | HA | 0.30 | 0.27 | 0.26 | 0.26 | 0.27 |
| 823 | 96 | HN | 96 | HB@ | 0.45 | 0.29 | 0.28 | 0.26 | 0.24 |
| 824 | 96 | HA | 96 | HB@ | 0.50 | 0.26 | 0.26 | 0.26 | 0.25 |
| 825 | 96 | HN | 97 | HN | 0.30 | 0.28 | 0.29 | 0.30 | 0.30 |
| 826 | 97 | HN | 97 | HA | 0.30 | 0.28 | 0.27 | 0.27 | 0.27 |
| 827 | 97 | HA | 98 | HN | 0.45 | 0.35 | 0.35 | 0.35 | 0.35 |
| 828 | 97 | HN | 98 | HN | 0.30 | 0.28 | 0.28 | 0.28 | 0.29 |
| 829 | 97 | HN | 99 | HN | 0.45 | 0.43 | 0.46 | 0.47 | 0.45 |
| 830 | 98 | HN | 98 | HB | 0.30 | 0.24 | 0.24 | 0.25 | 0.24 |
| 831 | 98 | HN | 98 | HG2@ | 0.75 | 0.43 | 0.43 | 0.39 | 0.43 |
| 832 | 98 | HN | 98 | HG1@ | 0.50 | 0.28 | 0.26 | 0.26 | 0.26 |
| 833 | 98 | HN | 98 | HD@ | 0.75 | 0.40 | 0.40 | 0.41 | 0.39 |
| 834 | 98 | HN | 99 | HN | 0.45 | 0.28 | 0.30 | 0.30 | 0.30 |
| 835 | 98 | HG2@ | 99 | HA | 0.75 | 0.42 | 0.44 | 0.44 | 0.46 |
| 836 | 98 | HB | 99 | HN | 0.30 | 0.26 | 0.25 | 0.26 | 0.25 |
| 837 | 98 | HG2@ | 108 | HZ3 | 0.75 | 0.40 | 0.30 | 0.52 | 0.79 |
| 838 | 98 | HG2@ | 108 | HZ2 | 0.75 | 0.50 | 0.59 | 0.37 | 0.80 |
| 839 | 98 | HN | 98 | HA | 0.45 | 0.27 | 0.27 | 0.27 | 0.27 |
| 840 | 98 | HG2@ | 99 | HN | 0.75 | 0.40 | 0.40 | 0.38 | 0.40 |
| 841 | 98 | HA | 98 | HG2@ | 0.55 | 0.31 | 0.30 | 0.31 | 0.30 |
| 842 | 98 | HG2@ | 98 | HD@ | 0.90 | 0.36 | 0.35 | 0.35 | 0.35 |
| 843 | 98 | HB | 98 | HG2@ | 0.60 | 0.23 | 0.24 | 0.24 | 0.24 |
| 844 | 98 | HD@ | 108 | HZ2 | 0.60 | 0.31 | 0.44 | 0.47 | 0.74 |
| 845 | 98 | HG2@ | 108 | HE1 | 0.85 | 0.67 | 0.80 | 0.47 | 0.94 |
| 846 | 98 | HG1@ | 99 | HN | 0.75 | 0.46 | 0.46 | 0.44 | 0.46 |
| 847 | 98 | HG2@ | 99 | HB | 1.05 | 0.57 | 0.63 | 0.61 | 0.61 |
| 848 | 98 | HG2@ | 107 | HN | 0.85 | 0.51 | 0.61 | 0.79 | 0.75 |
| 849 | 98 | HG2@ | 108 | HE3 | 0.85 | 0.54 | 0.49 | 0.70 | 0.90 |
| 850 | 99 | HN | 99 | HA | 0.45 | 0.28 | 0.27 | 0.27 | 0.27 |
| 851 | 99 | HN | 99 | HB | 0.25 | 0.23 | 0.34 | 0.34 | 0.26 |
| 852 | 99 | HA | 99 | HB | 0.25 | 0.29 | 0.25 | 0.25 | 0.26 |
| 853 | 99 | HN | 99 | HG@ | 0.83 | 0.34 | 0.26 | 0.26 | 0.30 |
| 854 | 99 | HN | 100 | HN | 0.45 | 0.24 | 0.28 | 0.30 | 0.29 |
| 855 | 99 | HA | 100 | HN | 0.45 | 0.32 | 0.34 | 0.29 | 0.35 |
| 856 | 99 | HB | 100 | HN | 0.45 | 0.27 | 0.38 | 0.40 | 0.28 |
| 857 | 99 | HG@ | 108 | HZ3 | 0.83 | 0.42 | 0.73 | 0.86 | 0.69 |
| 858 | 99 | HB | 108 | HZ3 | 0.45 | 0.50 | 0.82 | 0.89 | 0.77 |
| 859 | 99 | HN | 108 | HZ3 | 0.55 | 0.47 | 0.65 | 0.82 | 0.81 |
| 860 | 100 | HN | 100 | HA | 0.30 | 0.28 | 0.26 | 0.27 | 0.26 |
| 861 | 100 | HN | 100 | HB@ | 0.65 | 0.30 | 0.28 | 0.33 | 0.30 |
| 862 | 100 | HA | 100 | HB@ | 0.65 | 0.23 | 0.25 | 0.23 | 0.25 |
| 863 | 100 | HN | 101 | HN | 0.45 | 0.26 | 0.28 | 0.24 | 0.29 |
| 864 | 101 | HN | 101 | HA | 0.45 | 0.28 | 0.28 | 0.28 | 0.28 |
| 865 | 101 | HN | 101 | HB@ | 0.45 | 0.28 | 0.23 | 0.26 | 0.26 |
| 866 | 101 | HA | 101 | HB@ | 0.45 | 0.25 | 0.26 | 0.25 | 0.26 |
| 867 | 101 | HA | 102 | HN | 0.45 | 0.31 | 0.23 | 0.23 | 0.30 |
| 868 | 102 | HN | 102 | HA@ | 0.65 | 0.25 | 0.23 | 0.24 | 0.23 |
| 869 | 104 | HN | 104 | HA@ | 0.65 | 0.24 | 0.23 | 0.23 | 0.23 |
| 870 | 104 | HN | 105 | HN | 0.45 | 0.46 | 0.46 | 0.46 | 0.29 |
| 871 | 104 | HA@ | 105 | HN | 0.65 | 0.24 | 0.25 | 0.24 | 0.30 |
| 872 | 105 | HN | 105 | HA | 0.45 | 0.27 | 0.28 | 0.27 | 0.26 |
| 873 | 105 | HN | 105 | HB@ | 0.65 | 0.30 | 0.28 | 0.25 | 0.29 |
| 874 | 105 | HA | 105 | HB@ | 0.50 | 0.26 | 0.26 | 0.26 | 0.23 |
| 875 | 105 | HA | 105 | HE@ | 0.45 | 0.45 | 0.48 | 0.50 | 0.58 |
| 876 | 105 | HB@ | 108 | HE3 | 0.65 | 0.37 | 0.39 | 0.74 | 0.62 |
| 877 | 105 | HA | 108 | HE3 | 0.25 | 0.22 | 0.26 | 0.48 | 0.60 |
| 878 | 105 | HA | 108 | HZ3 | 0.30 | 0.26 | 0.29 | 0.48 | 0.59 |
| 879 | 105 | HE@ | 105 | HB@ | 0.65 | 0.35 | 0.35 | 0.35 | 0.43 |
| 880 | 105 | HB@ | 111 | HZ2 | 0.65 | 0.43 | 0.49 | 0.66 | 0.69 |
| 881 | 105 | HB@ | 108 | HZ3 | 0.75 | 0.46 | 0.50 | 0.75 | 0.56 |
| 882 | 105 | HB@ | 111 | HE1 | 0.75 | 0.44 | 0.46 | 0.49 | 0.84 |
| 883 | 105 | HE@ | 111 | HE1 | 0.70 | 0.45 | 0.57 | 0.57 | 1.10 |
| 884 | 105 | HB@ | 106 | HN | 0.75 | 0.37 | 0.42 | 0.33 | 0.33 |
| 885 | 106 | HN | 106 | HB@ | 0.65 | 0.28 | 0.28 | 0.28 | 0.28 |
| 886 | 106 | HA | 106 | HB@ | 0.50 | 0.28 | 0.23 | 0.26 | 0.26 |
| 887 | 106 | HA | 107 | HN | 0.45 | 0.34 | 0.34 | 0.34 | 0.28 |
| 888 | 106 | HN | 107 | HN | 0.30 | 0.29 | 0.30 | 0.30 | 0.33 |
| 889 | 107 | HN | 107 | HA | 0.30 | 0.27 | 0.27 | 0.27 | 0.27 |
| 890 | 107 | HN | 107 | HB@ | 0.55 | 0.27 | 0.27 | 0.27 | 0.27 |
| 891 | 107 | HA | 107 | HB@ | 0.55 | 0.24 | 0.24 | 0.24 | 0.24 |
| 892 | 107 | HA | 108 | HN | 0.45 | 0.34 | 0.34 | 0.33 | 0.28 |
| 893 | 107 | HB@ | 108 | HN | 0.75 | 0.36 | 0.37 | 0.37 | 0.40 |
| 894 | 107 | HB@ | 108 | HE3 | 0.60 | 0.41 | 0.40 | 0.34 | 0.52 |
| 895 | 107 | HN | 108 | HN | 0.30 | 0.28 | 0.29 | 0.28 | 0.31 |
| 896 | 107 | HN | 109 | HN | 0.75 | 0.66 | 0.68 | 0.71 | 0.73 |
| 897 | 108 | HN | 108 | HB@ | 0.65 | 0.29 | 0.27 | 0.32 | 0.30 |
| 898 | 108 | HH2 | 108 | HZ2 | 0.30 | 0.25 | 0.25 | 0.25 | 0.25 |
| 899 | 108 | HE3 | 108 | HZ3 | 0.30 | 0.25 | 0.25 | 0.25 | 0.25 |
| 900 | 108 | HZ3 | 108 | HH2 | 0.30 | 0.25 | 0.25 | 0.25 | 0.25 |
| 901 | 108 | HE3 | 108 | HB@ | 0.65 | 0.33 | 0.33 | 0.33 | 0.29 |
| 902 | 108 | HA | 108 | HD1 | 0.45 | 0.31 | 0.33 | 0.45 | 0.26 |
| 903 | 108 | HN | 108 | HE3 | 0.45 | 0.26 | 0.23 | 0.34 | 0.39 |
| 904 | 108 | HN | 108 | HZ3 | 0.55 | 0.47 | 0.47 | 0.54 | 0.60 |
| 905 | 109 | HA | 109 | HB | 0.25 | 0.23 | 0.27 | 0.26 | 0.25 |
| 906 | 109 | HA | 112 | HN | 0.30 | 0.36 | 0.36 | 0.33 | 0.37 |
| 907 | 109 | HN | 109 | HB | 0.30 | 0.25 | 0.27 | 0.28 | 0.30 |
| 908 | 109 | HN | 110 | HN | 0.30 | 0.28 | 0.28 | 0.29 | 0.29 |
| 909 | 109 | HN | 111 | HN | 0.45 | 0.42 | 0.43 | 0.48 | 0.43 |
| 910 | 109 | HN | 109 | HG@ | 0.83 | 0.35 | 0.30 | 0.28 | 0.28 |
| 911 | 109 | HG@ | 110 | HN | 0.98 | 0.35 | 0.40 | 0.41 | 0.39 |
| 912 | 109 | HB | 110 | HN | 0.30 | 0.40 | 0.28 | 0.33 | 0.35 |
| 913 | 109 | HA | 110 | HN | 0.45 | 0.35 | 0.35 | 0.34 | 0.34 |
| 914 | 109 | HN | 112 | HN | 0.55 | 0.50 | 0.50 | 0.53 | 0.51 |
| 915 | 110 | HN | 110 | HA | 0.30 | 0.28 | 0.27 | 0.26 | 0.27 |
| 916 | 110 | HA | 110 | HB@ | 0.55 | 0.24 | 0.24 | 0.25 | 0.24 |
| 917 | 110 | HA | 113 | HB@ | 0.50 | 0.35 | 0.39 | 0.38 | 0.51 |
| 918 | 110 | HN | 110 | HB@ | 0.55 | 0.28 | 0.27 | 0.26 | 0.27 |
| 919 | 110 | HN | 111 | HN | 0.30 | 0.26 | 0.29 | 0.31 | 0.26 |
| 920 | 111 | HN | 111 | HA | 0.30 | 0.28 | 0.27 | 0.28 | 0.27 |
| 921 | 111 | HN | 112 | HN | 0.25 | 0.28 | 0.27 | 0.25 | 0.27 |
| 922 | 111 | HE3 | 111 | HZ3 | 0.25 | 0.25 | 0.25 | 0.25 | 0.25 |
| 923 | 111 | HZ3 | 111 | HH2 | 0.30 | 0.25 | 0.25 | 0.25 | 0.25 |
| 924 | 111 | HE3 | 112 | HA | 0.30 | 0.26 | 0.35 | 0.28 | 0.63 |
| 925 | 111 | HE3 | 112 | HB@ | 0.65 | 0.44 | 0.47 | 0.46 | 0.77 |
| 926 | 111 | HE1 | 116 | HA | 0.55 | 0.41 | 0.39 | 0.40 | 0.56 |
| 927 | 111 | HZ2 | 116 | HB@ | 0.75 | 0.44 | 0.74 | 0.55 | 0.82 |
| 928 | 112 | HN | 112 | HA | 0.30 | 0.26 | 0.26 | 0.27 | 0.27 |
| 929 | 112 | HN | 112 | HB@ | 0.45 | 0.31 | 0.28 | 0.28 | 0.29 |
| 930 | 112 | HN | 113 | HN | 0.45 | 0.28 | 0.30 | 0.28 | 0.30 |
| 931 | 112 | HB@ | 113 | HN | 0.50 | 0.32 | 0.29 | 0.30 | 0.40 |
| 932 | 112 | HA | 116 | HB@ | 0.50 | 0.27 | 0.70 | 0.55 | 0.46 |
| 933 | 112 | HN | 116 | HB@ | 0.95 | 0.51 | 0.89 | 0.75 | 0.59 |
| 934 | 113 | HN | 113 | HA | 0.45 | 0.28 | 0.27 | 0.28 | 0.27 |
| 935 | 113 | HN | 113 | HB@ | 0.45 | 0.28 | 0.27 | 0.23 | 0.35 |
| 936 | 113 | HA | 113 | HB@ | 0.45 | 0.26 | 0.26 | 0.25 | 0.22 |
| 937 | 113 | HB@ | 114 | HN | 0.50 | 0.33 | 0.30 | 0.43 | 0.29 |
| 938 | 113 | HN | 114 | HN | 0.45 | 0.22 | 0.26 | 0.40 | 0.33 |
| 939 | 114 | HN | 114 | HA | 0.30 | 0.28 | 0.27 | 0.20 | 0.26 |
| 940 | 114 | HA | 114 | HB@ | 0.50 | 0.25 | 0.25 | 0.26 | 0.24 |
| 941 | 114 | HN | 114 | HB@ | 0.50 | 0.35 | 0.30 | 0.36 | 0.28 |
| 942 | 114 | HN | 115 | HN | 0.45 | 0.23 | 0.24 | 0.33 | 0.30 |
| 943 | 115 | HN | 115 | HA | 0.45 | 0.28 | 0.27 | 0.27 | 0.28 |
| 944 | 115 | HN | 115 | HB@ | 0.65 | 0.30 | 0.28 | 0.29 | 0.29 |
| 945 | 115 | HA | 115 | HB@ | 0.50 | 0.25 | 0.25 | 0.25 | 0.25 |
| 946 | 116 | HN | 116 | HA | 0.30 | 0.27 | 0.28 | 0.21 | 0.28 |
| 947 | 116 | HN | 116 | HB@ | 0.45 | 0.23 | 0.28 | 0.34 | 0.24 |
| 948 | 116 | HA | 116 | HB@ | 0.50 | 0.25 | 0.26 | 0.26 | 0.25 |
| 949 | 116 | HA | 117 | HN | 0.25 | 0.22 | 0.25 | 0.27 | 0.22 |
| 950 | 116 | HB@ | 117 | HN | 0.65 | 0.41 | 0.33 | 0.41 | 0.39 |
| 951 | 116 | HN | 117 | HN | 0.55 | 0.46 | 0.31 | 0.32 | 0.44 |
| 952 | 117 | HN | 117 | HA@ | 0.50 | 0.24 | 0.24 | 0.23 | 0.23 |
| 953 | 117 | HN | 118 | HN | 0.30 | 0.27 | 0.36 | 0.38 | 0.33 |
| 954 | 118 | HN | 118 | HA | 0.45 | 0.29 | 0.27 | 0.27 | 0.26 |
| 955 | 118 | HN | 118 | HB | 0.45 | 0.37 | 0.35 | 0.34 | 0.35 |
| 956 | 118 | HA | 118 | HB | 0.25 | 0.24 | 0.26 | 0.26 | 0.25 |
| 957 | 118 | HA | 119 | HN | 0.30 | 0.25 | 0.29 | 0.33 | 0.22 |
| 958 | 118 | HN | 118 | HG2@ | 0.60 | 0.34 | 0.34 | 0.32 | 0.30 |
| 959 | 118 | HB | 118 | HG2@ | 0.55 | 0.23 | 0.24 | 0.24 | 0.24 |
| 960 | 118 | HG2@ | 119 | HN | 0.75 | 0.44 | 0.47 | 0.51 | 0.44 |
| 961 | 119 | HN | 119 | HA | 0.30 | 0.28 | 0.27 | 0.28 | 0.24 |
| 962 | 119 | HA | 119 | HB2 | 0.25 | 0.24 | 0.25 | 0.27 | 0.26 |
| 963 | 119 | HA | 119 | HB1 | 0.25 | 0.29 | 0.28 | 0.27 | 0.24 |
| 964 | 119 | HA | 120 | HN | 0.25 | 0.24 | 0.22 | 0.30 | 0.23 |
| 965 | 119 | HN | 119 | HB2 | 0.45 | 0.26 | 0.24 | 0.24 | 0.32 |
| 966 | 119 | HN | 119 | HB1 | 0.30 | 0.25 | 0.26 | 0.30 | 0.28 |
| 967 | 119 | HA | 121 | HN | 0.45 | 0.34 | 0.40 | 0.38 | 0.45 |
| 968 | 120 | HN | 120 | HA | 0.30 | 0.28 | 0.28 | 0.27 | 0.24 |
| 969 | 120 | HA | 120 | HB | 0.45 | 0.24 | 0.25 | 0.25 | 0.25 |
| 970 | 120 | HN | 121 | HN | 0.30 | 0.27 | 0.26 | 0.28 | 0.32 |
| 971 | 120 | HN | 120 | HG@ | 0.83 | 0.26 | 0.33 | 0.26 | 0.29 |
| 972 | 120 | HG@ | 121 | HN | 0.98 | 0.41 | 0.41 | 0.39 | 0.36 |
| 973 | 120 | HA | 123 | HE1 | 0.45 | 0.45 | 0.45 | 0.39 | 0.48 |
| 974 | 121 | HN | 121 | HA | 0.30 | 0.28 | 0.27 | 0.26 | 0.26 |
| 975 | 121 | HA | 121 | HB@ | 0.50 | 0.25 | 0.26 | 0.25 | 0.26 |
| 976 | 121 | HN | 121 | HB@ | 0.50 | 0.24 | 0.27 | 0.29 | 0.27 |
| 977 | 121 | HA | 122 | HN | 0.45 | 0.34 | 0.35 | 0.34 | 0.34 |
| 978 | 121 | HN | 122 | HN | 0.30 | 0.27 | 0.29 | 0.30 | 0.30 |
| 979 | 121 | HN | 123 | HN | 0.55 | 0.44 | 0.44 | 0.46 | 0.47 |
| 980 | 121 | HN | 123 | HD1 | 0.55 | 0.55 | 0.51 | 0.53 | 0.56 |
| 981 | 122 | HN | 122 | HA | 0.30 | 0.27 | 0.27 | 0.27 | 0.27 |
| 982 | 122 | HA | 122 | HB@ | 0.55 | 0.24 | 0.24 | 0.24 | 0.24 |
| 983 | 122 | HN | 122 | HB@ | 0.55 | 0.27 | 0.27 | 0.27 | 0.27 |
| 984 | 122 | HA | 123 | HN | 0.45 | 0.35 | 0.35 | 0.34 | 0.34 |
| 985 | 122 | HB@ | 123 | HN | 0.60 | 0.36 | 0.33 | 0.33 | 0.34 |
| 986 | 123 | HN | 123 | HA | 0.30 | 0.28 | 0.27 | 0.27 | 0.27 |
| 987 | 123 | HN | 123 | HB2 | 0.45 | 0.23 | 0.24 | 0.25 | 0.24 |
| 988 | 123 | HN | 123 | HB1 | 0.45 | 0.35 | 0.35 | 0.35 | 0.35 |
| 989 | 123 | HA | 123 | HB2 | 0.45 | 0.29 | 0.29 | 0.29 | 0.29 |
| 990 | 123 | HA | 123 | HB1 | 0.30 | 0.26 | 0.25 | 0.24 | 0.24 |
| 991 | 123 | HB1 | 124 | HN | 0.45 | 0.40 | 0.36 | 0.37 | 0.37 |
| 992 | 123 | HH2 | 123 | HZ2 | 0.25 | 0.25 | 0.25 | 0.25 | 0.25 |
| 993 | 123 | HE3 | 123 | HZ3 | 0.25 | 0.25 | 0.25 | 0.25 | 0.25 |
| 994 | 123 | HE3 | 123 | HB2 | 0.45 | 0.40 | 0.39 | 0.41 | 0.41 |
| 995 | 123 | HE3 | 123 | HB1 | 0.45 | 0.26 | 0.27 | 0.29 | 0.29 |
| 996 | 123 | HE3 | 123 | HA | 0.30 | 0.29 | 0.28 | 0.25 | 0.25 |
| 997 | 123 | HZ2 | 123 | HE3 | 0.45 | 0.50 | 0.49 | 0.49 | 0.49 |
| 998 | 123 | HE1 | 123 | HB2 | 0.55 | 0.48 | 0.47 | 0.46 | 0.47 |
| 999 | 124 | HN | 124 | HA | 0.45 | 0.28 | 0.27 | 0.28 | 0.28 |
| 1000 | 124 | HA | 124 | HB | 0.25 | 0.24 | 0.25 | 0.25 | 0.26 |
| 1001 | 124 | HN | 124 | HG1@ | 0.50 | 0.26 | 0.25 | 0.24 | 0.24 |
| 1002 | 124 | HN | 124 | HG2@ | 0.60 | 0.35 | 0.33 | 0.36 | 0.40 |
| 1003 | 124 | HN | 125 | HN | 0.25 | 0.22 | 0.29 | 0.36 | 0.36 |
| 1004 | 124 | HB | 124 | HD@ | 0.60 | 0.28 | 0.28 | 0.28 | 0.28 |
| 1005 | 124 | HB | 124 | HG2@ | 0.55 | 0.24 | 0.24 | 0.24 | 0.24 |
| 1006 | 125 | HN | 125 | HA | 0.30 | 0.28 | 0.27 | 0.27 | 0.26 |
| 1007 | 125 | HN | 125 | HB@ | 0.45 | 0.23 | 0.27 | 0.28 | 0.28 |
| 1008 | 125 | HA | 125 | HB@ | 0.50 | 0.25 | 0.26 | 0.26 | 0.26 |
| 1009 | 125 | HB@ | 126 | HN | 0.65 | 0.41 | 0.35 | 0.37 | 0.36 |
| 1010 | 125 | HA | 126 | HN | 0.25 | 0.22 | 0.23 | 0.21 | 0.21 |
| 1011 | 125 | HN | 126 | HN | 0.55 | 0.46 | 0.40 | 0.45 | 0.45 |
| 1012 | 126 | HN | 126 | HA@ | 0.50 | 0.24 | 0.24 | 0.24 | 0.24 |
| 1013 | 126 | HN | 127 | HN | 0.30 | 0.27 | 0.32 | 0.34 | 0.36 |
| 1014 | 127 | HN | 127 | HA | 0.30 | 0.28 | 0.26 | 0.28 | 0.28 |
| 1015 | 127 | HN | 127 | HB2 | 0.45 | 0.24 | 0.24 | 0.25 | 0.32 |
| 1016 | 127 | HN | 127 | HB1 | 0.45 | 0.35 | 0.35 | 0.30 | 0.27 |
| 1017 | 127 | HA | 127 | HB2 | 0.25 | 0.29 | 0.29 | 0.26 | 0.24 |
| 1018 | 127 | HA | 127 | HB1 | 0.30 | 0.24 | 0.24 | 0.24 | 0.23 |
| 1019 | 127 | HB1 | 128 | HN | 0.45 | 0.29 | 0.36 | 0.43 | 0.44 |
| 1020 | 127 | HN | 128 | HN | 0.45 | 0.46 | 0.29 | 0.33 | 0.34 |
| 1021 | 127 | HA | 128 | HN | 0.30 | 0.22 | 0.30 | 0.26 | 0.25 |
| 1022 | 127 | HB1 | 129 | HD@ | 0.98 | 0.41 | 0.76 | 0.61 | 0.62 |
| 1023 | 127 | HA | 129 | HN | 0.75 | 0.58 | 0.50 | 0.39 | 0.40 |
| 1024 | 128 | HN | 128 | HA | 0.45 | 0.28 | 0.27 | 0.27 | 0.27 |
| 1025 | 128 | HA | 128 | HB@ | 0.45 | 0.25 | 0.25 | 0.26 | 0.26 |
| 1026 | 128 | HA | 129 | HN | 0.25 | 0.23 | 0.25 | 0.26 | 0.25 |
| 1027 | 128 | HN | 129 | HB@ | 0.65 | 0.64 | 0.58 | 0.59 | 0.61 |
| 1028 | 129 | HN | 129 | HA | 0.30 | 0.29 | 0.28 | 0.27 | 0.27 |
| 1029 | 129 | HA | 129 | HB@ | 0.50 | 0.25 | 0.26 | 0.26 | 0.26 |
| 1030 | 129 | HA | 129 | HD@ | 0.78 | 0.35 | 0.35 | 0.35 | 0.35 |
| 1031 | 129 | HN | 129 | HB@ | 0.65 | 0.32 | 0.29 | 0.28 | 0.28 |
| 1032 | 129 | HN | 129 | HD@ | 0.98 | 0.46 | 0.45 | 0.40 | 0.39 |
| 1033 | 129 | HN | 129 | HG | 0.30 | 0.30 | 0.32 | 0.25 | 0.25 |
| 1034 | 129 | HB@ | 129 | HG | 0.45 | 0.25 | 0.25 | 0.25 | 0.25 |
| 1035 | 1 | HB@ | 1 | HG@ | 0.70 | 0.24 | 0.23 | 0.23 | 0.23 |
| 1036 | 1 | HB@ | 1 | HA | 0.50 | 0.25 | 0.25 | 0.25 | 0.25 |
| 1037 | 1 | HB@ | 86 | HA | 0.65 | 0.34 | 0.42 | 0.69 | 0.63 |
| 1038 | 1 | HG@ | 1 | HA | 0.50 | 0.28 | 0.27 | 0.30 | 0.28 |
| 1039 | 1 | HD@ | 1 | HE@ | 0.85 | 0.22 | 0.24 | 0.23 | 0.23 |
| 1040 | 1 | HD@ | 1 | HG@ | 0.70 | 0.24 | 0.24 | 0.23 | 0.23 |
| 1041 | 1 | HE@ | 1 | HG@ | 0.70 | 0.31 | 0.27 | 0.29 | 0.29 |
| 1042 | 1 | HE@ | 86 | HA | 0.65 | 0.64 | 0.61 | 0.78 | 0.66 |
| 1043 | 2 | HA | 39 | HB2 | 0.45 | 0.39 | 0.38 | 0.31 | 0.44 |
| 1044 | 2 | HA | 40 | HG2@ | 0.75 | 0.53 | 0.57 | 0.83 | 0.58 |
| 1045 | 2 | HG1@ | 38 | HB2 | 0.75 | 0.34 | 0.37 | 0.41 | 0.35 |
| 1046 | 2 | HG1@ | 39 | HB2 | 0.75 | 0.41 | 0.43 | 0.43 | 0.49 |
| 1047 | 2 | HG2@ | 39 | HB1 | 0.75 | 0.50 | 0.53 | 0.63 | 0.53 |
| 1048 | 2 | HG2@ | 39 | HB2 | 0.60 | 0.36 | 0.37 | 0.50 | 0.48 |
| 1049 | 2 | HG2@ | 1 | HA | 0.75 | 0.47 | 0.49 | 0.47 | 0.46 |
| 1050 | 3 | HB1 | 38 | HB1 | 0.45 | 0.34 | 0.36 | 0.50 | 0.43 |
| 1051 | 3 | HB1 | 8 | HB2 | 0.45 | 0.23 | 0.28 | 0.31 | 0.24 |
| 1052 | 3 | HB1 | 7 | HB@ | 0.65 | 0.41 | 0.47 | 0.37 | 0.42 |
| 1053 | 3 | HB2 | 8 | HB1 | 0.45 | 0.44 | 0.52 | 0.54 | 0.54 |
| 1054 | 3 | HB2 | 8 | HB2 | 0.45 | 0.29 | 0.39 | 0.39 | 0.38 |
| 1055 | 3 | HB2 | 38 | HB1 | 0.45 | 0.22 | 0.26 | 0.38 | 0.37 |
| 1056 | 4 | HA@ | 7 | HB@ | 0.85 | 0.48 | 0.44 | 0.50 | 0.51 |
| 1057 | 5 | HA | 5 | HB@ | 0.50 | 0.22 | 0.25 | 0.26 | 0.26 |
| 1058 | 5 | HA | 5 | HD@ | 0.65 | 0.46 | 0.45 | 0.43 | 0.42 |
| 1059 | 6 | HB1 | 127 | HB2 | 0.45 | 0.40 | 0.50 | 0.45 | 0.36 |
| 1060 | 6 | HB1 | 9 | HB@ | 0.75 | 0.50 | 0.55 | 0.50 | 0.52 |
| 1061 | 6 | HB2 | 9 | HB@ | 0.75 | 0.59 | 0.63 | 0.60 | 0.59 |
| 1062 | 7 | HB@ | 7 | HG@ | 0.70 | 0.22 | 0.23 | 0.24 | 0.23 |
| 1063 | 9 | HB@ | 12 | HB1 | 0.75 | 0.67 | 0.68 | 0.65 | 0.65 |
| 1064 | 12 | HA | 12 | HG@ | 0.65 | 0.25 | 0.26 | 0.26 | 0.25 |
| 1065 | 12 | HA | 12 | HB1 | 0.30 | 0.25 | 0.25 | 0.25 | 0.25 |
| 1066 | 12 | HA | 12 | HB2 | 0.30 | 0.29 | 0.29 | 0.29 | 0.29 |
| 1067 | 12 | HB2 | 17 | HD@ | 0.98 | 0.59 | 0.56 | 0.67 | 0.55 |
| 1068 | 12 | HB2 | 25 | HD@ | 0.98 | 0.38 | 0.39 | 0.37 | 0.40 |
| 1069 | 12 | HB2 | 12 | HG@ | 0.50 | 0.25 | 0.24 | 0.25 | 0.25 |
| 1070 | 12 | HG@ | 88 | HD@ | 0.95 | 0.30 | 0.42 | 0.73 | 0.49 |
| 1071 | 12 | HG@ | 17 | HD@ | 1.18 | 0.53 | 0.55 | 0.57 | 0.56 |
| 1072 | 12 | HE@ | 17 | HG | 0.45 | 0.62 | 0.61 | 0.70 | 0.58 |
| 1073 | 12 | HE@ | 28 | HA | 0.60 | 0.50 | 0.59 | 0.58 | 0.57 |
| 1074 | 12 | HE@ | 28 | HB1 | 0.60 | 0.33 | 0.37 | 0.35 | 0.34 |
| 1075 | 12 | HE@ | 28 | HB2 | 0.60 | 0.46 | 0.37 | 0.39 | 0.33 |
| 1076 | 12 | HE@ | 28 | HE3 | 0.60 | 0.44 | 0.61 | 0.33 | 0.45 |
| 1077 | 12 | HE@ | 28 | HD1 | 0.60 | 0.68 | 0.47 | 0.64 | 0.42 |
| 1078 | 13 | HA | 13 | HG@ | 0.50 | 0.27 | 0.27 | 0.26 | 0.28 |
| 1079 | 13 | HA | 13 | HD@ | 0.65 | 0.43 | 0.35 | 0.40 | 0.34 |
| 1080 | 13 | HA | 13 | HE@ | 0.65 | 0.51 | 0.48 | 0.51 | 0.49 |
| 1081 | 13 | HA | 25 | HD@ | 0.98 | 0.40 | 0.44 | 0.53 | 0.42 |
| 1082 | 14 | HA | 14 | HB@ | 0.50 | 0.26 | 0.26 | 0.25 | 0.26 |
| 1083 | 14 | HA | 14 | HD@ | 0.65 | 0.44 | 0.38 | 0.42 | 0.41 |
| 1084 | 15 | HA | 92 | HG1@ | 0.75 | 0.51 | 0.57 | 0.62 | 0.60 |
| 1085 | 15 | HB1 | 92 | HG2@ | 0.75 | 0.40 | 0.38 | 0.50 | 0.52 |
| 1086 | 15 | HB1 | 92 | HG1@ | 0.75 | 0.30 | 0.36 | 0.38 | 0.39 |
| 1087 | 17 | HA | 20 | HB2 | 0.30 | 0.22 | 0.49 | 0.55 | 0.45 |
| 1088 | 17 | HB2 | 12 | HB2 | 0.45 | 0.40 | 0.44 | 0.51 | 0.46 |
| 1089 | 17 | HD@ | 12 | HB1 | 0.98 | 0.43 | 0.41 | 0.52 | 0.41 |
| 1090 | 20 | HB1 | 17 | HD@ | 0.98 | 0.48 | 0.71 | 0.57 | 0.54 |
| 1091 | 20 | HB1 | 17 | HA | 0.45 | 0.29 | 0.54 | 0.70 | 0.53 |
| 1092 | 21 | HA | 21 | HG@ | 0.65 | 0.32 | 0.27 | 0.30 | 0.29 |
| 1093 | 21 | HA | 21 | HD@ | 0.65 | 0.25 | 0.35 | 0.42 | 0.34 |
| 1094 | 21 | HB@ | 21 | HG@ | 0.70 | 0.23 | 0.23 | 0.23 | 0.23 |
| 1095 | 21 | HB@ | 21 | HD@ | 0.85 | 0.30 | 0.28 | 0.28 | 0.29 |
| 1096 | 24 | HA | 24 | HB1 | 0.25 | 0.25 | 0.28 | 0.27 | 0.27 |
| 1097 | 24 | HA | 24 | HB2 | 0.25 | 0.24 | 0.24 | 0.24 | 0.24 |
| 1098 | 24 | HA | 19 | HB@ | 0.65 | 0.44 | 0.53 | 0.47 | 0.53 |
| 1099 | 25 | HA | 28 | HB@ | 0.65 | 0.38 | 0.41 | 0.35 | 0.39 |
| 1100 | 25 | HA | 28 | HD1 | 0.30 | 0.23 | 0.24 | 0.33 | 0.26 |
| 1101 | 25 | HB@ | 18 | HA | 0.65 | 0.34 | 0.57 | 0.55 | 0.69 |
| 1102 | 25 | HB@ | 18 | HB1 | 0.65 | 0.46 | 0.65 | 0.71 | 0.92 |
| 1103 | 25 | HB@ | 18 | HB2 | 0.65 | 0.32 | 0.53 | 0.76 | 0.91 |
| 1104 | 25 | HD@ | 18 | HB1 | 0.98 | 0.49 | 0.56 | 0.65 | 0.76 |
| 1105 | 25 | HD@ | 18 | HB2 | 0.98 | 0.33 | 0.53 | 0.67 | 0.73 |
| 1106 | 25 | HD@ | 13 | HB@ | 1.18 | 0.36 | 0.44 | 0.60 | 0.42 |
| 1107 | 25 | HD@ | 17 | HD@ | 1.51 | 0.78 | 0.64 | 0.65 | 0.60 |
| 1108 | 25 | HD@ | 9 | HA | 0.98 | 0.36 | 0.38 | 0.41 | 0.41 |
| 1109 | 27 | HB1 | 105 | HE@ | 0.60 | 0.36 | 0.48 | 0.38 | 1.07 |
| 1110 | 27 | HB2 | 105 | HE@ | 0.60 | 0.49 | 0.37 | 0.52 | 1.08 |
| 1111 | 28 | HA | 31 | HB@ | 0.60 | 0.35 | 0.35 | 0.36 | 0.36 |
| 1112 | 28 | HA | 105 | HE@ | 0.45 | 0.32 | 0.41 | 0.35 | 1.23 |
| 1113 | 28 | HA | 28 | HD1 | 0.45 | 0.44 | 0.45 | 0.31 | 0.41 |
| 1114 | 28 | HB@ | 17 | HD@ | 1.18 | 0.47 | 0.55 | 0.79 | 0.63 |
| 1115 | 29 | HA | 8 | HD@ | 0.98 | 0.47 | 0.52 | 0.43 | 0.48 |
| 1116 | 29 | HG2@ | 28 | HD1 | 0.75 | 0.64 | 0.61 | 0.71 | 0.64 |
| 1117 | 29 | HG2@ | 28 | HB2 | 0.75 | 0.44 | 0.44 | 0.42 | 0.44 |
| 1118 | 29 | HG2@ | 8 | HD@ | 1.28 | 0.56 | 0.58 | 0.54 | 0.51 |
| 1119 | 29 | HG1@ | 8 | HD@ | 1.28 | 0.59 | 0.61 | 0.53 | 0.64 |
| 1120 | 29 | HG1@ | 123 | HA | 0.75 | 0.50 | 0.59 | 0.79 | 0.60 |
| 1121 | 29 | HG1@ | 30 | HA | 0.75 | 0.46 | 0.50 | 0.49 | 0.48 |
| 1122 | 29 | HG1@ | 123 | HD1 | 0.75 | 0.64 | 0.54 | 0.86 | 0.75 |
| 1123 | 31 | HA | 111 | HB2 | 0.45 | 0.33 | 0.53 | 0.61 | 0.59 |
| 1124 | 31 | HB@ | 111 | HB1 | 0.75 | 0.46 | 0.60 | 0.68 | 0.63 |
| 1125 | 31 | HB@ | 111 | HB2 | 0.75 | 0.31 | 0.46 | 0.53 | 0.53 |
| 1126 | 31 | HB@ | 105 | HE@ | 0.90 | 0.32 | 0.38 | 0.40 | 1.30 |
| 1127 | 31 | HB@ | 56 | HD@ | 1.28 | 0.48 | 0.58 | 0.49 | 0.80 |
| 1128 | 33 | HA | 37 | HA | 0.45 | 0.30 | 0.29 | 0.35 | 0.33 |
| 1129 | 33 | HA | 33 | HB1 | 0.30 | 0.29 | 0.29 | 0.27 | 0.28 |
| 1130 | 33 | HA | 33 | HB2 | 0.30 | 0.23 | 0.24 | 0.25 | 0.25 |
| 1131 | 33 | HA | 33 | HG@ | 0.65 | 0.34 | 0.28 | 0.27 | 0.27 |
| 1132 | 33 | HB1 | 33 | HG@ | 0.50 | 0.22 | 0.24 | 0.25 | 0.25 |
| 1133 | 33 | HB2 | 33 | HG@ | 0.50 | 0.25 | 0.25 | 0.25 | 0.25 |
| 1134 | 33 | HG@ | 33 | HD@ | 0.70 | 0.24 | 0.24 | 0.23 | 0.23 |
| 1135 | 33 | HD@ | 33 | HE@ | 0.70 | 0.24 | 0.23 | 0.23 | 0.24 |
| 1136 | 35 | HA | 35 | HG@ | 0.65 | 0.31 | 0.26 | 0.25 | 0.25 |
| 1137 | 36 | HB1 | 42 | HB@ | 0.75 | 0.35 | 0.38 | 0.49 | 0.39 |
| 1138 | 36 | HB2 | 42 | HB@ | 0.75 | 0.28 | 0.32 | 0.51 | 0.36 |
| 1139 | 38 | HB1 | 2 | HG1@ | 0.75 | 0.43 | 0.43 | 0.39 | 0.38 |
| 1140 | 38 | HB2 | 3 | HB2 | 0.45 | 0.38 | 0.41 | 0.51 | 0.53 |
| 1141 | 38 | HB2 | 55 | HD@ | 0.75 | 0.55 | 0.73 | 1.10 | 0.59 |
| 1142 | 40 | HA | 55 | HD@ | 0.75 | 0.43 | 0.50 | 0.72 | 0.38 |
| 1143 | 40 | HA | 55 | HG2@ | 0.75 | 0.44 | 0.39 | 0.48 | 0.42 |
| 1144 | 40 | HG2@ | 86 | HA | 0.60 | 0.35 | 0.34 | 0.36 | 0.38 |
| 1145 | 41 | HA | 41 | HB1 | 0.30 | 0.25 | 0.25 | 0.25 | 0.26 |
| 1146 | 41 | HA | 41 | HB2 | 0.45 | 0.29 | 0.28 | 0.29 | 0.29 |
| 1147 | 41 | HA | 84 | HA | 0.45 | 0.33 | 0.40 | 0.32 | 0.31 |
| 1148 | 41 | HA | 84 | HD@ | 0.98 | 0.38 | 0.43 | 0.43 | 0.45 |
| 1149 | 41 | HB1 | 84 | HD@ | 0.98 | 0.51 | 0.59 | 0.52 | 0.56 |
| 1150 | 43 | HA | 53 | HB1 | 0.45 | 0.53 | 0.58 | 0.60 | 0.55 |
| 1151 | 43 | HG2@ | 53 | HA | 0.75 | 0.40 | 0.37 | 0.50 | 0.38 |
| 1152 | 43 | HB | 51 | HG2@ | 0.60 | 0.32 | 0.36 | 0.40 | 0.80 |
| 1153 | 45 | HA | 51 | HB | 0.45 | 0.45 | 0.51 | 0.52 | 0.58 |
| 1154 | 51 | HA | 45 | HB@ | 0.65 | 0.51 | 0.51 | 0.53 | 0.44 |
| 1155 | 51 | HG2@ | 45 | HB@ | 0.95 | 0.53 | 0.57 | 0.66 | 0.57 |
| 1156 | 55 | HD@ | 38 | HB1 | 0.75 | 0.42 | 0.58 | 1.01 | 0.48 |
| 1157 | 55 | HD@ | 8 | HD@ | 1.28 | 0.33 | 0.40 | 0.47 | 0.44 |
| 1158 | 55 | HG2@ | 8 | HD@ | 1.28 | 0.54 | 0.46 | 0.42 | 0.57 |
| 1159 | 55 | HD@ | 56 | HD@ | 1.28 | 0.74 | 0.60 | 0.66 | 0.68 |
| 1160 | 55 | HG2@ | 91 | HB@ | 0.95 | 0.35 | 0.54 | 0.53 | 0.62 |
| 1161 | 57 | HA | 57 | HB@ | 0.65 | 0.25 | 0.25 | 0.26 | 0.26 |
| 1162 | 57 | HA | 57 | HG@ | 0.65 | 0.31 | 0.30 | 0.30 | 0.31 |
| 1163 | 66 | HA | 80 | HB2 | 0.45 | 0.35 | 0.35 | 0.32 | 0.47 |
| 1164 | 66 | HB1 | 80 | HB2 | 0.45 | 0.26 | 0.45 | 0.39 | 0.48 |
| 1165 | 66 | HB2 | 80 | HB2 | 0.45 | 0.36 | 0.36 | 0.29 | 0.34 |
| 1166 | 70 | HA | 70 | HB@ | 0.50 | 0.25 | 0.24 | 0.25 | 0.25 |
| 1167 | 76 | HB@ | 78 | HA | 0.65 | 0.57 | 0.58 | 0.59 | 0.56 |
| 1168 | 78 | HA | 79 | HD@ | 0.50 | 0.23 | 0.22 | 0.21 | 0.22 |
| 1169 | 78 | HB | 79 | HD@ | 0.50 | 0.30 | 0.34 | 0.42 | 0.43 |
| 1170 | 78 | HG2@ | 64 | HB1 | 0.75 | 0.63 | 0.71 | 0.55 | 0.42 |
| 1171 | 78 | HG2@ | 64 | HB2 | 0.75 | 0.62 | 0.68 | 0.60 | 0.43 |
| 1172 | 78 | HG2@ | 79 | HD@ | 0.95 | 0.41 | 0.44 | 0.47 | 0.46 |
| 1173 | 78 | HG1@ | 79 | HD@ | 0.85 | 0.50 | 0.53 | 0.42 | 0.41 |
| 1174 | 78 | HD@ | 79 | HD@ | 0.95 | 0.59 | 0.59 | 0.45 | 0.43 |
| 1175 | 81 | HA | 84 | HB1 | 0.45 | 0.48 | 0.47 | 0.38 | 0.61 |
| 1176 | 81 | HA | 84 | HB2 | 0.45 | 0.34 | 0.33 | 0.34 | 0.47 |
| 1177 | 86 | HB@ | 40 | HG2@ | 0.95 | 0.56 | 0.55 | 0.54 | 0.48 |
| 1178 | 86 | HB@ | 1 | HG@ | 0.85 | 0.56 | 0.51 | 0.54 | 0.62 |
| 1179 | 86 | HB@ | 1 | HD@ | 0.85 | 0.39 | 0.41 | 0.59 | 0.53 |
| 1180 | 92 | HA | 91 | HA | 0.45 | 0.46 | 0.46 | 0.46 | 0.46 |
| 1181 | 92 | HG1@ | 93 | HA | 0.75 | 0.41 | 0.48 | 0.47 | 0.47 |
| 1182 | 92 | HG1@ | 95 | HB@ | 0.90 | 0.56 | 0.57 | 0.54 | 0.61 |
| 1183 | 92 | HG2@ | 95 | HB@ | 0.90 | 0.60 | 0.59 | 0.56 | 0.51 |
| 1184 | 93 | HA | 96 | HD@ | 0.65 | 0.35 | 0.37 | 0.38 | 0.43 |
| 1185 | 93 | HB1 | 90 | HA | 0.45 | 0.45 | 0.38 | 0.39 | 0.42 |
| 1186 | 93 | HB2 | 90 | HA | 0.45 | 0.29 | 0.29 | 0.28 | 0.28 |
| 1187 | 94 | HA | 97 | HB@ | 0.65 | 0.28 | 0.35 | 0.38 | 0.37 |
| 1188 | 96 | HA | 96 | HG@ | 0.65 | 0.26 | 0.26 | 0.27 | 0.28 |
| 1189 | 96 | HA | 96 | HD@ | 0.65 | 0.44 | 0.43 | 0.41 | 0.34 |
| 1190 | 96 | HB@ | 96 | HG@ | 0.65 | 0.23 | 0.24 | 0.24 | 0.23 |
| 1191 | 96 | HG@ | 96 | HE@ | 0.70 | 0.25 | 0.28 | 0.29 | 0.29 |
| 1192 | 96 | HG@ | 96 | HD@ | 0.70 | 0.24 | 0.23 | 0.23 | 0.23 |
| 1193 | 96 | HD@ | 96 | HE@ | 0.70 | 0.24 | 0.24 | 0.23 | 0.23 |
| 1194 | 96 | HD@ | 17 | HD@ | 1.18 | 0.63 | 0.62 | 0.46 | 0.49 |
| 1195 | 96 | HE@ | 17 | HD@ | 1.18 | 0.47 | 0.59 | 0.46 | 0.54 |
| 1196 | 98 | HA | 95 | HA | 0.45 | 0.54 | 0.54 | 0.53 | 0.53 |
| 1197 | 98 | HA | 95 | HB@ | 0.75 | 0.76 | 0.76 | 0.76 | 0.76 |
| 1198 | 98 | HB | 95 | HA | 0.45 | 0.30 | 0.29 | 0.30 | 0.28 |
| 1199 | 98 | HD@ | 95 | HA | 0.75 | 0.33 | 0.32 | 0.34 | 0.31 |
| 1200 | 98 | HG2@ | 107 | HB@ | 1.05 | 0.32 | 0.69 | 0.78 | 0.81 |
| 1201 | 98 | HD@ | 58 | HD@ | 1.05 | 0.40 | 0.42 | 0.52 | 0.48 |
| 1202 | 108 | HB@ | 111 | HB1 | 0.65 | 0.28 | 0.27 | 0.60 | 0.43 |
| 1203 | 108 | HB@ | 111 | HB2 | 0.65 | 0.29 | 0.30 | 0.57 | 0.28 |
| 1204 | 111 | HA | 115 | HB@ | 0.65 | 0.34 | 0.28 | 0.32 | 0.32 |
| 1205 | 113 | HB@ | 109 | HG@ | 1.18 | 0.58 | 0.67 | 0.50 | 0.86 |
| 1206 | 116 | HB@ | 116 | HG@ | 0.65 | 0.22 | 0.23 | 0.24 | 0.24 |
| 1207 | 116 | HG@ | 116 | HD@ | 0.65 | 0.24 | 0.24 | 0.23 | 0.23 |
| 1208 | 118 | HA | 118 | HG2@ | 0.60 | 0.30 | 0.28 | 0.29 | 0.29 |
| 1209 | 121 | HA | 124 | HD@ | 0.75 | 0.47 | 0.39 | 0.46 | 0.50 |
| 1210 | 121 | HA | 124 | HG1@ | 0.65 | 0.31 | 0.29 | 0.35 | 0.39 |
| 1211 | 123 | HB@ | 29 | HG1@ | 0.95 | 0.36 | 0.40 | 0.64 | 0.48 |
| 1212 | 123 | HB@ | 29 | HG2@ | 0.95 | 0.51 | 0.47 | 0.84 | 0.63 |
| 1213 | 124 | HG2@ | 26 | HA@ | 0.95 | 0.35 | 0.74 | 0.52 | 0.41 |
| 1214 | 124 | HD@ | 26 | HA@ | 0.95 | 0.52 | 0.81 | 0.32 | 0.41 |
| 1215 | 124 | HD@ | 121 | HG@ | 0.95 | 0.45 | 0.55 | 0.71 | 0.62 |
| 1216 | 2 | HN | 39 | HA | 0.55 | 0.48 | 0.47 | 0.64 | 0.49 |
| 1217 | 3 | HN | 8 | HD@ | 1.08 | 0.60 | 0.64 | 0.62 | 0.65 |
| 1218 | 3 | HN | 38 | HB2 | 0.45 | 0.33 | 0.34 | 0.42 | 0.40 |
| 1219 | 3 | HN | 39 | HN | 0.55 | 0.50 | 0.49 | 0.48 | 0.50 |
| 1220 | 3 | HN | 40 | HB | 0.55 | 0.69 | 0.75 | 0.91 | 0.69 |
| 1221 | 4 | HN | 38 | HB2 | 0.55 | 0.59 | 0.58 | 0.57 | 0.47 |
| 1222 | 5 | HN | 38 | HD@ | 0.89 | 0.55 | 0.54 | 0.43 | 0.34 |
| 1223 | 8 | HN | 3 | HE@ | 0.89 | 0.60 | 0.68 | 0.74 | 0.64 |
| 1224 | 9 | HN | 124 | HG2@ | 0.75 | 0.57 | 0.54 | 0.69 | 0.48 |
| 1225 | 11 | HN | 129 | HD@ | 1.08 | 0.71 | 1.21 | 0.92 | 0.85 |
| 1226 | 12 | HN | 25 | HD@ | 0.98 | 0.54 | 0.57 | 0.58 | 0.59 |
| 1227 | 14 | HN | 25 | HD@ | 0.98 | 0.62 | 0.67 | 0.78 | 0.67 |
| 1228 | 17 | HN | 12 | HB2 | 0.45 | 0.52 | 0.54 | 0.59 | 0.56 |
| 1229 | 17 | HN | 12 | HA | 0.75 | 0.42 | 0.43 | 0.45 | 0.45 |
| 1230 | 17 | HN | 25 | HD@ | 1.08 | 0.59 | 0.61 | 0.70 | 0.64 |
| 1231 | 19 | HN | 25 | HB@ | 0.65 | 0.48 | 0.69 | 0.52 | 0.65 |
| 1232 | 19 | HN | 28 | HD1 | 0.75 | 0.38 | 0.74 | 0.86 | 0.74 |
| 1233 | 20 | HN | 28 | HZ2 | 0.45 | 0.49 | 0.72 | 0.56 | 0.77 |
| 1234 | 24 | HN | 19 | HA | 0.45 | 0.41 | 0.52 | 0.67 | 0.59 |
| 1235 | 27 | HN | 120 | HB | 0.45 | 0.36 | 0.62 | 0.48 | 0.50 |
| 1236 | 28 | HN | 105 | HE@ | 0.60 | 0.50 | 0.53 | 0.47 | 1.26 |
| 1237 | 31 | HN | 56 | HD@ | 0.98 | 0.65 | 0.70 | 0.65 | 0.88 |
| 1238 | 31 | HN | 105 | HE@ | 0.60 | 0.46 | 0.51 | 0.53 | 1.40 |
| 1239 | 31 | HN | 111 | HB1 | 0.55 | 0.63 | 0.71 | 0.80 | 0.76 |
| 1240 | 31 | HN | 111 | HB2 | 0.55 | 0.47 | 0.56 | 0.67 | 0.69 |
| 1241 | 39 | HN | 2 | HA | 0.55 | 0.46 | 0.46 | 0.51 | 0.47 |
| 1242 | 39 | HN | 2 | HG1@ | 0.85 | 0.51 | 0.53 | 0.57 | 0.54 |
| 1243 | 39 | HN | 2 | HG2@ | 0.85 | 0.57 | 0.54 | 0.70 | 0.61 |
| 1244 | 39 | HN | 55 | HG2@ | 0.85 | 0.70 | 0.51 | 0.59 | 0.79 |
| 1245 | 39 | HN | 55 | HD@ | 0.85 | 0.52 | 0.66 | 0.91 | 0.46 |
| 1246 | 40 | HN | 55 | HD@ | 0.75 | 0.48 | 0.59 | 0.76 | 0.48 |
| 1247 | 42 | HN | 54 | HN | 0.75 | 0.41 | 0.47 | 0.50 | 0.52 |
| 1248 | 45 | HN | 51 | HG2@ | 0.85 | 0.51 | 0.58 | 0.65 | 0.74 |
| 1249 | 46 | HN | 51 | HG2@ | 0.85 | 0.51 | 0.55 | 0.59 | 0.65 |
| 1250 | 54 | HN | 42 | HB@ | 0.85 | 0.44 | 0.56 | 0.68 | 0.56 |
| 1251 | 59 | HN | 98 | HD@ | 0.85 | 0.52 | 0.59 | 0.42 | 0.62 |
| 1252 | 60 | HN | 51 | HB | 0.55 | 0.46 | 0.46 | 0.51 | 0.36 |
| 1253 | 61 | HN | 50 | HB1 | 0.45 | 0.40 | 0.36 | 0.33 | 0.56 |
| 1254 | 61 | HN | 50 | HB2 | 0.45 | 0.31 | 0.27 | 0.34 | 0.51 |
| 1255 | 63 | HN | 58 | HG2@ | 0.75 | 0.51 | 0.48 | 0.55 | 0.65 |
| 1256 | 64 | HN | 58 | HG2@ | 0.75 | 0.46 | 0.39 | 0.40 | 0.56 |
| 1257 | 64 | HN | 74 | HA | 0.55 | 0.39 | 0.52 | 0.43 | 0.50 |
| 1258 | 65 | HN | 78 | HG1@ | 0.75 | 0.44 | 0.48 | 0.76 | 0.63 |
| 1259 | 65 | HN | 79 | HA | 0.45 | 0.28 | 0.31 | 0.28 | 0.30 |
| 1260 | 65 | HN | 74 | HD@ | 0.75 | 0.40 | 0.67 | 0.44 | 0.58 |
| 1261 | 80 | HN | 66 | HB@ | 0.65 | 0.42 | 0.49 | 0.49 | 0.47 |
| 1262 | 86 | HN | 40 | HG2@ | 0.75 | 0.61 | 0.53 | 0.60 | 0.46 |
| 1263 | 88 | HN | 3 | HE@ | 0.99 | 0.50 | 0.52 | 0.58 | 0.60 |
| 1264 | 88 | HN | 3 | HZ | 0.45 | 0.40 | 0.43 | 0.50 | 0.54 |
| 1265 | 91 | HN | 84 | HD@ | 1.08 | 1.01 | 1.13 | 1.27 | 1.36 |
| 1266 | 92 | HN | 17 | HD@ | 1.08 | 0.66 | 0.67 | 0.89 | 0.73 |
| 1267 | 95 | HN | 17 | HD@ | 1.08 | 0.63 | 0.59 | 0.83 | 0.73 |
| 1268 | 98 | HN | 108 | HZ2 | 0.55 | 0.55 | 0.81 | 0.74 | 0.99 |
| 1269 | 99 | HN | 20 | HE@ | 0.99 | 0.60 | 0.70 | 0.55 | 0.65 |
| 1270 | 100 | HN | 20 | HE@ | 0.99 | 0.50 | 0.53 | 0.49 | 0.46 |
| 1271 | 103 | HN | 98 | HG2@ | 0.85 | 0.51 | 0.71 | 0.57 | 0.41 |
| 1272 | 104 | HN | 99 | HA | 0.45 | 0.53 | 0.40 | 0.47 | 0.61 |
| 1273 | 104 | HN | 99 | HG@ | 0.98 | 0.64 | 0.68 | 0.74 | 0.84 |
| 1274 | 111 | HN | 31 | HB@ | 0.75 | 0.49 | 0.51 | 0.53 | 0.53 |
| 1275 | 116 | HN | 111 | HA | 0.45 | 0.41 | 0.50 | 0.36 | 0.35 |
| 1276 | 127 | HN | 6 | HB1 | 0.55 | 0.54 | 0.61 | 0.59 | 0.52 |
| 1277 | 128 | HN | 6 | HB1 | 0.45 | 0.35 | 0.68 | 0.77 | 0.75 |
| 1278 | 128 | HN | 6 | HB2 | 0.55 | 0.48 | 0.63 | 0.81 | 0.82 |
| 1279 | 129 | HN | 6 | HB1 | 0.55 | 0.71 | 0.87 | 0.75 | 0.72 |
| 1280 | 2 | HN | 1 | HB@ | 0.65 | 0.40 | 0.37 | 0.33 | 0.34 |
| 1281 | 2 | HN | 1 | HG@ | 0.65 | 0.28 | 0.34 | 0.37 | 0.37 |
| 1282 | 3 | HN | 3 | HE@ | 0.99 | 0.56 | 0.57 | 0.52 | 0.58 |
| 1283 | 3 | HN | 4 | HN | 0.45 | 0.47 | 0.45 | 0.35 | 0.35 |
| 1284 | 4 | HN | 3 | HB1 | 0.45 | 0.29 | 0.26 | 0.42 | 0.42 |
| 1285 | 4 | HN | 3 | HB2 | 0.45 | 0.41 | 0.38 | 0.43 | 0.44 |
| 1286 | 4 | HN | 5 | HA | 0.55 | 0.55 | 0.54 | 0.60 | 0.61 |
| 1287 | 4 | HN | 7 | HG@ | 0.65 | 0.44 | 0.42 | 0.49 | 0.50 |
| 1288 | 4 | HN | 7 | HN | 0.45 | 0.42 | 0.46 | 0.48 | 0.50 |
| 1289 | 4 | HN | 3 | HA | 0.45 | 0.23 | 0.23 | 0.25 | 0.24 |
| 1290 | 5 | HN | 5 | HB@ | 0.50 | 0.30 | 0.27 | 0.35 | 0.32 |
| 1291 | 5 | HN | 5 | HG@ | 0.65 | 0.28 | 0.29 | 0.32 | 0.36 |
| 1292 | 6 | HN | 4 | HA@ | 0.75 | 0.41 | 0.43 | 0.52 | 0.51 |
| 1293 | 6 | HN | 5 | HB@ | 0.50 | 0.38 | 0.30 | 0.22 | 0.24 |
| 1294 | 6 | HN | 5 | HG@ | 0.65 | 0.25 | 0.36 | 0.40 | 0.37 |
| 1295 | 6 | HN | 9 | HN | 0.55 | 0.46 | 0.48 | 0.51 | 0.49 |
| 1296 | 7 | HN | 3 | HD@ | 0.99 | 0.64 | 0.75 | 0.71 | 0.67 |
| 1297 | 7 | HN | 4 | HA@ | 0.65 | 0.41 | 0.43 | 0.47 | 0.48 |
| 1298 | 7 | HN | 6 | HA | 0.45 | 0.35 | 0.35 | 0.34 | 0.34 |
| 1299 | 7 | HN | 7 | HG@ | 0.65 | 0.34 | 0.33 | 0.30 | 0.30 |
| 1300 | 8 | HN | 5 | HA | 0.55 | 0.36 | 0.38 | 0.44 | 0.44 |
| 1301 | 8 | HN | 7 | HA | 0.45 | 0.35 | 0.35 | 0.35 | 0.35 |
| 1302 | 8 | HN | 7 | HG@ | 0.65 | 0.49 | 0.41 | 0.47 | 0.45 |
| 1303 | 9 | HN | 6 | HB1 | 0.45 | 0.51 | 0.55 | 0.52 | 0.54 |
| 1304 | 9 | HN | 6 | HB2 | 0.55 | 0.54 | 0.57 | 0.57 | 0.57 |
| 1305 | 9 | HN | 7 | HA | 0.45 | 0.45 | 0.47 | 0.45 | 0.46 |
| 1306 | 9 | HN | 12 | HN | 0.45 | 0.48 | 0.48 | 0.50 | 0.48 |
| 1307 | 9 | HN | 5 | HA | 0.45 | 0.39 | 0.41 | 0.48 | 0.44 |
| 1308 | 10 | HN | 8 | HA | 0.55 | 0.43 | 0.47 | 0.45 | 0.46 |
| 1309 | 11 | HN | 9 | HA | 0.45 | 0.46 | 0.47 | 0.44 | 0.46 |
| 1310 | 11 | HN | 12 | HG@ | 0.75 | 0.55 | 0.55 | 0.59 | 0.56 |
| 1311 | 11 | HN | 8 | HD@ | 0.98 | 0.63 | 0.69 | 0.66 | 0.67 |
| 1312 | 12 | HN | 8 | HA | 0.55 | 0.38 | 0.39 | 0.42 | 0.39 |
| 1313 | 12 | HN | 12 | HG@ | 0.50 | 0.30 | 0.28 | 0.31 | 0.29 |
| 1314 | 13 | HN | 9 | HA | 0.45 | 0.41 | 0.40 | 0.41 | 0.40 |
| 1315 | 13 | HN | 11 | HA | 0.45 | 0.43 | 0.46 | 0.46 | 0.46 |
| 1316 | 13 | HN | 12 | HG@ | 0.65 | 0.47 | 0.46 | 0.48 | 0.46 |
| 1317 | 13 | HN | 15 | HN | 0.45 | 0.40 | 0.44 | 0.44 | 0.45 |
| 1318 | 13 | HN | 16 | HN | 0.55 | 0.51 | 0.52 | 0.57 | 0.56 |
| 1319 | 14 | HN | 12 | HA | 0.55 | 0.45 | 0.46 | 0.44 | 0.46 |
| 1320 | 14 | HN | 13 | HG@ | 0.65 | 0.42 | 0.42 | 0.45 | 0.41 |
| 1321 | 14 | HN | 16 | HN | 0.45 | 0.41 | 0.43 | 0.45 | 0.45 |
| 1322 | 14 | HN | 15 | HB2 | 0.45 | 0.51 | 0.50 | 0.51 | 0.51 |
| 1323 | 15 | HN | 12 | HA | 0.45 | 0.34 | 0.35 | 0.35 | 0.37 |
| 1324 | 15 | HN | 13 | HA | 0.45 | 0.41 | 0.45 | 0.41 | 0.44 |
| 1325 | 15 | HN | 14 | HA | 0.45 | 0.34 | 0.35 | 0.34 | 0.35 |
| 1326 | 15 | HN | 14 | HB@ | 0.65 | 0.33 | 0.31 | 0.32 | 0.31 |
| 1327 | 15 | HN | 17 | HD@ | 1.28 | 0.70 | 0.80 | 0.76 | 0.71 |
| 1328 | 16 | HN | 15 | HA | 0.45 | 0.31 | 0.30 | 0.28 | 0.29 |
| 1329 | 16 | HN | 18 | HN | 0.45 | 0.41 | 0.53 | 0.52 | 0.51 |
| 1330 | 16 | HN | 17 | HG | 0.45 | 0.49 | 0.62 | 0.62 | 0.64 |
| 1331 | 16 | HN | 15 | HB1 | 0.45 | 0.44 | 0.41 | 0.41 | 0.43 |
| 1332 | 16 | HN | 15 | HB2 | 0.45 | 0.39 | 0.38 | 0.42 | 0.39 |
| 1333 | 17 | HN | 16 | HA@ | 0.65 | 0.32 | 0.30 | 0.31 | 0.31 |
| 1334 | 17 | HN | 18 | HB1 | 0.45 | 0.51 | 0.54 | 0.64 | 0.63 |
| 1335 | 17 | HN | 18 | HB2 | 0.55 | 0.50 | 0.53 | 0.56 | 0.57 |
| 1336 | 18 | HN | 19 | HN | 0.45 | 0.45 | 0.44 | 0.30 | 0.34 |
| 1337 | 18 | HN | 20 | HN | 0.55 | 0.43 | 0.54 | 0.70 | 0.57 |
| 1338 | 20 | HN | 17 | HD@ | 0.98 | 0.62 | 0.74 | 0.64 | 0.63 |
| 1339 | 20 | HN | 19 | HA | 0.30 | 0.30 | 0.23 | 0.26 | 0.24 |
| 1340 | 20 | HN | 19 | HB@ | 0.65 | 0.42 | 0.39 | 0.31 | 0.36 |
| 1341 | 20 | HN | 23 | HA | 0.45 | 0.57 | 0.54 | 0.63 | 0.67 |
| 1342 | 21 | HN | 20 | HB1 | 0.45 | 0.42 | 0.32 | 0.35 | 0.36 |
| 1343 | 21 | HN | 20 | HB2 | 0.45 | 0.42 | 0.38 | 0.39 | 0.31 |
| 1344 | 21 | HN | 21 | HB@ | 0.65 | 0.36 | 0.32 | 0.29 | 0.28 |
| 1345 | 21 | HN | 21 | HG@ | 0.75 | 0.35 | 0.31 | 0.27 | 0.29 |
| 1346 | 22 | HN | 19 | HA | 0.55 | 0.37 | 0.51 | 0.79 | 0.65 |
| 1347 | 22 | HN | 20 | HA | 0.45 | 0.36 | 0.42 | 0.56 | 0.50 |
| 1348 | 22 | HN | 20 | HN | 0.55 | 0.44 | 0.47 | 0.55 | 0.52 |
| 1349 | 22 | HN | 21 | HB@ | 0.65 | 0.42 | 0.42 | 0.37 | 0.39 |
| 1350 | 23 | HN | 19 | HA | 0.45 | 0.32 | 0.47 | 0.80 | 0.70 |
| 1351 | 23 | HN | 20 | HN | 0.55 | 0.37 | 0.38 | 0.57 | 0.57 |
| 1352 | 23 | HN | 22 | HA@ | 0.65 | 0.32 | 0.31 | 0.29 | 0.29 |
| 1353 | 23 | HN | 21 | HB@ | 0.75 | 0.45 | 0.44 | 0.51 | 0.51 |
| 1354 | 24 | HN | 23 | HB1 | 0.45 | 0.36 | 0.28 | 0.40 | 0.41 |
| 1355 | 24 | HN | 23 | HB2 | 0.45 | 0.43 | 0.39 | 0.44 | 0.44 |
| 1356 | 24 | HN | 24 | HB1 | 0.30 | 0.25 | 0.30 | 0.25 | 0.25 |
| 1357 | 24 | HN | 24 | HB2 | 0.45 | 0.35 | 0.32 | 0.28 | 0.29 |
| 1358 | 24 | HN | 25 | HN | 0.45 | 0.46 | 0.42 | 0.46 | 0.45 |
| 1359 | 24 | HN | 27 | HB1 | 0.55 | 0.48 | 0.45 | 0.44 | 0.43 |
| 1360 | 24 | HN | 27 | HB2 | 0.55 | 0.35 | 0.50 | 0.38 | 0.31 |
| 1361 | 24 | HN | 27 | HD@ | 0.65 | 0.27 | 0.39 | 0.50 | 0.46 |
| 1362 | 24 | HN | 27 | HN | 0.55 | 0.41 | 0.38 | 0.44 | 0.37 |
| 1363 | 25 | HN | 24 | HA | 0.30 | 0.23 | 0.26 | 0.24 | 0.26 |
| 1364 | 26 | HN | 27 | HD@ | 0.75 | 0.55 | 0.63 | 0.69 | 0.63 |
| 1365 | 26 | HN | 28 | HD1 | 0.55 | 0.50 | 0.51 | 0.55 | 0.50 |
| 1366 | 27 | HN | 25 | HA | 0.45 | 0.43 | 0.45 | 0.41 | 0.44 |
| 1367 | 27 | HN | 26 | HA@ | 0.65 | 0.31 | 0.30 | 0.31 | 0.31 |
| 1368 | 27 | HN | 27 | HD@ | 0.65 | 0.34 | 0.42 | 0.43 | 0.40 |
| 1369 | 27 | HN | 29 | HN | 0.45 | 0.44 | 0.45 | 0.44 | 0.43 |
| 1370 | 27 | HN | 28 | HD1 | 0.45 | 0.45 | 0.49 | 0.40 | 0.42 |
| 1371 | 28 | HN | 25 | HA | 0.45 | 0.34 | 0.38 | 0.33 | 0.37 |
| 1372 | 28 | HN | 27 | HB1 | 0.45 | 0.34 | 0.39 | 0.30 | 0.38 |
| 1373 | 28 | HN | 29 | HB | 0.55 | 0.49 | 0.52 | 0.50 | 0.48 |
| 1374 | 28 | HN | 29 | HG2@ | 0.75 | 0.54 | 0.54 | 0.53 | 0.55 |
| 1375 | 28 | HN | 30 | HN | 0.55 | 0.42 | 0.43 | 0.43 | 0.43 |
| 1376 | 29 | HN | 28 | HA | 0.45 | 0.35 | 0.35 | 0.35 | 0.35 |
| 1377 | 29 | HN | 30 | HB1 | 0.55 | 0.47 | 0.48 | 0.49 | 0.48 |
| 1378 | 29 | HN | 30 | HB2 | 0.55 | 0.49 | 0.50 | 0.50 | 0.50 |
| 1379 | 29 | HN | 25 | HA | 0.55 | 0.42 | 0.42 | 0.42 | 0.43 |
| 1380 | 29 | HN | 28 | HD1 | 0.55 | 0.47 | 0.45 | 0.48 | 0.45 |
| 1381 | 30 | HN | 31 | HN | 0.30 | 0.28 | 0.30 | 0.30 | 0.31 |
| 1382 | 31 | HN | 27 | HA | 0.45 | 0.41 | 0.39 | 0.38 | 0.42 |
| 1383 | 31 | HN | 29 | HA | 0.45 | 0.43 | 0.46 | 0.46 | 0.44 |
| 1384 | 31 | HN | 30 | HA | 0.45 | 0.35 | 0.35 | 0.35 | 0.35 |
| 1385 | 31 | HN | 33 | HN | 0.55 | 0.42 | 0.44 | 0.44 | 0.43 |
| 1386 | 32 | HN | 30 | HA | 0.55 | 0.44 | 0.46 | 0.45 | 0.47 |
| 1387 | 32 | HN | 29 | HA | 0.45 | 0.34 | 0.35 | 0.35 | 0.33 |
| 1388 | 32 | HN | 31 | HB@ | 0.55 | 0.33 | 0.32 | 0.32 | 0.31 |
| 1389 | 33 | HN | 31 | HA | 0.55 | 0.44 | 0.46 | 0.45 | 0.45 |
| 1390 | 33 | HN | 29 | HA | 0.45 | 0.41 | 0.40 | 0.41 | 0.39 |
| 1391 | 33 | HN | 33 | HB1 | 0.30 | 0.24 | 0.25 | 0.26 | 0.26 |
| 1392 | 33 | HN | 33 | HB2 | 0.30 | 0.26 | 0.23 | 0.23 | 0.23 |
| 1393 | 33 | HN | 33 | HD@ | 0.75 | 0.49 | 0.47 | 0.46 | 0.49 |
| 1394 | 33 | HN | 33 | HG@ | 0.75 | 0.44 | 0.41 | 0.35 | 0.38 |
| 1395 | 33 | HN | 35 | HN | 0.45 | 0.42 | 0.45 | 0.47 | 0.45 |
| 1396 | 34 | HN | 32 | HN | 0.55 | 0.44 | 0.46 | 0.46 | 0.47 |
| 1397 | 34 | HN | 33 | HB1 | 0.45 | 0.26 | 0.24 | 0.26 | 0.23 |
| 1398 | 34 | HN | 33 | HB2 | 0.45 | 0.39 | 0.36 | 0.32 | 0.33 |
| 1399 | 34 | HN | 33 | HG@ | 0.65 | 0.38 | 0.40 | 0.40 | 0.39 |
| 1400 | 35 | HN | 32 | HA | 0.45 | 0.34 | 0.38 | 0.39 | 0.38 |
| 1401 | 35 | HN | 33 | HA | 0.55 | 0.46 | 0.47 | 0.49 | 0.49 |
| 1402 | 35 | HN | 34 | HA | 0.45 | 0.34 | 0.34 | 0.35 | 0.35 |
| 1403 | 35 | HN | 35 | HG@ | 0.50 | 0.32 | 0.32 | 0.38 | 0.31 |
| 1404 | 35 | HN | 37 | HN | 0.45 | 0.39 | 0.39 | 0.50 | 0.38 |
| 1405 | 36 | HN | 32 | HA | 0.45 | 0.36 | 0.31 | 0.31 | 0.28 |
| 1406 | 36 | HN | 32 | HB@ | 0.85 | 0.53 | 0.52 | 0.53 | 0.50 |
| 1407 | 36 | HN | 35 | HB@ | 0.65 | 0.31 | 0.28 | 0.29 | 0.27 |
| 1408 | 36 | HN | 34 | HA | 0.45 | 0.42 | 0.52 | 0.54 | 0.56 |
| 1409 | 38 | HN | 38 | HD@ | 0.89 | 0.35 | 0.33 | 0.34 | 0.33 |
| 1410 | 39 | HN | 38 | HA | 0.45 | 0.30 | 0.29 | 0.31 | 0.30 |
| 1411 | 40 | HN | 39 | HB1 | 0.45 | 0.43 | 0.40 | 0.34 | 0.39 |
| 1412 | 40 | HN | 39 | HB2 | 0.45 | 0.44 | 0.41 | 0.38 | 0.33 |
| 1413 | 40 | HN | 39 | HD@ | 0.75 | 0.55 | 0.50 | 0.34 | 0.47 |
| 1414 | 40 | HN | 41 | HA | 0.55 | 0.53 | 0.52 | 0.51 | 0.53 |
| 1415 | 40 | HN | 41 | HB1 | 0.55 | 0.62 | 0.60 | 0.61 | 0.63 |
| 1416 | 40 | HN | 41 | HB2 | 0.45 | 0.49 | 0.48 | 0.48 | 0.50 |
| 1417 | 41 | HN | 40 | HB | 0.45 | 0.40 | 0.35 | 0.37 | 0.35 |
| 1418 | 41 | HN | 40 | HG2@ | 0.75 | 0.50 | 0.49 | 0.49 | 0.50 |
| 1419 | 41 | HN | 41 | HB@ | 0.50 | 0.29 | 0.28 | 0.28 | 0.28 |
| 1420 | 42 | HN | 39 | HD@ | 0.75 | 0.51 | 0.45 | 0.63 | 0.45 |
| 1421 | 42 | HN | 40 | HG2@ | 0.85 | 0.64 | 0.65 | 0.65 | 0.67 |
| 1422 | 42 | HN | 41 | HA | 0.45 | 0.32 | 0.31 | 0.33 | 0.31 |
| 1423 | 42 | HN | 41 | HB@ | 0.65 | 0.40 | 0.37 | 0.35 | 0.37 |
| 1424 | 42 | HN | 43 | HN | 0.45 | 0.46 | 0.44 | 0.40 | 0.42 |
| 1425 | 43 | HN | 44 | HN | 0.55 | 0.44 | 0.40 | 0.36 | 0.30 |
| 1426 | 44 | HN | 43 | HB | 0.45 | 0.29 | 0.34 | 0.42 | 0.35 |
| 1427 | 44 | HN | 43 | HG2@ | 0.75 | 0.45 | 0.46 | 0.52 | 0.44 |
| 1428 | 46 | HN | 45 | HB@ | 0.65 | 0.39 | 0.39 | 0.35 | 0.37 |
| 1429 | 46 | HN | 46 | HD@ | 0.65 | 0.33 | 0.46 | 0.53 | 0.35 |
| 1430 | 46 | HN | 50 | HN | 0.55 | 0.44 | 0.43 | 0.34 | 0.39 |
| 1431 | 47 | HN | 46 | HB@ | 0.50 | 0.31 | 0.33 | 0.36 | 0.32 |
| 1432 | 48 | HN | 47 | HA | 0.45 | 0.33 | 0.34 | 0.34 | 0.34 |
| 1433 | 48 | HN | 47 | HG2@ | 0.75 | 0.50 | 0.49 | 0.48 | 0.48 |
| 1434 | 48 | HN | 50 | HN | 0.45 | 0.37 | 0.39 | 0.46 | 0.39 |
| 1435 | 49 | HN | 47 | HA | 0.45 | 0.32 | 0.38 | 0.35 | 0.37 |
| 1436 | 49 | HN | 48 | HA | 0.45 | 0.32 | 0.33 | 0.27 | 0.26 |
| 1437 | 49 | HN | 48 | HB1 | 0.55 | 0.44 | 0.41 | 0.45 | 0.44 |
| 1438 | 49 | HN | 48 | HB2 | 0.55 | 0.44 | 0.39 | 0.43 | 0.43 |
| 1439 | 50 | HN | 49 | HA@ | 0.65 | 0.31 | 0.31 | 0.30 | 0.31 |
| 1440 | 51 | HN | 52 | HA | 0.55 | 0.47 | 0.52 | 0.52 | 0.50 |
| 1441 | 51 | HN | 52 | HN | 0.45 | 0.43 | 0.44 | 0.45 | 0.40 |
| 1442 | 52 | HN | 53 | HD@ | 0.99 | 0.55 | 0.58 | 0.58 | 0.60 |
| 1443 | 52 | HN | 53 | HE@ | 0.99 | 0.57 | 0.67 | 0.56 | 0.65 |
| 1444 | 53 | HN | 51 | HG2@ | 0.85 | 0.56 | 0.63 | 0.77 | 0.78 |
| 1445 | 54 | HN | 52 | HA | 0.45 | 0.61 | 0.62 | 0.63 | 0.57 |
| 1446 | 54 | HN | 57 | HN | 0.55 | 0.43 | 0.49 | 0.71 | 0.49 |
| 1447 | 54 | HN | 53 | HB2 | 0.45 | 0.42 | 0.39 | 0.35 | 0.43 |
| 1448 | 54 | HN | 53 | HE@ | 0.99 | 0.69 | 0.64 | 0.68 | 0.68 |
| 1449 | 54 | HN | 53 | HD@ | 0.99 | 0.45 | 0.41 | 0.43 | 0.45 |
| 1450 | 56 | HN | 55 | HA | 0.55 | 0.34 | 0.35 | 0.34 | 0.35 |
| 1451 | 56 | HN | 55 | HB | 0.55 | 0.41 | 0.30 | 0.28 | 0.39 |
| 1452 | 56 | HN | 57 | HA | 0.45 | 0.45 | 0.46 | 0.49 | 0.47 |
| 1453 | 56 | HN | 58 | HN | 0.55 | 0.41 | 0.45 | 0.46 | 0.44 |
| 1454 | 57 | HN | 57 | HG@ | 0.65 | 0.34 | 0.32 | 0.32 | 0.32 |
| 1455 | 60 | HN | 61 | HB@ | 0.75 | 0.50 | 0.53 | 0.53 | 0.56 |
| 1456 | 62 | HN | 61 | HB@ | 0.65 | 0.30 | 0.31 | 0.30 | 0.35 |
| 1457 | 62 | HN | 62 | HB@ | 0.65 | 0.29 | 0.27 | 0.33 | 0.28 |
| 1458 | 62 | HN | 63 | HN | 0.45 | 0.27 | 0.30 | 0.23 | 0.44 |
| 1459 | 63 | HN | 62 | HA | 0.45 | 0.35 | 0.35 | 0.35 | 0.25 |
| 1460 | 63 | HN | 62 | HB@ | 0.65 | 0.35 | 0.26 | 0.36 | 0.26 |
| 1461 | 63 | HN | 63 | HB@ | 0.65 | 0.31 | 0.30 | 0.28 | 0.28 |
| 1462 | 63 | HN | 64 | HN | 0.45 | 0.24 | 0.27 | 0.30 | 0.34 |
| 1463 | 64 | HN | 65 | HN | 0.55 | 0.45 | 0.46 | 0.44 | 0.44 |
| 1464 | 67 | HN | 68 | HN | 0.30 | 0.27 | 0.28 | 0.39 | 0.38 |
| 1465 | 67 | HN | 69 | HN | 0.45 | 0.38 | 0.43 | 0.43 | 0.45 |
| 1466 | 68 | HN | 67 | HA@ | 0.65 | 0.31 | 0.31 | 0.28 | 0.29 |
| 1467 | 68 | HN | 69 | HN | 0.25 | 0.20 | 0.25 | 0.29 | 0.29 |
| 1468 | 69 | HN | 68 | HB@ | 0.65 | 0.40 | 0.31 | 0.38 | 0.33 |
| 1469 | 69 | HN | 70 | HD@ | 0.75 | 0.49 | 0.47 | 0.47 | 0.48 |
| 1470 | 69 | HN | 69 | HA | 0.45 | 0.29 | 0.27 | 0.28 | 0.28 |
| 1471 | 71 | HN | 69 | HG2@ | 0.85 | 0.62 | 0.70 | 0.72 | 0.83 |
| 1472 | 71 | HN | 70 | HB@ | 0.65 | 0.34 | 0.32 | 0.36 | 0.30 |
| 1473 | 72 | HN | 69 | HG2@ | 0.85 | 0.56 | 0.62 | 0.63 | 0.83 |
| 1474 | 72 | HN | 70 | HB@ | 0.75 | 0.55 | 0.56 | 0.59 | 0.62 |
| 1475 | 72 | HN | 73 | HN | 0.45 | 0.46 | 0.45 | 0.29 | 0.29 |
| 1476 | 73 | HN | 73 | HA | 0.45 | 0.29 | 0.27 | 0.26 | 0.27 |
| 1477 | 73 | HN | 73 | HB@ | 0.65 | 0.30 | 0.28 | 0.28 | 0.29 |
| 1478 | 73 | HN | 74 | HN | 0.45 | 0.21 | 0.26 | 0.29 | 0.37 |
| 1479 | 74 | HN | 73 | HA | 0.45 | 0.34 | 0.34 | 0.33 | 0.23 |
| 1480 | 74 | HN | 75 | HD@ | 1.08 | 0.60 | 0.62 | 0.60 | 0.67 |
| 1481 | 74 | HN | 75 | HG | 0.45 | 0.43 | 0.46 | 0.45 | 0.53 |
| 1482 | 75 | HN | 76 | HA | 0.55 | 0.51 | 0.53 | 0.53 | 0.51 |
| 1483 | 75 | HN | 77 | HN | 0.45 | 0.41 | 0.43 | 0.43 | 0.38 |
| 1484 | 77 | HN | 77 | HA | 0.25 | 0.22 | 0.20 | 0.20 | 0.20 |
| 1485 | 77 | HN | 78 | HD@ | 0.75 | 0.65 | 0.68 | 0.77 | 0.75 |
| 1486 | 77 | HN | 78 | HG2@ | 0.85 | 0.61 | 0.66 | 0.62 | 0.59 |
| 1487 | 78 | HN | 79 | HD@ | 0.65 | 0.49 | 0.48 | 0.47 | 0.47 |
| 1488 | 80 | HN | 79 | HB@ | 0.65 | 0.35 | 0.34 | 0.30 | 0.36 |
| 1489 | 80 | HN | 80 | HB1 | 0.45 | 0.35 | 0.35 | 0.35 | 0.32 |
| 1490 | 80 | HN | 80 | HB2 | 0.45 | 0.25 | 0.24 | 0.25 | 0.27 |
| 1491 | 80 | HN | 82 | HN | 0.55 | 0.44 | 0.45 | 0.46 | 0.44 |
| 1492 | 81 | HN | 79 | HA | 0.45 | 0.39 | 0.42 | 0.43 | 0.39 |
| 1493 | 81 | HN | 79 | HB@ | 0.65 | 0.34 | 0.35 | 0.34 | 0.36 |
| 1494 | 81 | HN | 80 | HB1 | 0.45 | 0.39 | 0.36 | 0.38 | 0.38 |
| 1495 | 81 | HN | 80 | HB2 | 0.45 | 0.30 | 0.28 | 0.28 | 0.27 |
| 1496 | 81 | HN | 84 | HD@ | 0.98 | 0.68 | 0.66 | 0.69 | 0.76 |
| 1497 | 82 | HN | 81 | HA | 0.45 | 0.35 | 0.35 | 0.35 | 0.34 |
| 1498 | 82 | HN | 79 | HB@ | 0.65 | 0.41 | 0.34 | 0.40 | 0.35 |
| 1499 | 82 | HN | 83 | HD@ | 0.98 | 0.61 | 0.64 | 0.63 | 0.62 |
| 1500 | 82 | HN | 84 | HN | 0.45 | 0.42 | 0.44 | 0.43 | 0.46 |
| 1501 | 83 | HN | 85 | HN | 0.45 | 0.43 | 0.45 | 0.45 | 0.50 |
| 1502 | 84 | HN | 85 | HN | 0.30 | 0.23 | 0.29 | 0.30 | 0.32 |
| 1503 | 85 | HN | 84 | HB@ | 0.65 | 0.40 | 0.36 | 0.32 | 0.41 |
| 1504 | 85 | HN | 84 | HD@ | 0.98 | 0.59 | 0.57 | 0.53 | 0.59 |
| 1505 | 85 | HN | 84 | HG | 0.45 | 0.48 | 0.48 | 0.44 | 0.49 |
| 1506 | 85 | HN | 86 | HN | 0.55 | 0.47 | 0.45 | 0.45 | 0.43 |
| 1507 | 86 | HN | 87 | HA | 0.55 | 0.52 | 0.53 | 0.51 | 0.51 |
| 1508 | 86 | HN | 87 | HB1 | 0.45 | 0.46 | 0.51 | 0.55 | 0.58 |
| 1509 | 86 | HN | 87 | HB2 | 0.45 | 0.51 | 0.51 | 0.55 | 0.49 |
| 1510 | 88 | HN | 87 | HB1 | 0.55 | 0.41 | 0.39 | 0.44 | 0.43 |
| 1511 | 88 | HN | 88 | HB | 0.45 | 0.37 | 0.27 | 0.27 | 0.25 |
| 1512 | 88 | HN | 89 | HN | 0.45 | 0.27 | 0.26 | 0.32 | 0.28 |
| 1513 | 89 | HN | 88 | HA | 0.45 | 0.31 | 0.32 | 0.35 | 0.32 |
| 1514 | 89 | HN | 87 | HA | 0.45 | 0.41 | 0.40 | 0.39 | 0.34 |
| 1515 | 89 | HN | 90 | HB@ | 0.75 | 0.59 | 0.55 | 0.54 | 0.62 |
| 1516 | 89 | HN | 91 | HN | 0.55 | 0.45 | 0.44 | 0.41 | 0.45 |
| 1517 | 90 | HN | 87 | HA | 0.55 | 0.66 | 0.60 | 0.46 | 0.65 |
| 1518 | 91 | HN | 93 | HN | 0.45 | 0.43 | 0.45 | 0.44 | 0.43 |
| 1519 | 91 | HN | 89 | HA | 0.75 | 0.46 | 0.46 | 0.44 | 0.41 |
| 1520 | 92 | HN | 88 | HD@ | 0.85 | 0.55 | 0.55 | 0.64 | 0.66 |
| 1521 | 92 | HN | 93 | HA | 0.55 | 0.52 | 0.53 | 0.52 | 0.54 |
| 1522 | 93 | HN | 92 | HA | 0.45 | 0.35 | 0.35 | 0.35 | 0.35 |
| 1523 | 94 | HN | 93 | HA | 0.45 | 0.35 | 0.35 | 0.35 | 0.35 |
| 1524 | 94 | HN | 95 | HB@ | 0.75 | 0.53 | 0.54 | 0.54 | 0.55 |
| 1525 | 94 | HN | 92 | HA | 0.55 | 0.43 | 0.46 | 0.46 | 0.45 |
| 1526 | 95 | HN | 93 | HA | 0.45 | 0.45 | 0.46 | 0.45 | 0.45 |
| 1527 | 96 | HN | 95 | HA | 0.45 | 0.35 | 0.35 | 0.35 | 0.35 |
| 1528 | 96 | HN | 92 | HA | 0.55 | 0.40 | 0.40 | 0.41 | 0.40 |
| 1529 | 96 | HN | 94 | HN | 0.45 | 0.41 | 0.45 | 0.45 | 0.45 |
| 1530 | 96 | HN | 96 | HD@ | 0.65 | 0.44 | 0.43 | 0.44 | 0.45 |
| 1531 | 96 | HN | 96 | HG@ | 0.75 | 0.30 | 0.28 | 0.30 | 0.36 |
| 1532 | 96 | HN | 94 | HA | 0.55 | 0.42 | 0.44 | 0.44 | 0.45 |
| 1533 | 97 | HN | 96 | HG@ | 0.75 | 0.47 | 0.46 | 0.44 | 0.42 |
| 1534 | 97 | HN | 97 | HB@ | 0.50 | 0.23 | 0.28 | 0.28 | 0.28 |
| 1535 | 97 | HN | 93 | HA | 0.55 | 0.43 | 0.45 | 0.48 | 0.44 |
| 1536 | 97 | HN | 96 | HA | 0.45 | 0.35 | 0.35 | 0.35 | 0.35 |
| 1537 | 98 | HN | 99 | HB | 0.55 | 0.50 | 0.64 | 0.64 | 0.54 |
| 1538 | 98 | HN | 99 | HG@ | 1.08 | 0.62 | 0.55 | 0.54 | 0.60 |
| 1539 | 98 | HN | 100 | HN | 0.55 | 0.42 | 0.43 | 0.45 | 0.45 |
| 1540 | 98 | HN | 96 | HA | 0.55 | 0.45 | 0.45 | 0.46 | 0.46 |
| 1541 | 99 | HN | 98 | HA | 0.45 | 0.35 | 0.35 | 0.35 | 0.35 |
| 1542 | 99 | HN | 97 | HA | 0.45 | 0.43 | 0.46 | 0.47 | 0.45 |
| 1543 | 100 | HN | 97 | HN | 0.55 | 0.47 | 0.49 | 0.56 | 0.48 |
| 1544 | 100 | HN | 99 | HG@ | 0.98 | 0.41 | 0.41 | 0.44 | 0.40 |
| 1545 | 101 | HN | 100 | HA | 0.45 | 0.34 | 0.34 | 0.35 | 0.35 |
| 1546 | 102 | HN | 100 | HA | 0.55 | 0.33 | 0.63 | 0.42 | 0.37 |
| 1547 | 102 | HN | 101 | HB@ | 0.65 | 0.40 | 0.37 | 0.42 | 0.40 |
| 1548 | 102 | HN | 103 | HA | 0.55 | 0.49 | 0.53 | 0.56 | 0.53 |
| 1549 | 103 | HN | 99 | HG@ | 0.98 | 0.57 | 0.93 | 0.80 | 0.65 |
| 1550 | 103 | HN | 102 | HA@ | 0.65 | 0.31 | 0.31 | 0.30 | 0.31 |
| 1551 | 103 | HN | 103 | HD@ | 0.75 | 0.26 | 0.33 | 0.50 | 0.40 |
| 1552 | 104 | HN | 103 | HA | 0.30 | 0.23 | 0.34 | 0.24 | 0.24 |
| 1553 | 104 | HN | 106 | HN | 0.55 | 0.40 | 0.48 | 0.54 | 0.53 |
| 1554 | 105 | HN | 105 | HG@ | 0.75 | 0.28 | 0.32 | 0.35 | 0.31 |
| 1555 | 105 | HN | 106 | HB1 | 0.55 | 0.61 | 0.50 | 0.60 | 0.64 |
| 1556 | 105 | HN | 106 | HB2 | 0.55 | 0.46 | 0.57 | 0.47 | 0.51 |
| 1557 | 105 | HN | 106 | HN | 0.30 | 0.28 | 0.31 | 0.27 | 0.30 |
| 1558 | 105 | HN | 107 | HN | 0.45 | 0.45 | 0.57 | 0.45 | 0.48 |
| 1559 | 105 | HN | 105 | HE@ | 0.60 | 0.59 | 0.60 | 0.53 | 0.39 |
| 1560 | 107 | HN | 106 | HB1 | 0.45 | 0.34 | 0.37 | 0.38 | 0.43 |
| 1561 | 107 | HN | 106 | HB2 | 0.45 | 0.32 | 0.35 | 0.32 | 0.40 |
| 1562 | 107 | HN | 108 | HE3 | 0.45 | 0.37 | 0.34 | 0.44 | 0.49 |
| 1563 | 107 | HN | 108 | HZ3 | 0.45 | 0.46 | 0.43 | 0.53 | 0.59 |
| 1564 | 108 | HN | 106 | HA | 0.45 | 0.37 | 0.39 | 0.37 | 0.35 |
| 1565 | 108 | HN | 109 | HN | 0.45 | 0.43 | 0.44 | 0.44 | 0.44 |
| 1566 | 109 | HN | 108 | HA | 0.45 | 0.22 | 0.21 | 0.23 | 0.23 |
| 1567 | 109 | HN | 108 | HB@ | 0.65 | 0.39 | 0.39 | 0.29 | 0.32 |
| 1568 | 109 | HN | 110 | HB@ | 0.75 | 0.56 | 0.54 | 0.54 | 0.56 |
| 1569 | 110 | HN | 108 | HA | 0.45 | 0.39 | 0.40 | 0.45 | 0.45 |
| 1570 | 110 | HN | 108 | HB@ | 0.65 | 0.39 | 0.42 | 0.37 | 0.36 |
| 1571 | 110 | HN | 112 | HN | 0.45 | 0.43 | 0.46 | 0.45 | 0.44 |
| 1572 | 111 | HN | 108 | HA | 0.55 | 0.43 | 0.44 | 0.53 | 0.47 |
| 1573 | 111 | HN | 112 | HB@ | 0.65 | 0.57 | 0.53 | 0.52 | 0.55 |
| 1574 | 111 | HN | 110 | HA | 0.45 | 0.35 | 0.35 | 0.35 | 0.34 |
| 1575 | 111 | HN | 110 | HB@ | 0.75 | 0.36 | 0.34 | 0.34 | 0.35 |
| 1576 | 112 | HN | 111 | HA | 0.45 | 0.36 | 0.35 | 0.34 | 0.34 |
| 1577 | 112 | HN | 110 | HA | 0.45 | 0.44 | 0.46 | 0.45 | 0.44 |
| 1578 | 112 | HN | 112 | HG@ | 0.65 | 0.27 | 0.27 | 0.27 | 0.26 |
| 1579 | 113 | HN | 110 | HA | 0.45 | 0.32 | 0.38 | 0.38 | 0.41 |
| 1580 | 113 | HN | 112 | HA | 0.45 | 0.37 | 0.35 | 0.35 | 0.30 |
| 1581 | 113 | HN | 114 | HB1 | 0.55 | 0.57 | 0.58 | 0.70 | 0.59 |
| 1582 | 113 | HN | 114 | HB2 | 0.55 | 0.49 | 0.50 | 0.58 | 0.57 |
| 1583 | 113 | HN | 115 | HN | 0.45 | 0.41 | 0.44 | 0.44 | 0.37 |
| 1584 | 113 | HN | 109 | HA | 0.55 | 0.44 | 0.41 | 0.37 | 0.55 |
| 1585 | 114 | HN | 111 | HA | 0.55 | 0.37 | 0.40 | 0.65 | 0.53 |
| 1586 | 114 | HN | 116 | HN | 0.45 | 0.38 | 0.54 | 0.46 | 0.45 |
| 1587 | 115 | HN | 114 | HA | 0.45 | 0.35 | 0.33 | 0.33 | 0.34 |
| 1588 | 115 | HN | 116 | HB1 | 0.55 | 0.38 | 0.66 | 0.61 | 0.49 |
| 1589 | 115 | HN | 116 | HB2 | 0.55 | 0.43 | 0.57 | 0.50 | 0.47 |
| 1590 | 115 | HN | 116 | HN | 0.30 | 0.20 | 0.37 | 0.30 | 0.27 |
| 1591 | 115 | HN | 111 | HA | 0.55 | 0.34 | 0.28 | 0.44 | 0.34 |
| 1592 | 116 | HN | 115 | HB@ | 0.65 | 0.29 | 0.37 | 0.26 | 0.36 |
| 1593 | 116 | HN | 116 | HG@ | 0.65 | 0.42 | 0.32 | 0.29 | 0.37 |
| 1594 | 116 | HN | 113 | HN | 0.55 | 0.49 | 0.70 | 0.54 | 0.38 |
| 1595 | 117 | HN | 116 | HG@ | 0.65 | 0.34 | 0.44 | 0.50 | 0.36 |
| 1596 | 118 | HN | 116 | HA | 0.45 | 0.36 | 0.49 | 0.46 | 0.40 |
| 1597 | 118 | HN | 117 | HA@ | 0.65 | 0.32 | 0.28 | 0.29 | 0.31 |
| 1598 | 119 | HN | 120 | HN | 0.45 | 0.39 | 0.44 | 0.27 | 0.38 |
| 1599 | 120 | HN | 119 | HB1 | 0.45 | 0.44 | 0.35 | 0.40 | 0.38 |
| 1600 | 120 | HN | 119 | HB2 | 0.45 | 0.46 | 0.40 | 0.40 | 0.32 |
| 1601 | 120 | HN | 120 | HB | 0.45 | 0.36 | 0.25 | 0.34 | 0.29 |
| 1602 | 120 | HN | 122 | HN | 0.45 | 0.44 | 0.49 | 0.47 | 0.47 |
| 1603 | 120 | HN | 123 | HN | 0.45 | 0.57 | 0.55 | 0.56 | 0.55 |
| 1604 | 121 | HN | 121 | HG@ | 0.65 | 0.44 | 0.31 | 0.32 | 0.33 |
| 1605 | 122 | HN | 119 | HA | 0.55 | 0.46 | 0.55 | 0.42 | 0.49 |
| 1606 | 122 | HN | 121 | HB@ | 0.65 | 0.35 | 0.30 | 0.33 | 0.32 |
| 1607 | 122 | HN | 121 | HG@ | 0.65 | 0.50 | 0.46 | 0.43 | 0.50 |
| 1608 | 123 | HN | 124 | HD@ | 0.85 | 0.70 | 0.66 | 0.64 | 0.66 |
| 1609 | 123 | HN | 124 | HG2@ | 0.85 | 0.55 | 0.58 | 0.59 | 0.65 |
| 1610 | 123 | HN | 125 | HB@ | 0.75 | 0.52 | 0.62 | 0.72 | 0.75 |
| 1611 | 124 | HN | 121 | HA | 0.45 | 0.34 | 0.37 | 0.38 | 0.39 |
| 1612 | 124 | HN | 122 | HA | 0.45 | 0.40 | 0.47 | 0.48 | 0.43 |
| 1613 | 124 | HN | 123 | HA | 0.45 | 0.34 | 0.35 | 0.35 | 0.34 |
| 1614 | 124 | HN | 125 | HB@ | 0.65 | 0.42 | 0.55 | 0.61 | 0.62 |
| 1615 | 125 | HN | 122 | HA | 0.45 | 0.33 | 0.41 | 0.55 | 0.54 |
| 1616 | 125 | HN | 124 | HA | 0.45 | 0.31 | 0.29 | 0.23 | 0.23 |
| 1617 | 125 | HN | 124 | HD@ | 0.75 | 0.57 | 0.52 | 0.64 | 0.66 |
| 1618 | 125 | HN | 124 | HB | 0.45 | 0.44 | 0.40 | 0.41 | 0.41 |
| 1619 | 125 | HN | 123 | HB1 | 0.55 | 0.55 | 0.55 | 0.54 | 0.54 |
| 1620 | 125 | HN | 123 | HB2 | 0.55 | 0.52 | 0.52 | 0.54 | 0.56 |
| 1621 | 126 | HN | 127 | HB1 | 0.55 | 0.62 | 0.63 | 0.64 | 0.62 |
| 1622 | 126 | HN | 127 | HB2 | 0.55 | 0.50 | 0.53 | 0.58 | 0.66 |
| 1623 | 127 | HN | 124 | HA | 0.45 | 0.44 | 0.59 | 0.50 | 0.52 |
| 1624 | 127 | HN | 124 | HB | 0.55 | 0.45 | 0.63 | 0.43 | 0.43 |
| 1625 | 127 | HN | 126 | HA@ | 0.65 | 0.32 | 0.28 | 0.30 | 0.29 |
| 1626 | 127 | HN | 129 | HD@ | 0.98 | 0.57 | 0.83 | 0.79 | 0.79 |
| 1627 | 128 | HN | 129 | HD@ | 1.08 | 0.59 | 0.69 | 0.60 | 0.63 |
| 1628 | 129 | HN | 127 | HB1 | 0.55 | 0.52 | 0.56 | 0.44 | 0.51 |
| 1629 | 129 | HN | 127 | HB2 | 0.55 | 0.51 | 0.57 | 0.45 | 0.41 |
| 1630 | 129 | HN | 128 | HB@ | 0.65 | 0.40 | 0.33 | 0.42 | 0.42 |
